# Hox transcript antisense RNA knockdown inhibits osteosarcoma progression by regulating the phosphoinositide 3-kinase/AKT pathway through the microRNA miR-6888-3p/spleen tyrosine kinase axis

**DOI:** 10.1080/21655979.2022.2059614

**Published:** 2022-04-17

**Authors:** Wei Wu, Linxiu Wang, Sen Li

**Affiliations:** a College of Integration of Traditional Chinese and Western Medicine, Southwest Medical University, Luzhou, Sichuan, China; b Department of Orthopedics, The Second Affiliated Hospital of Chengdu Medical College, China National Nuclear Corporation 416 Hospital, Chengdu, Sichuan, China; c Department of Spinal Surgery, Chinese Medicine Hospital Affiliated to Southwest Medical University, Luzhou, Sichuan, China

**Keywords:** HOTAIR, osteosarcoma, miR-6888-3p, SYK, PI3K/AKT pathway

## Abstract

Long non-coding RNA HOX transcript antisense RNA (lncRNA HOTAIR) is thought to be a key regulator of the occurrence and development of osteosarcoma (OS). The expression of HOTAIR, microRNA miR-6888-3p, spleen tyrosine kinase (SYK), and phosphoinositide 3-kinase/AKT (PI3K/AKT) pathway-related proteins in OS was detected by quantitative reverse transcription-PCR (qRT-PCR) and western blotting. Changes in the proliferation and migration of OS cells were detected by Cell Counting Kit-8 (CCK-8) and transwell assays after the knockdown of HOTAIR, miR-6888-3p, or SYK. Luciferase assays, RNA immunoprecipitation (RIP), and RNA pull-down assays were used to detect the relationship between miR-6888-3p and HOTAIR or SYK. We found that HOTAIR and SYK were highly expressed in OS, whereas miR-6888-3p expression was low. In addition, downregulation of HOTAIR or SYK significantly inhibited the growth and migration of OS cells and the PI3K/AKT pathway, both *in vitro* and *in vivo*. Additionally, downregulation of miR-6888-3p promoted the proliferation and migration of OS cells and activated the PI3K/AKT pathway. Mechanistically, these results suggest that the HOTAIR sponge, miR-6888-3p, regulates SYK expression. To summarize, HOTAIR regulates SYK by acting on miR-6888-3p, thereby promoting the proliferation and migration of OS cells.

## Graphical abstract

**Figure:**
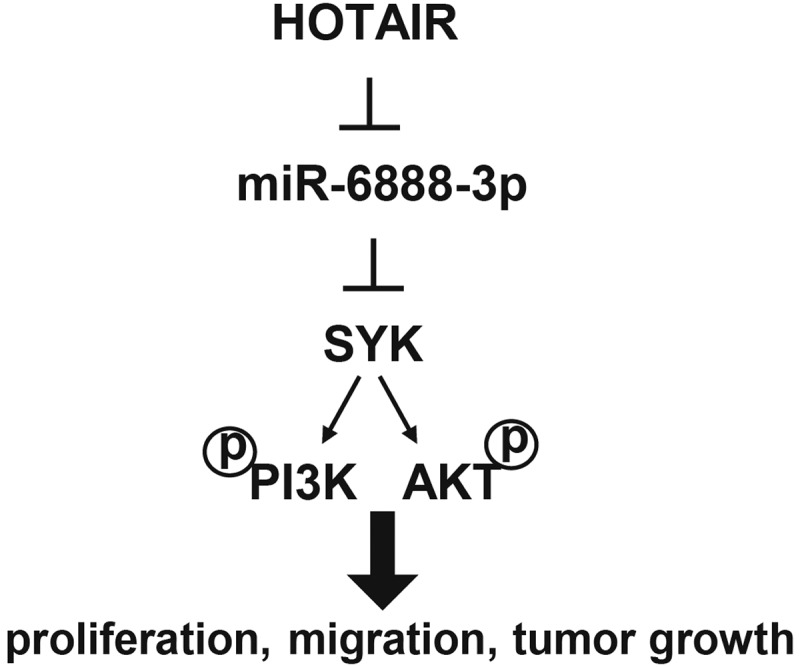

## Introduction

Osteosarcoma (OS) is an aggressive bone malignancy that occurs primarily in the second decade of life and in adults over the age of 40 years [1]. Current treatments for OS mainly include surgical resection and multiple chemotherapy that improves the 5-year survival rate from 20% to 70% [2]. Despite these advances, the overall survival rate of patients with metastatic or recurrent OS is approximately 20% [3,4]. Therefore, informative biomarkers are required to better diagnose and treat OS patients [5].

Long non-coding RNAs (lncRNAs), which are highly tissue-specific, disease-specific, and developmental stage-specific non-coding transcripts, play a key role in biological processes by participating in almost all known levels of regulation to control cis and/or trans gene expression [6]. Accumulating evidence suggests that a combination of lncRNAs may be a better prognostic indicator and adjuvant treatment strategy for OS [7]. Hox transcript antisense RNA (HOTAIR) is highly expressed in a wide range of cancers and is a prime example of a carcinogenic trans-lncRNA [8]. Several studies have revealed that HOTAIR is upregulated in OS, promoting the malignant growth of OS cells, and its genetic variation correlates with OS risk [9,10]. However, the mechanism of HOTAIR in OS remains unclear.

By acting as a sponge, lncRNAs compete for other genes to bind to microRNAs (miRNAs), thereby blocking the transcriptional degradation and translational inhibition of miRNA targeted mRNAs [11]. Previous studies indicate that various miRNAs play carcinogenic or tumor-suppressive roles in the occurrence and development of OS. For example, miR-221 expression is significantly upregulated in OS, indicating a late tumor stage, metastasis, and poor prognosis [12]. Downregulation of miR-451, which is common in clinically advanced OS, is an adverse prognostic factor for overall disease-free survival and inhibiting cancer cell growth *in vitro* and *in vivo* [13]. miR-200a inhibits OS cell proliferation, promotes apoptosis, and increases cellular radiosensitivity [14]. miR-6888-3p is poorly studied and no studies have been conducted on the potential role of HOTAIR/miR-6888-3p in OS progression.

Spleen tyrosine kinase (SYK) mediates signal transduction by immune receptors and is widely expressed in various cell types [15]. Studies have shown that SYK plays a varied role in epithelial cancer. The abundance of SYK is negatively correlated with breast cancer progression and inhibits tumor growth and metastasis in xenografts [16]. In contrast, higher levels of SYK in head and neck squamous cell carcinomas promote cell migration [17]. In addition, an earlier study showed that SYK is expressed in osteoblasts and recruited by genes related to OS [18]. However, the effects and mechanism of action of SYK in OS have rarely been studied.

The phosphoinositide 3-kinase/AKT (PI3K/AKT) pathway is involved in various biological processes and is often abnormally activated in human cancers [19]. Growing evidence suggests that PI3K/AKT signaling directly or indirectly regulates important epigenetic modifications and is involved in the PI3K cascade-related tumorigenicity in cancer [20]. HOTAIR and SYK have been revealed to be the regulators of the PI3K/AKT pathway in adenocarcinoma of esophagogastric junction and HepG2 cells [21,22]. Therefore, the effect of the HOTAIR/miR-6888-3p/SYK axis on the PI3K/AKT pathway warrants further study.

This study investigated the effect of HOTAIR/miR-6888-3p/SYK on the malignant behavior of OS. We hypothesized that HOTAIR sponges miR-6888-3p and upregulates SYK to promote the survival and migration of OS by regulating the PI3K/AKT signaling pathway. This study aimed to provide a promising new strategy for RNA-based diagnosis and treatment of OS.

## Methods

### Tissue specimens

Thirty-two pairs of OS tissues and adjacent normal bone were obtained from patients undergoing surgery at The Second Affiliated Hospital of Chengdu Medical College, China National Nuclear Corporation 416 Hospital, between January 2020 and January 2021. The inclusion criteria were as follows [1]: patients with OS confirmed by pathological examination and [2] patients who understood the principle of the experiment and signed the informed consent. The exclusion criteria were as follows [1]: patients with other diseases, such as other types of malignant tumors and [2] patients who had received treatment before admission. This study was approved by the Ethics Committee of The Second Affiliated Hospital of Chengdu Medical College, China National Nuclear Corporation 416 Hospital, and all patients provided written informed consent. Tissue specimens were immediately frozen in liquid nitrogen and stored at −80°C until use.

### Cell culture

OS cell lines (HOS, Saos2, MG-63, and SW1353) and osteoblasts (HFOB1.19) were purchased from ATCC (USA) and cultured in RPMI-1640 medium (Invitrogen, USA) supplemented with 1% penicillin/streptomycin and 10% fetal bovine serum (FBS) (Gibco, USA). All cells were incubated at 37°C in a humidified atmosphere containing 5% CO_2_.

### Quantitative reverse transcription-PCR assay

Total RNA was extracted from tissues and cells using an RNA isolation kit (Takara, Japan) and then reverse transcribed to cDNA using PrimeScript RT polymerase (Takara). Quantitative reverse transcription-PCR (qRT-PCR) was performed using FastStart Universal SYBR-Green Master Mix (Roche Diagnostics, USA) according to the instructions by the manufacturer, on a 7900 Fast Real-Time PCR system (Applied Biosystems, USA). GAPDH was used as an internal control for normalization.

The miRNAs in tissues and cells were extracted using the NucleoSpin® miRNA kit (Macherey Nagel, France). The ImProm-II Reverse Transcription System (Promega, USA) was used to reverse the RNA to cDNA. Subsequently, TransStart Eco Green qPCR SuperMix (TransGen Biotech, China) was used for qRT-PCR. miRNAs were normalized to U6. The 2^−ΔΔCt^ method was used to calculate the gene expression [23]. The primer sequences are listed in Table 1.

**Table 1.** Sequence of the primers used in this study

Gene	Primer sequence (5'-3')
HOTAIR	Forward: 5'-GGGTGTTGGTCTGTGGAACT-3'
	Reverse: 5'-CAGTGG-GGAACTCTGACTCG-3'
miR-197-3p	Forward: 5'-CACCACCTTCTCCACCCA-3'
	Reverse: 5'-GGGACTGGACTTGGAGTC-3'
FLT1	Forward: 5'-CAGCGGCTTTTGTGGAAG-ACTCAC-3'
	Reverse: 5'-ACATCTCGGTGTCACTT-CTTGGAC-3'
SYK	Forward: 5'-GGAACTGTGAAAAAGGGCTA-3'
	Reverse: 5'-CTGTGCACAAAATTGCTCTC-3'
KDR	Forward: 5'-TGCCTACCTCACCTGTTTC-3'
	Reverse: 5'-GGCTCTTTCGCTTACTGTTC-3'
U6	Forward: 5'-CTCGCTTCGGCAGCACA-3'
	Reverse: 5'-AACGCTTCACGAATTTGCGT-3'
GAPDH	Forward: 5'-CTTTGGTATCGTGGAAGGACTC-3'
	Reverse: 5'-GTAGAGGCAGGGATGATGTTCT-3'

### Subcellular localization

The components of HOS and Saos2 cells were divided into nuclear and cytoplasmic components using the PARIS kit (Ca# AM1921; Thermo Fisher Scientific, USA). Briefly, the nuclear and cytoplasmic components were separated by centrifugation after cell lysis. The supernatant was then collected in RNase-free tubes, and the precipitate was lysed with the cell division buffer. The subcellular localization of HOTAIR was detected by qRT-PCR after the elution of nuclear and cytoplasmic RNA. U6 was used as a positive control for the nuclear component, and GAPDH was used as a positive control for the cytoplasmic component [24].

### Fluorescent in situ hybridization

Fluorescent In Situ Hybridization (FISH) assay was performed using the lncRNA FISH probe (RiboBio, China) and a fluorescent in situ hybridization kit (Genepharma, China) to localize HOTAIR in Saos-2 and HOS cells. The probe cocktail included a HOTAIR probe (the nucleus was visualized using DAPI and it appeared as a blue signal; the HOTAIR gene appeared as a red signal). Briefly, the cells were fixed in 4% paraformaldehyde for 10 min at 25°C. Cells and tissues were permeabilized with Triton-100 for 5 min at 4°C. Hybridization of HOTAIR probes and DAPI were carried out overnight in a humidified chamber at 37°C in the dark, followed by washing in PBS three times every 5 min. Fluorescence signals were captured using an Olympus laser confocal microscope FV1000 (Olympus, Tokyo, Japan) [25].

### Cell transfection

Small interfering RNA targeting HOTAIR or SYK (si-lnc or si-SYK) and a negative control (si-NC) were synthesized by Invitrogen. The miR-6888-3p inhibitor, inhibitor-NC, miR-6888-3p mimic, and mimic-NC were purchased from Switchgear Genomics (USA). HOS and Saos2 cells were transfected with 50 nM siRNA, 75 nM inhibitor, or 75 nM mimic using Lipofectamine 3000 (Invitrogen) according to the manufacturer’s instructions. Knockdown efficiency was determined by qRT-PCR 48 h post-transfection.

### Cell counting kit-8 (CCK-8) assay

Forty-eight hours after transfection, HOS and Saos2 cells (2 × 10^4^ cells/well) were harvested, and seeded into 48-well plates. Cell viability was assessed using the CCK-8 kit (Dojindo, Kumamoto, Japan) at 24, 48, 72, and 96 h after incubation, according to the instructions by the manufacturer. Twenty microliters of CCK-8 solution was added to each well and incubated at 37°C for 2 h. Absorbance was measured at 450 nm using a microplate reader (BioTek, USA).

### Cell migration assay

Cell migration was assessed using Transwell inserts (Corning, USA). Cells (2 × 10^5^) filled with 200 μL serum-free medium were seeded into the upper chamber, and 800 μL of medium containing 20% FBS was added into the lower chamber. After culturing at 37°C for 24 h, the cells on the surface of the filter membrane were fixed with 4% paraformaldehyde for 15 min, stained with 0.5% crystal violet for 10 min, and then visualized using a CKX41 inverted microscope (Olympus, Japan).

### Western blotting

Cellular proteins were extracted with radioimmunoprecipitation assay (RIPA) lysis buffer, and the equivalent protein lysates were separated by 10% sulfate-polyacrylamide gel electrophoresis and transferred to a polyvinylidene fluoride membrane. After blocking the membrane with a blocking buffer for 1 h at 37°C, the membrane was incubated with PI3K (Ca# 3011; 1:1000, Cell Signaling Technology, USA), p-PI3K (Ca# 13857; 1:1000, Cell Signaling Technology), AKT (Ca# 4685; 1:1000, Cell Signaling Technology), p-AKT (Ca# 4060; 1:2000, Cell Signaling Technology), and GAPDH (Ca# ab181602; 1:1000, Abcam, UK) overnight at 4°C. The membrane was then incubated with the secondary antibody and the band signal was detected using the SuperSignal West Pico chemiluminescent substrate kit (Pierce, USA).

### Xenograft model antitumor assay

The animal experiments were approved by the Animal Research Committee of our hospital. Four-week-old male BALB/c mice (10 mice, 18–22 g) were randomly divided into two groups. Short hairpin-targeted HOTAIR (sh-lnc) and negative control (sh-NC) purchased from GenePharma (USA) were packaged into HOS cells. Then, the cells (1 × 10^7^) from the sh-NC and sh-lnc groups were injected subcutaneously into the armpits of mice, with five mice in each group. Tumor volume was measured weekly using a caliper, and all mice were sacrificed after 5 weeks following xenografting. Xenografts were then removed and weighed.

### Luciferase assay

Potential binding sites of miR-6888 in HOTAIR or SYK were predicted using the shared site. The sequences containing wild-type (WT) and mutated (MUT) HOTAIR or SYK 3’-UTR were synthesized from GenePharma (USA) and subcloned into the pMIR-REPORTTM vector (Thermo Fisher Scientific) to construct a luciferase reporter vector. HOS and Saos2 cells were transfected with an miR-6888 mimic and WT or MUT, respectively. Forty-eight hours after transfection, the dual-luciferase assay kit (Yeasen, China) was used to determine the luciferase activity.

### RNA immunoprecipitation assay

RNA immunoprecipitation (RIP) experiment was performed using an EZMagna RIP kit (Millipore, USA). HOS and Saos2 cells transfected with miR-6888 mimic or mimic-NC were lysed using 100 µL RIP lysis buffer containing 0.5 µL protease inhibitor and 0.25 µL RNase inhibitor. The cell lysate was centrifuged at 4°C for 15 min at 513 × *g*, and the supernatant was incubated with RIP buffer containing protein A/G-Sepharose beads conjugated with anti-AGO2 (Ca# 03–110; 1:5,000, Millipore) or negative control IgG antibody (Ca# 12–371; 1:5,000, Millipore). Finally, RNA was eluted from the magnetic beads and analyzed using RT-qPCR [26].

### RNA pull-down assay

Biotin-labeled miR-6888 (Bio-miR-6888) or NC (Bio-NC) (Thermo Fisher Scientific, USA) were transfected into HOS and Saos2 cells. After 24 h, the cells were lysed and incubated with streptavidin beads (Sigma, USA) at 4°C for 4 h. Next, the beads were washed with lysis buffer containing proteinase K (Invitrogen, USA) to collect the supernatant. RNA was extracted and HOTAIR enrichment was assessed using qRT-PCR, as described previously [27].

### Statistical analysis

Student’s t-test and one-way analysis of variance (ANOVA) were used to estimate the statistical significance of the differences between the groups. Statistical significance was set at *p* < 0.05, and the results were expressed as the mean ± standard deviation (SD). All experiments were repeated at least three times. SPSS 18.0 (SPSS, USA) was used for the statistical analysis. The correlation between miR-6888-3p and HOTAIR or SYK expression was analyzed using Pearson’s correlation analysis.

## Results

This study investigated the effect of HOTAIR/miR-6888-3p/SYK on the malignant behavior of OS. We found that HOTAIR was upregulated in OS, promoting the proliferation and migration of cancer cells and activating the PI3K/AKT pathway. Mechanistically, HOTAIR sponges miR-6888-3p and upregulates SYK to promote the survival and migration of OS by activating the PI3K/AKT signaling pathway.

### HOTAIR was upregulated in OS, promoting cancer cell proliferation and migration and activating PI3K/AKT pathway

**Figure 1.**
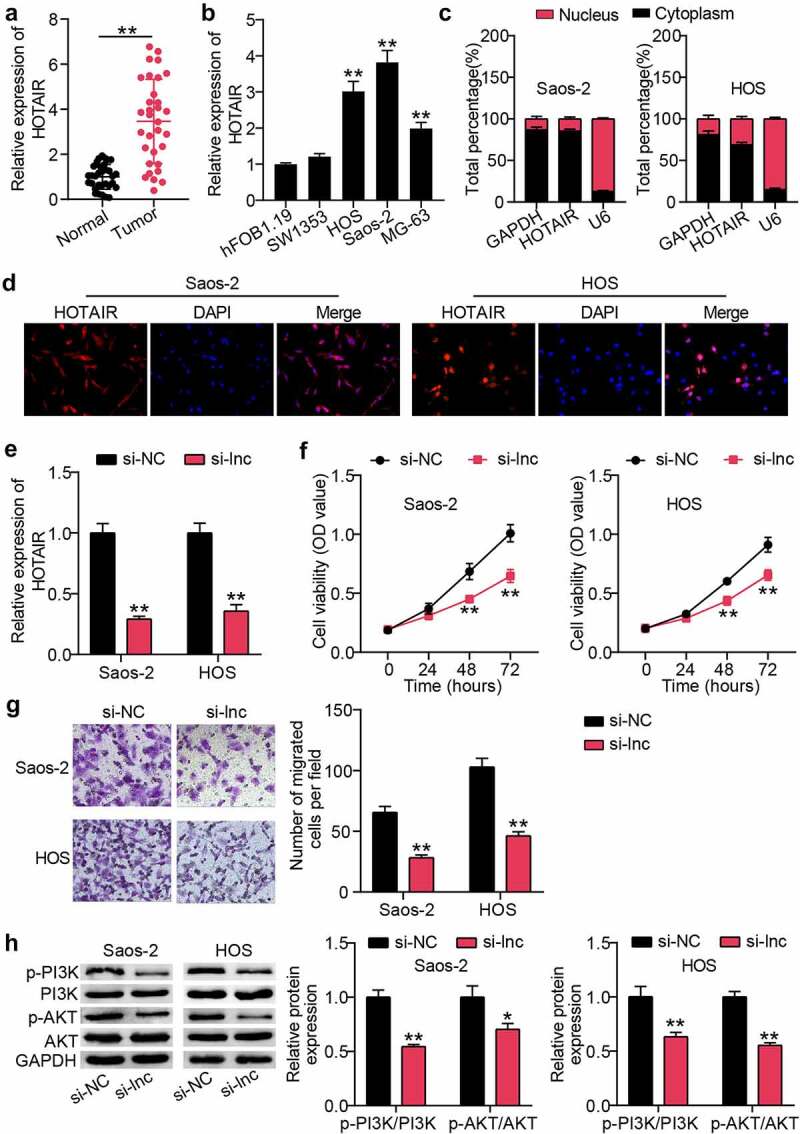
HOTAIR was up-regulated in OS, promoting proliferation and migration of cancer cells, and activating PI3K/AKT pathway (a) qRT-PCR for relative HOTAIR expression in OS tissues and adjacent tissues. ** P < 0.001. (b) qRT-PCR for HOTAIR expression in OS cell lines. ** P < 0.001. vs hFOB1.19. (c) qRT-PCR for HOTAIR expression in cytoplasmic and nuclear of HOS and Saos2 cells. (d) FISH assay showing the localization of HOTAIR in HOS and Saos2 cells. (e) HOS and Saos2 were transfected with si-NC, and si-lnc. The expression of HOTAIR was measured by RT-qPCR. (f) Cell viability was detected by CCK-8 assay in HOS and Saos2 cells transfected with si-NC, and si-lnc. (g) Migrated cells were counted by transwell assay in HOS and Saos2 cells transfected with si-NC, and si-lnc. (h) The expression of PI3K, AKT, p-PI3K and p-AKT protein levels was detected by Western blot analysis in HOS and Saos2 cells transfected with si-NC, and si-lnc. * P < 0.05, ** P < 0.001. vs si-NC treatment.

To determine the role of HOTAIR in OS tissues, qRT-PCR was used to monitor HOTAIR expression in OS and para-cancerous tissues. This concluded that HOTAIR expression was upregulated approximately three-fold in cancer tissues (Figure 1(a)). Similarly, monitoring OS cell lines revealed elevated HOTAIR levels in both cancer cells compared to those in HFOB1.19 cells (Figure 1(b)). HOS and Saos2 cells with higher HOTAIR expression were screened for nuclear-cytoplasmic separation, and the results showed that HOTAIR expression was higher in the cytoplasm than in the nucleus (Figure 1(c)). Similarly, the FISH assay showed that the red fluorescence intensity targeting HOTAIR was mainly localized to the cytoplasm (Figure 1(d)). Furthermore, HOTAIR was knocked down in HOS and Saos2 cells by si-lnc transfection, and qRT-PCR showed that the HOTAIR level in the si-lnc group was approximately 40% of that in the si-NC group (Figure 1(e)). Next, we explored the effects of HOTAIR on the physiological functions of OS cells. CCK-8 results revealed that cell viability in the si-lnc group decreased by approximately 35% compared to that in the si-NC group (figure 1(f)). Cell migration measurements showed that silencing HOTAIR in the Transwell experiment reduced cell migration by more than 50% (Figure 1(g)). Furthermore, western blot analysis revealed that HOTAIR interference inhibited the PI3K/AK pathway, as demonstrated by decreased p-PI3K and p-AKT protein levels (Figure 1(h)).

### *HOTAIR knockdown inhibited the growth of OS cells* in vivo

**Figure 2.**
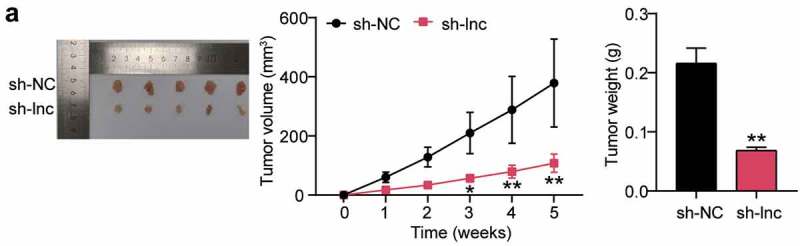
HOTAIR knockdown inhibited the growth of OS cells in vivo (a) Representative images of the tumors from the sh-NC (n = 5) and sh-lnc groups (n = 5) at the end of the experiment. Tumor volume of the mice over the course of the experiment. Body weight of the mice at the end of the experiment. * P < 0.05, ** P < 0.001. vs sh-NC treatment.

To observe the effect of HOTAIR on tumor growth, HOS cells were injected into nude mice for tumor xenotransplantation. Tumor volume was measured weekly, and downregulation of HOTAIR significantly delayed tumor growth *in vivo*. Additionally, tumor weight measurements showed a reduction in tumor size. Conclusively, HOTAIR knockdown significantly inhibited OS cell growth *in vivo* (Figure 2(a)).

### miR-6888-3p: a potential target for HOTAIR

**Figure 3.**
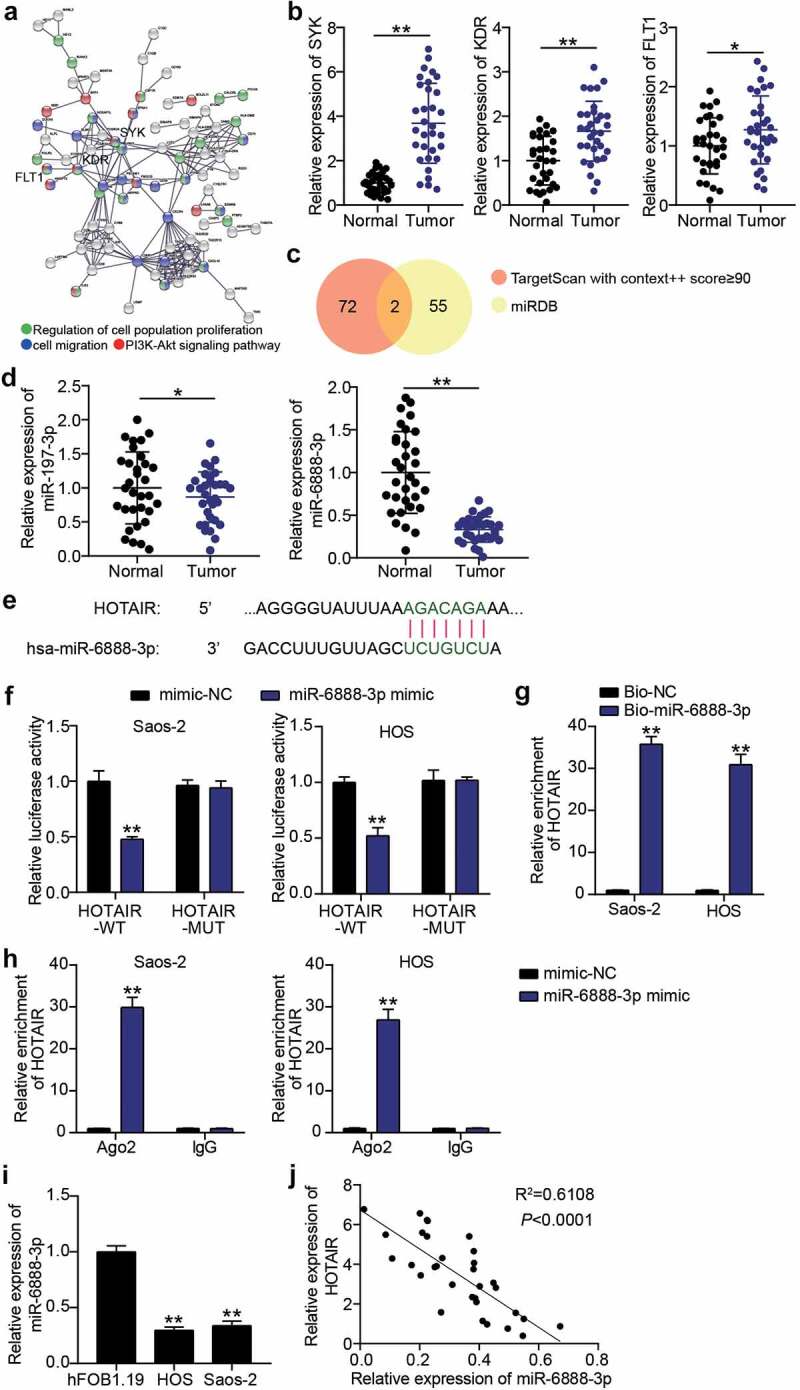
miR-6888-3p was a target for HOTAIR (a) SYK, KDR, and FLT1 were associated with cell migration, cell proliferation and PI3K-Akt signaling pathway by STRING analysis. (b) qRT-PCR for relative SYK, KDR, FLT1 expression in OS tissues and adjacent tissues. * P < 0.05, ** P < 0.001. (c) miR-197-3p and miR-6888-3p were predicted to bind to SYK and HOTAIR by TargetScan and miRDB. (d) qRT-PCR for relative miR-197-3p and miR-6888-3p expression in OS tissues and adjacent tissues. ** P < 0.001. (e) Bioinformatics analysis for predicting miR-6888-3p binding sites in HOTAIR. (f) Luciferase reporter assays using HOS and Saos2 cells co-transfected with the miR-6888-3p mimic and HOTAIR-WT or HOTAIR-MUT reporte*r plasmid*. ** P < 0.001. vs mimic-NC. (g) RNA pull-down assay was performed to measure HOTAIR levels in HOS and Saos2 cells transfected with Bio-miR-6888-3p. ** P < 0.001. vs anti-IgG. (h) RIP assay was performed to measure miR-6888-3p and HOTAIR levels on anti-IgG and anti-Ago2. ** P < 0.001. vs anti-IgG. (i) qRT-PCR for the determination of miR-6888-3p expression in OS cell lines. ** P < 0.001. vs hFOB1.19. (j) Negative association between HOTAIR and miR-6888-3p expression in OS tissues.

Subsequently, we mined the miRNAs downstream of HOTAIR using bioinformatics. GSE12865, including OS and non-tumor samples, was used to screen for upregulated genes. With an adjusted *p*-value < 0.01 and log FC > 2, a total of 291 upregulated genes in OS samples were screened out (Supplementary Table S1). STRING implemented GO and KEGG enrichment analyses of the genes screened in the OS samples and established an association between SYK, KDR, FLT1, and cell proliferation, migration, and the PI3K-AKT signaling pathway.

(Figure 3(a)). The expression of SYK, KDR, and FLT1 was detected using qRT-PCR to screen for the mRNA of interest. According to our results, SYK, KDR, and FLT1 levels in cancer tissues were 3.7 times, 1.7 times and 1.3 times higher than those in normal tissues, respectively. SYK was identified as the mRNA of interest (Figure 3(b)). To identify the key miRNAs connecting SYK and HOTAIR, TargetScan and miRDB were used to predict miRNA binding to SYK and HOTAIR. TargetScan and miRDB results showed that miR-197-3p and miR-6888-3p were common miRNAs (Figure 3(c)). Subsequently, the expression levels of miR-197-3p and miR-6888-3p detected in cancer tissues showed that there was no significant difference in the expression of miR-197-3p, and that miR-6888-3p was significantly downregulated. Therefore, miR-6888-3p was selected for subsequent experiments (Figure 3(d)). After comparing the information, HOTAIR sequences were found to contain sites binding to miR-6888-3p (Figure 3(e)). Luciferase analysis revealed that luciferase activity in wild-type HOTAIR vector-transfected HOS and Saos2 cells decreased by more than 50% in the miR-6888-3p mimic group compared to that in the mimic-NC group (figure 3(f)). An RNA pull-down assay was performed with Bio-miR-6888-3p to test the ability of HOTAIR to act as an miRNA sponge. The results showed that the expression level of HOTAIR was higher in the Bio-miR-6888-3p group than in the Bio-NC group (Figure 3(g)). Furthermore, RIP results suggested that miR-6888-3p and HOTAIR levels were significantly enriched in anti-Ago2 (Figure 3(h)). Additionally, qRT-PCR analysis showed that the miR-6888-3p levels in HOS and Saos2 cells were lower than those in hFOB1.19 cells (Figure 3(i)). HOTAIR and miR-6888-3p expression levels were found to be negatively correlated in the OS tissue (Figure 3(j)). That is, miR-6888-3p expression was downregulated in OS and inhibited by the HOTAIR sponge.

### HOTAIR regulated the PI3K/AKT pathway by influencing the proliferation and migration of OS cells via miR-6888-3p sponging

**Figure 4.**
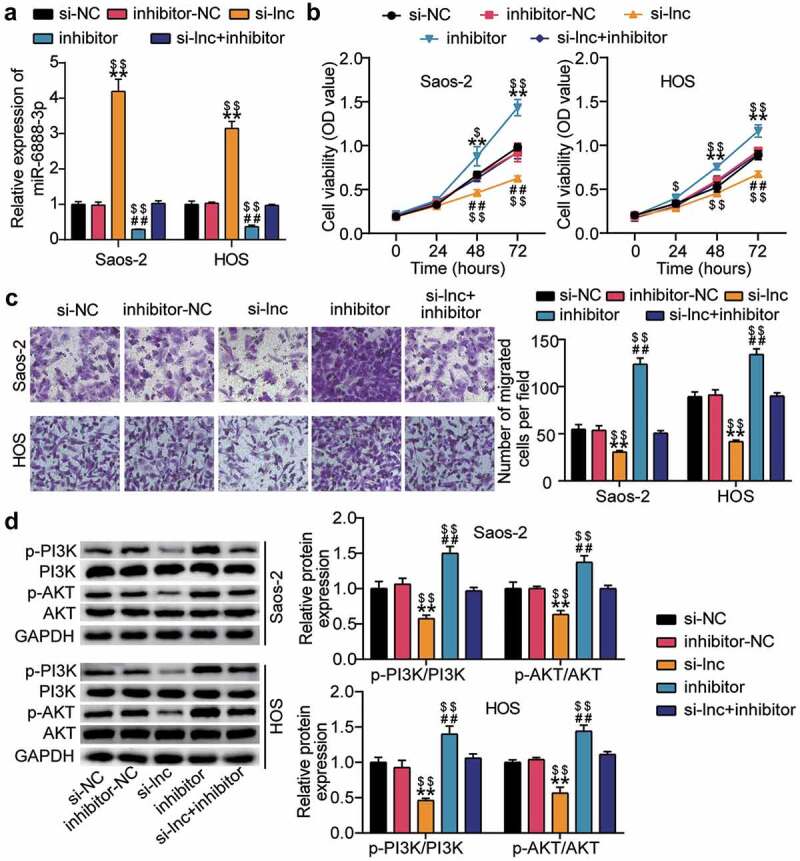
HOTAIR affected the proliferation and migration of OS cells through sponge miR-6888-3p, and regulated the PI3K/AKT pathway (a) qRT-PCR for miR-6888-3p expression in si-lnc transfected or miR-6888-3p inhibitor transfected cells. (b) Cell viability was detected by CCK-8 assay in HOS and Saos2 cells transfected with si-lnc or miR-6888-3p inhibitor. (c) Migrated cells were counted by transwell assay in HOS and Saos2 cells transfected with si-lnc or miR-6888-3p inhibitor. (d) The expression of PI3K, AKT, p-PI3K and p-AKT protein levels was detected by Western blot analysis in HOS and Saos2 cells transfected with si-lnc or miR-6888-3p inhibitor. * P < 0.05, ** P < 0.001. vs si-NC treatment. # P < 0.05, ## P < 0.001. vs inhibitor-NC treatment. $ P < 0.05, $$ P < 0.001. vs si-lnc+inhibitor treatment.

HOTAIR and miR-6888-3p were co-silenced in HOS and Saos2 cells to further investigate the effect of HOTAIR/miR-6888-3p on the proliferation, migration, and PI3K/AKT pathways of OS cells. According to the qRT-PCR analysis, knockdown of HOTAIR increased the miR-6888-3p levels to more than three-folds and, knockdown of miR-6888-3p decreased the miR-6888-3p levels by approximately 80% and eliminated the effect of HOTAIR knockdown (Figure 4(a)). Simultaneously, CCK-8 and transwell migration experiments showed that downregulating miR-6888-3p significantly promoted cell proliferation and migration while reversing the inhibitory effects of silencing HOTAIR relatively (Figure 4(b,c)). Additionally, detection of PI3K/AKT pathway-related protein levels revealed that the expression of p-PI3K and p-AKT proteins increased after miR-6888-3p inhibition, subsequently, the PI3K/AKT pathway was reactivated after silent treatment (Figure 4(d)). These results indicate that the miR-6888-3p inhibitor promoted the proliferation and migration of cancer cells and activated the PI3K/AKT pathway to reverse the effect of HOTAIR knockdown on HOS and Saos2 cells.

### miR-6888-3p negatively regulates SYK expression

**Figure 5.**
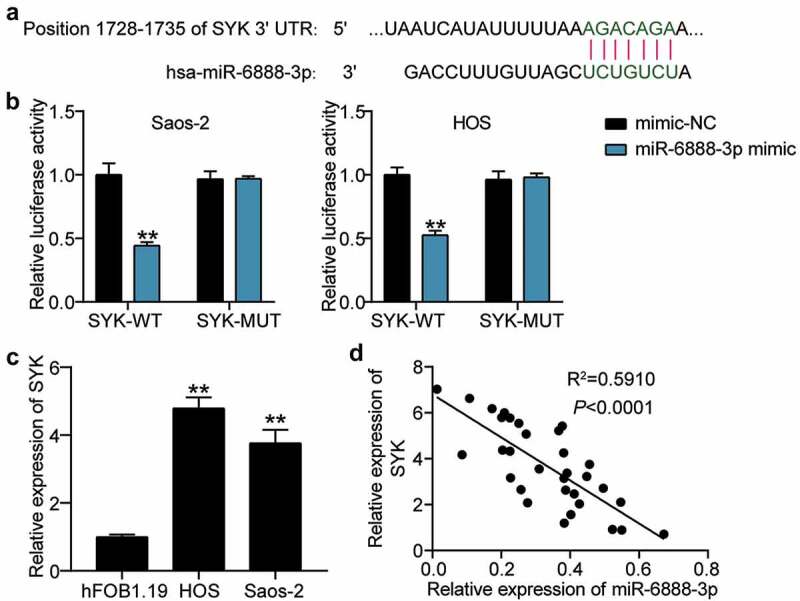
miR-6888-3p negatively targeted SYK (a) Bioinformatics analysis for predicting SYK binding sites in miR-6888-3p. (b) Luciferase reporter assays using HOS and Saos2 cells co-transfected with the miR-6888-3p mimic and SYK-WT or SYK-MUT reporte*r plasmid*. (c) qRT-PCR for the determination of SYK expression in OS cell lines. ** P < 0.001. vs hFOB1.19. (d) Negative association between SYK and miR-6888-3p expression in OS tissues.

Having retrieved SYK from the database, we then focused on demonstrating the relationship between miR-6888-3p and SYK. The TargetScan search revealed that the SYK 3'UTR contained miR-6888-3p binding targets (Figure 5(a)). Luciferase analysis revealed that the miR-6888-3p mimic significantly inhibited the luciferase activity of SYK-WT; however, it had no significant effect on the luciferase activity of SYK-MUT, suggesting that miR-6888-3p targeted SYK (Figure 5(b)). In addition, qRT-PCR showed that SYK levels in HOS and Saos2 cells were 5-fold and 3.8-fold, respectively, compared to those in HFOB1.19 cells (Figure 5(c)). Moreover, at the tissue level, miR-6888-3p expression negatively correlated with SYK expression in OS tissues (Figure 5(d)). In other words, miR-6888-3p targets and negatively regulates SYK expression.

### miR-6888-3p inhibits the levels of SYK, thereby suppressing the proliferation and migration of OS cells and the PI3K/AKT pathway

**Figure 6.**
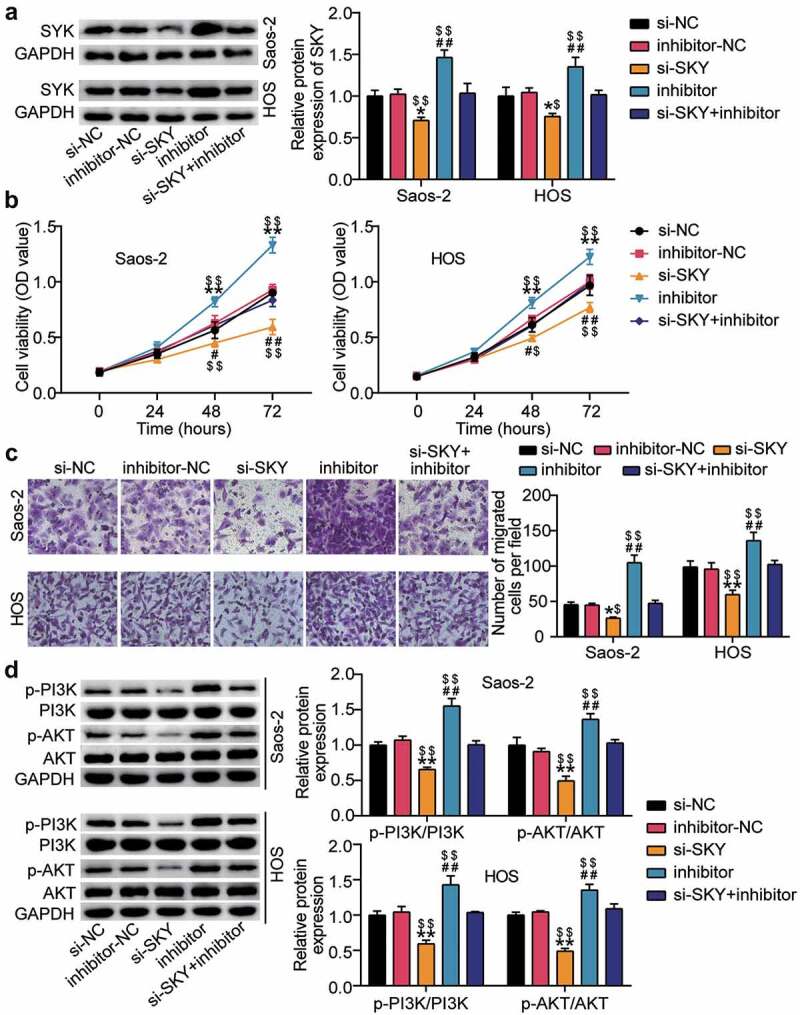
miR-6888-3p suppressed the proliferation and migration of OS cells and the PI3K/AKT pathway by inhibiting the level of SYK (a) Western blot assay for SYK protein expression in si-SYK transfected or miR-6888-3p inhibitor transfected cells. (b) Cell viability was detected by CCK-8 assay in HOS and Saos2 cells transfected with si-SYK or miR-6888-3p inhibitor. (c) Migrated cells were counted by transwell assay in HOS and Saos2 cells transfected with si-SYK or miR-6888-3p inhibitor. (d) The expression of PI3K, AKT, p-PI3K and p-AKT protein levels was detected by Western blot analysis in HOS and Saos2 cells transfected with si-SYK or miR-6888-3p inhibitor. * P < 0.05, ** P < 0.001. vs si-NC treatment. # P < 0.05, ## P < 0.001. vs inhibitor-NC treatment. $ P < 0.05, $$ P < 0.001. vs si-SKY+inhibitor treatment.

Western blot analysis showed that SYK protein levels increased after miR-6888-3p inhibition and decreased after si-SYK treatment (Figure 6(a)). Functional analysis showed that SYK knockdown decreased cell viability beyond 30%, reduced cell migration beyond 35%, and eliminated the pro-cell viability and migration effects of the miR-6888-3p inhibitor (Figure 6(b,c)). Furthermore, we explored the effects of miR-6888-3p and SYK on the PI3K/AKT pathway and uncovered that SYK silencing downregulated both p-PI3K and p-AKT protein levels, reversing the effects of interfering with miR-6888-3p (Figure 6(d)).

## Discussion

Increasing evidence suggests that abnormal lncRNA expression in cancer is closely linked to tumorigenesis, metastasis, and prognosis [28]. Previous studies established that HOTAIR was an oncogenic factor for OS, promoting *in vitro* proliferation, metastasis, cisplatin resistance, inhibition of apoptosis, and cell cycle arrest in cancer cells [29,30]. In this study, we measured HOTAIR expression in OS samples and their corresponding non-tumor tissues. Consistent with previous studies, HOTAIR was found to be upregulated in OS tissues. Additionally, the function of HOTAIR was identified by loss of function in OS cells. Based on the confirmation that HOTAIR deletion inhibited the proliferation and migration of OS cells *in vitro*, it was further revealed that HOTAIR knockdown inhibited tumor growth *in vivo*. These results suggest that HOTAIR acts as an oncogene that promotes malignant progression of OS and may be a diagnostic and prognostic marker for the disease.

HOTAIR expression participates in an automatic regulatory circuit, suppressing miR-217 expression and activity, and resulting in upregulation of ZEB1 expression in OS [9]. This study demonstrates the existence of a similar regulatory network for HOTAIR. miR-6888-3p was selected as a downstream target of HOTAIR through bioinformatic studies. As expected, the luciferase assay confirmed the direct binding of the miR-6888-3p reaction element to full-length HOTAIR. In addition, the expression of miR-6888-3p in OS negatively correlated with HOTAIR, and analysis at the cellular level showed that miR-6888-3p was negatively regulated by HOTAIR. Further studies revealed that interference with miR-6888-3p promotes the proliferation and migration of cancer cells and reverses the effect of HOTAIR knockdown on cell function. Consequently, HOTAIR may act as an endogenous sponge to inhibit the expression and function of miR-6888-3p.

Increasing evidence suggests that SYK is essential for tumor cell proliferation, metabolism, and metastasis [17,31]. This study focused on SYK for further investigation and observed a negative correlation between SYK mRNA and miR-6888-3p expression in OS tissues. Subsequently, it was confirmed that SYK is a direct target of miR-6888-3p and can eliminate interference with miR-6888-3p induced OS cell proliferation and migration. Therefore, the influence of HOTAIR/miR-6888-3p/SYK may have biological significance in the regulatory network of tumorigenesis in OS.

Additionally, western blot analysis revealed that HOTAIR/miR-6888-3p/SYK could affect the progression of OS through the PI3K/AKT signaling pathway. In this study, we elucidated the blocking effect of HOTAIR or SYK knockdown on PI3K/AKT pathway activation and the inducing effect of interference with miR-6888-3p. These findings provide a new signaling pathway regulatory network for OS.

However, this study has some limitations. First, the clinical relevance of HOTAIR/miR-6888-3p/SYK in the prognosis, metastasis, and recurrence of OS patients, needs to be further investigated. Additionally, the correlation between HOTAIR expression and clinicopathological features along with the survival outcomes need to be explored. Therefore, this should be the focus of future research.

## Conclusion

In conclusion, our current work highlights that the novel HOTAIR/miR-6888-3p/SYK signaling axis promotes the malignant progression of OS by activating the PI3K/AKT pathway. This provides a cue for understanding the pathogenesis of OS and is an interesting approach for the diagnosis and treatment of OS.

## Supplementary Material

Supplemental Material

## Data Availability

The datasets used and/or analyzed during the current study are available from the corresponding author on reasonable request.
