# Supplementary figures and images for: Hox transcript antisense RNA knockdown inhibits osteosarcoma progression by regulating the phosphoinositide 3-kinase/AKT pathway through the microRNA miR-6888-3p/spleen tyrosine kinase axis
Source: Bioengineered. 2022 Apr 17;13(4):9397–410. doi: 10.1080/21655979.2022.2059614 (PMC9161905; doi:10.1080/21655979.2022.2059614)

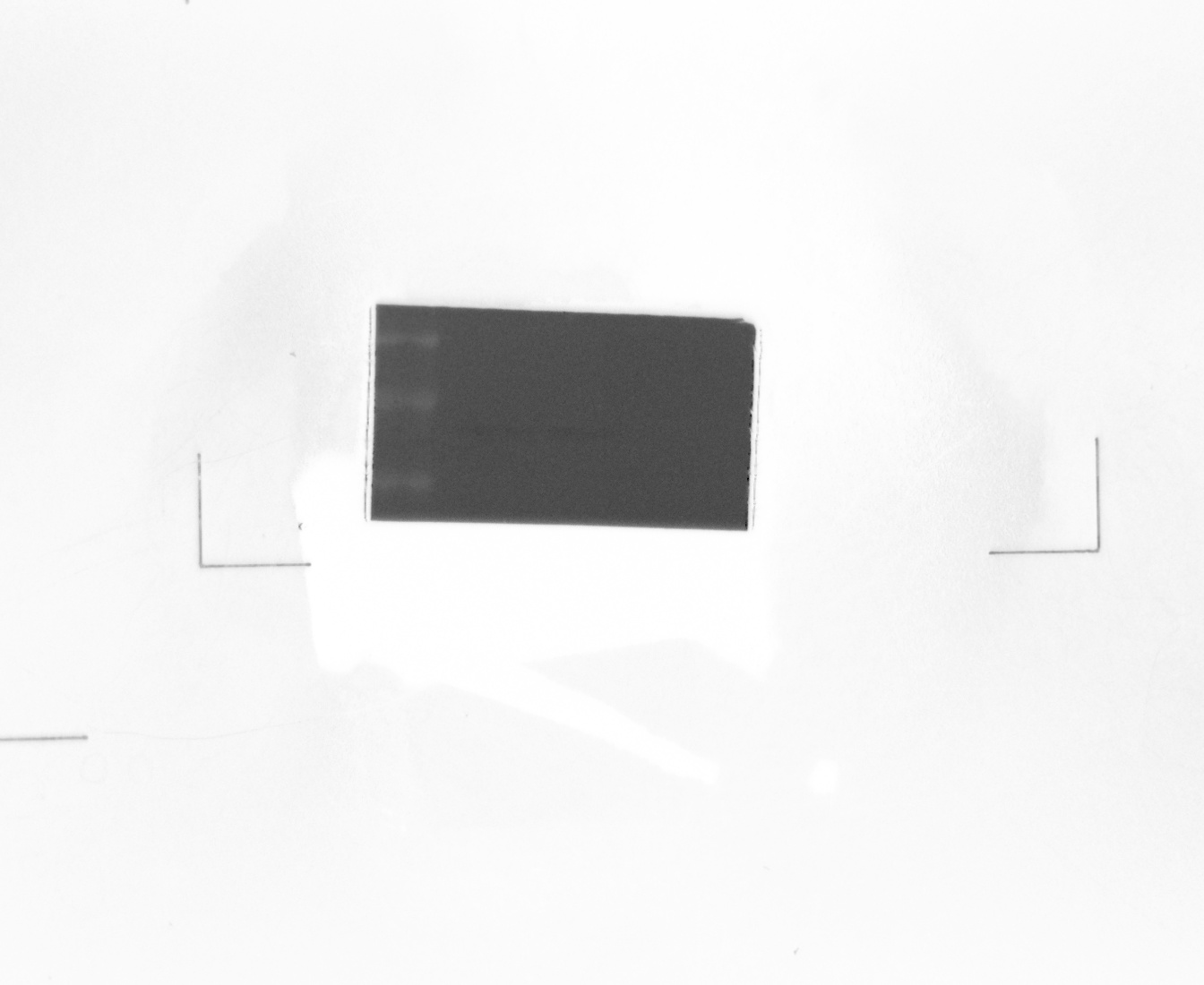

Supplement: Supplemental Material [file KBIE_A_2059614_SM8919.zip › Supplementary Material/Figure 1H/HOS AKT-bright field.jpg]

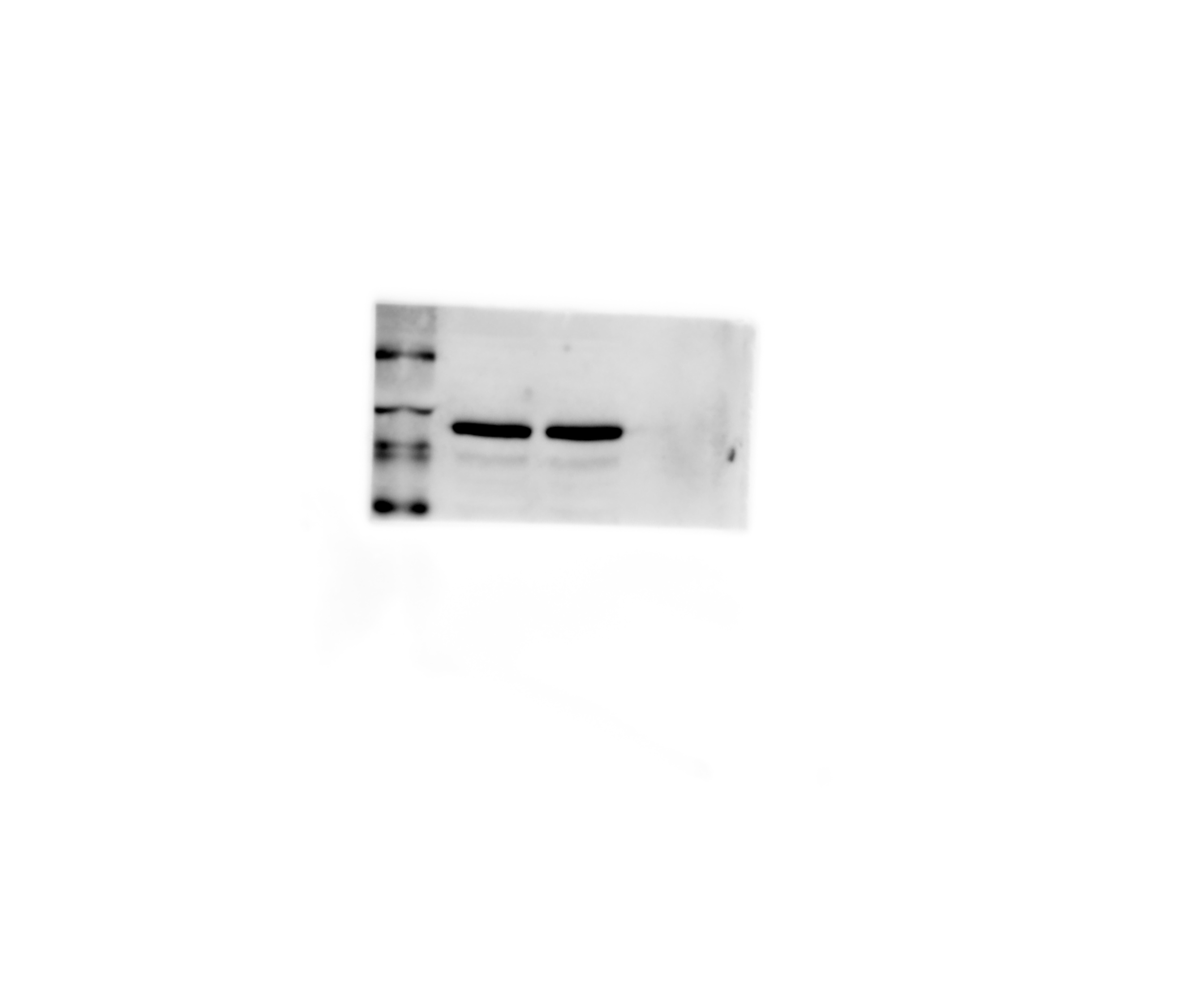

Supplement: Supplemental Material [file KBIE_A_2059614_SM8919.zip › Supplementary Material/Figure 1H/HOS AKT.jpg]

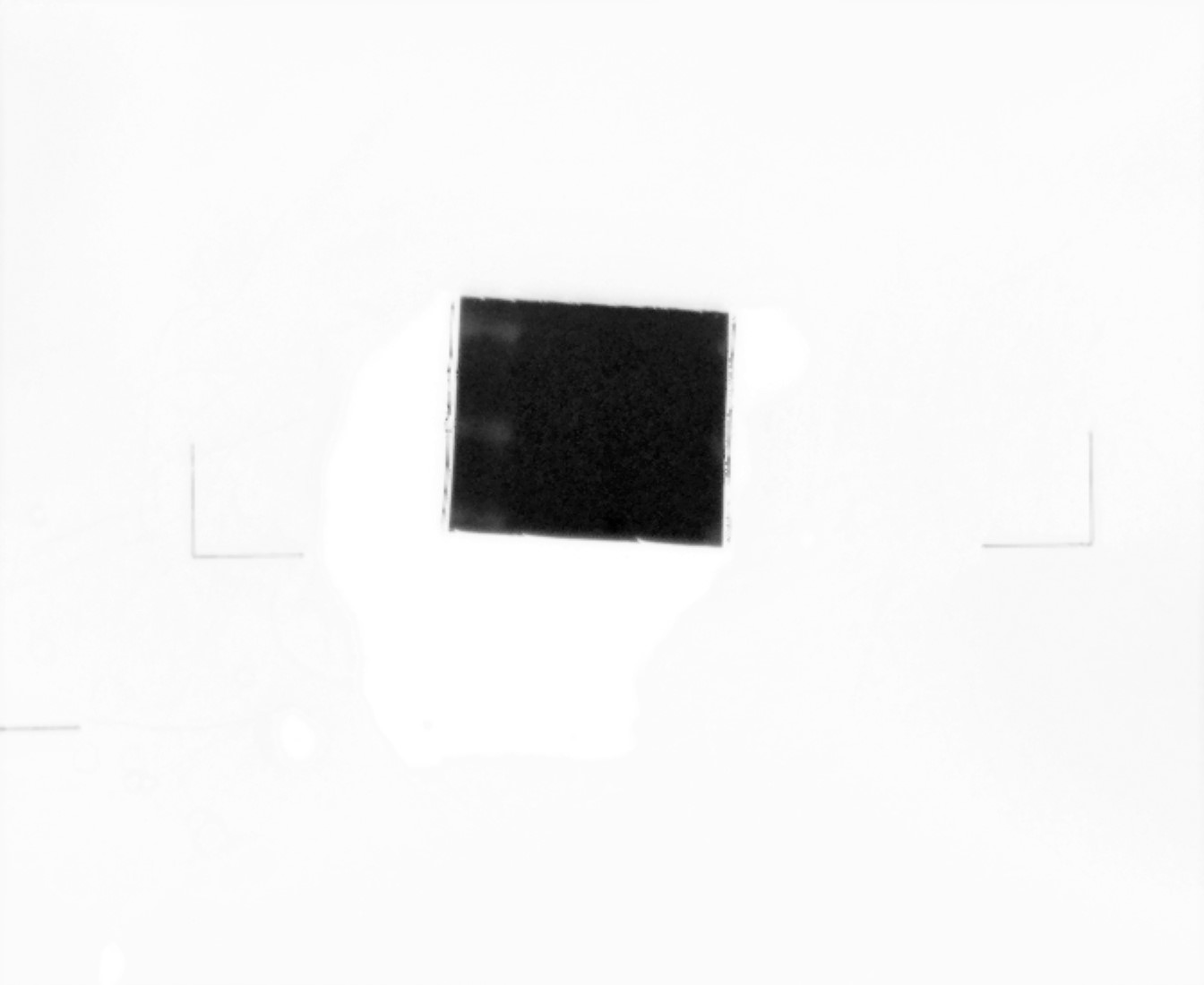

Supplement: Supplemental Material [file KBIE_A_2059614_SM8919.zip › Supplementary Material/Figure 1H/HOS GAPDH-bright field.jpg]

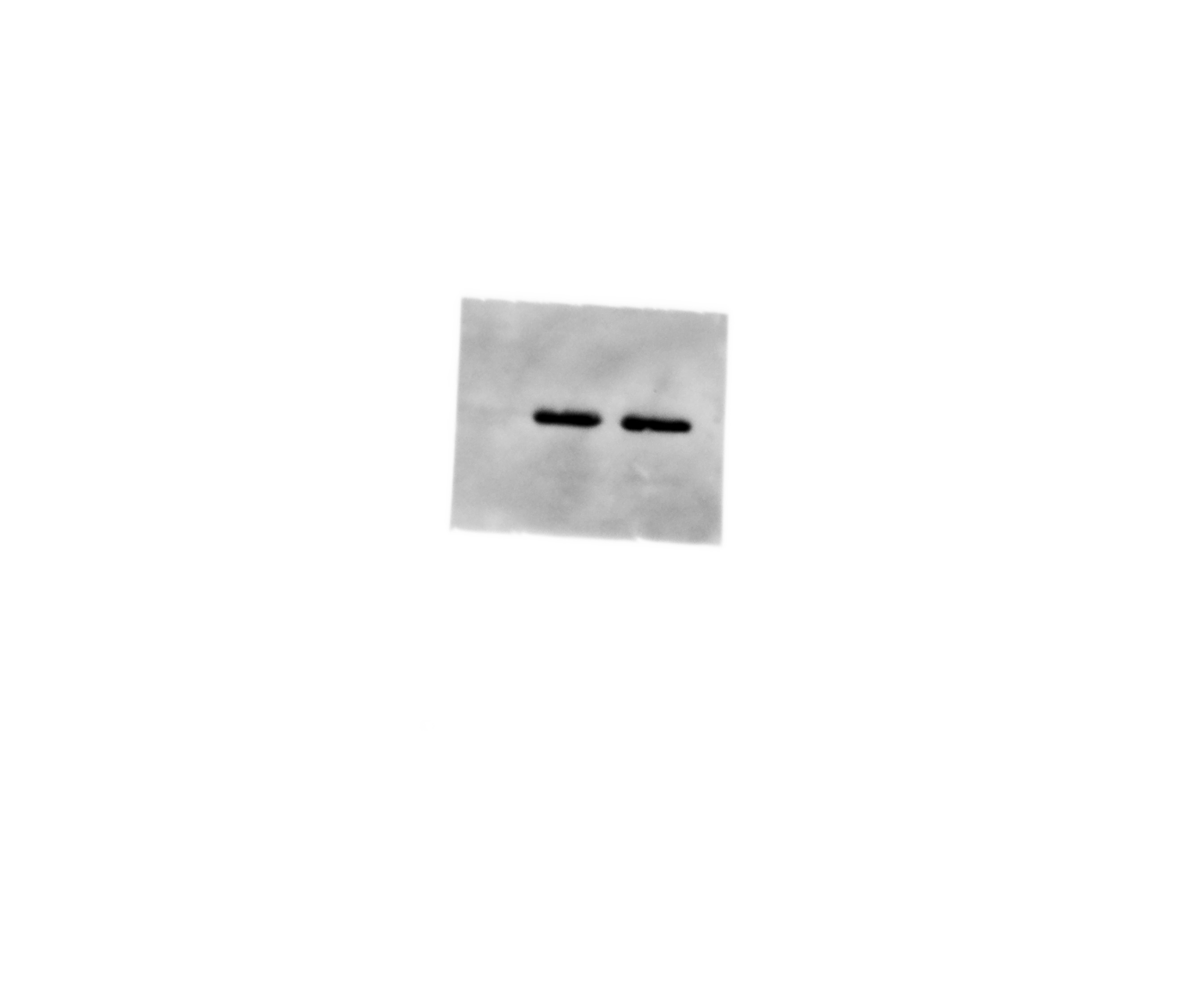

Supplement: Supplemental Material [file KBIE_A_2059614_SM8919.zip › Supplementary Material/Figure 1H/HOS GAPDH.jpg]

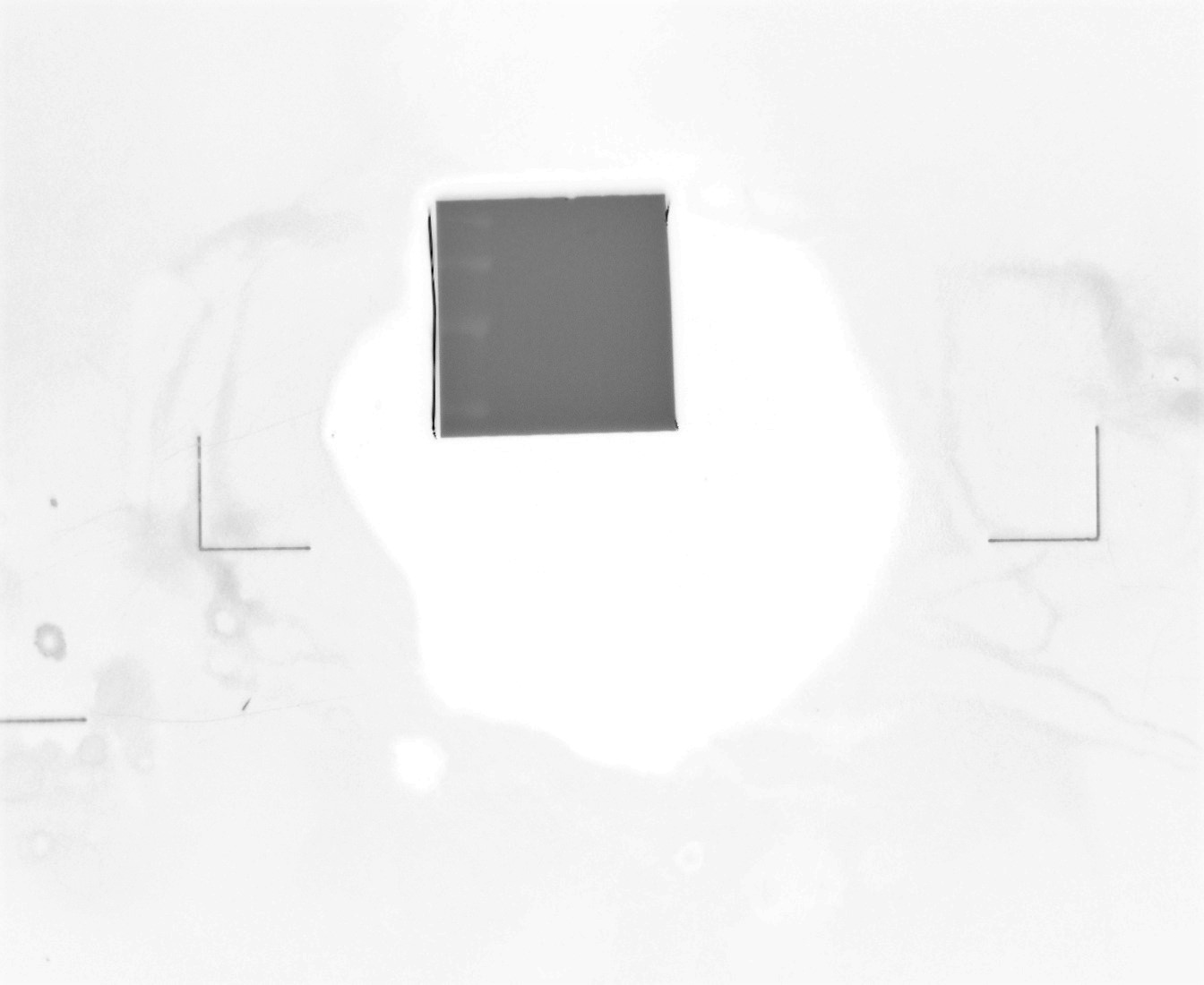

Supplement: Supplemental Material [file KBIE_A_2059614_SM8919.zip › Supplementary Material/Figure 1H/HOS P-AKT-bright field.jpg]

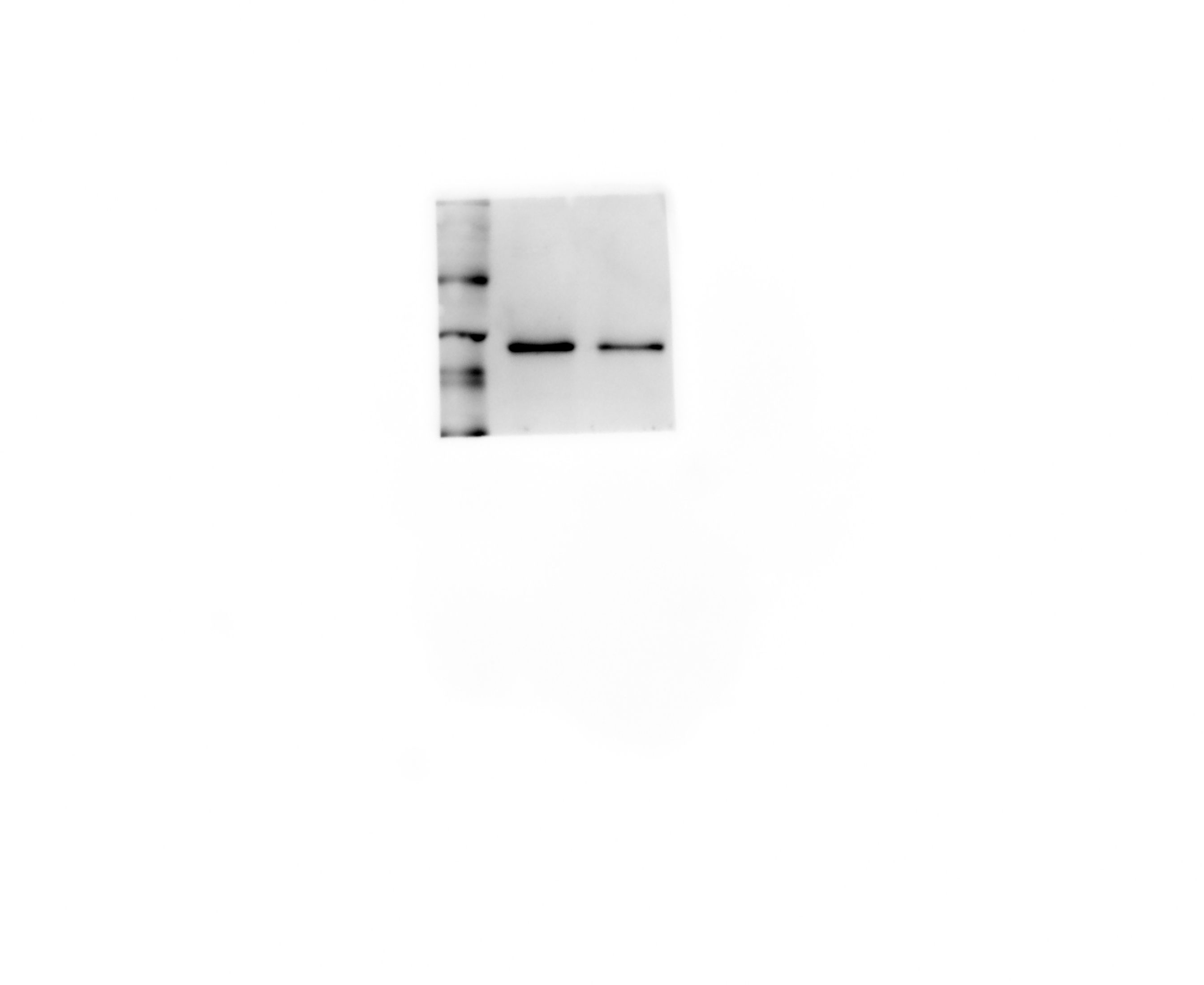

Supplement: Supplemental Material [file KBIE_A_2059614_SM8919.zip › Supplementary Material/Figure 1H/HOS P-AKT.jpg]

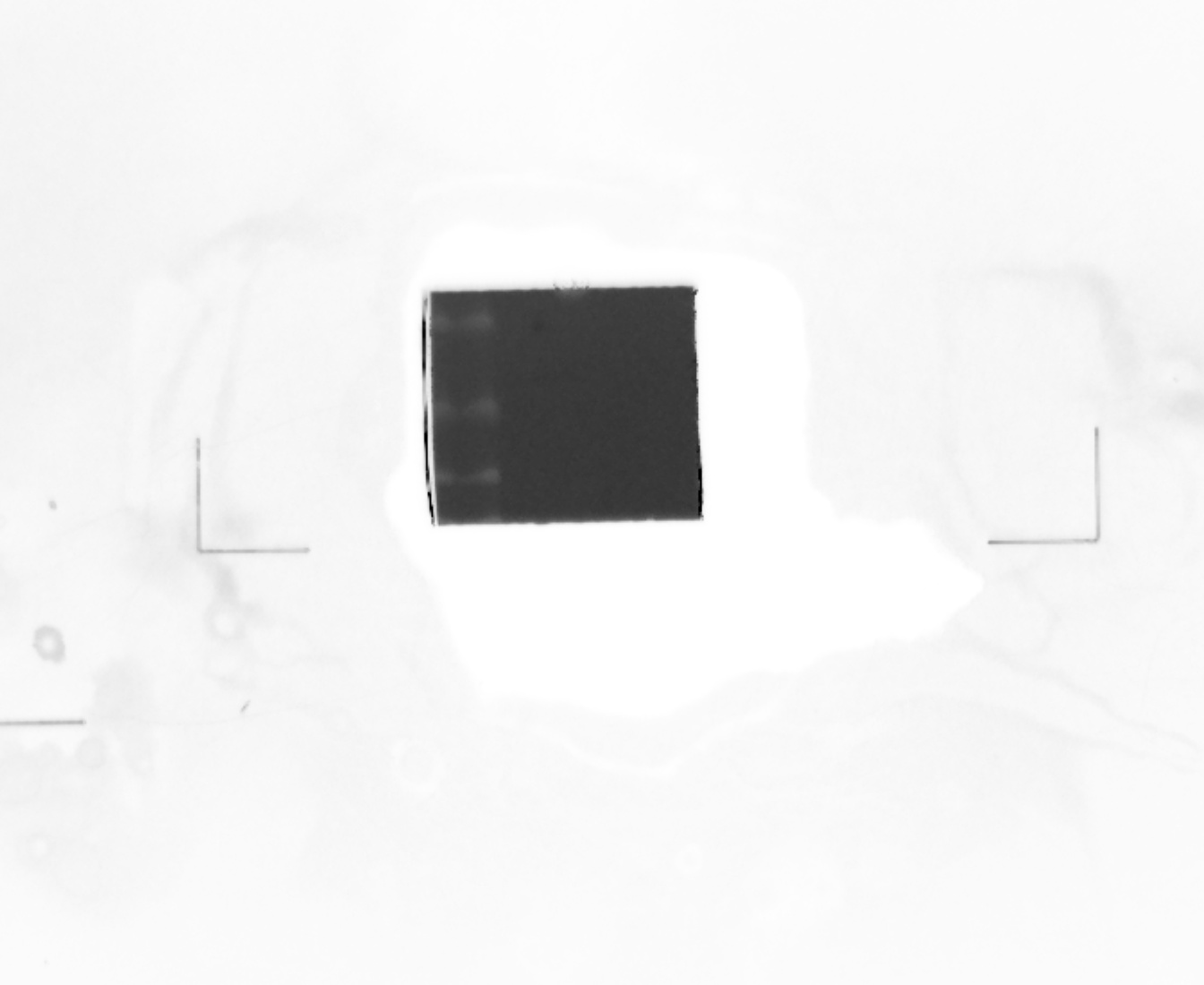

Supplement: Supplemental Material [file KBIE_A_2059614_SM8919.zip › Supplementary Material/Figure 1H/HOS P-PI3K-bright field.jpg]

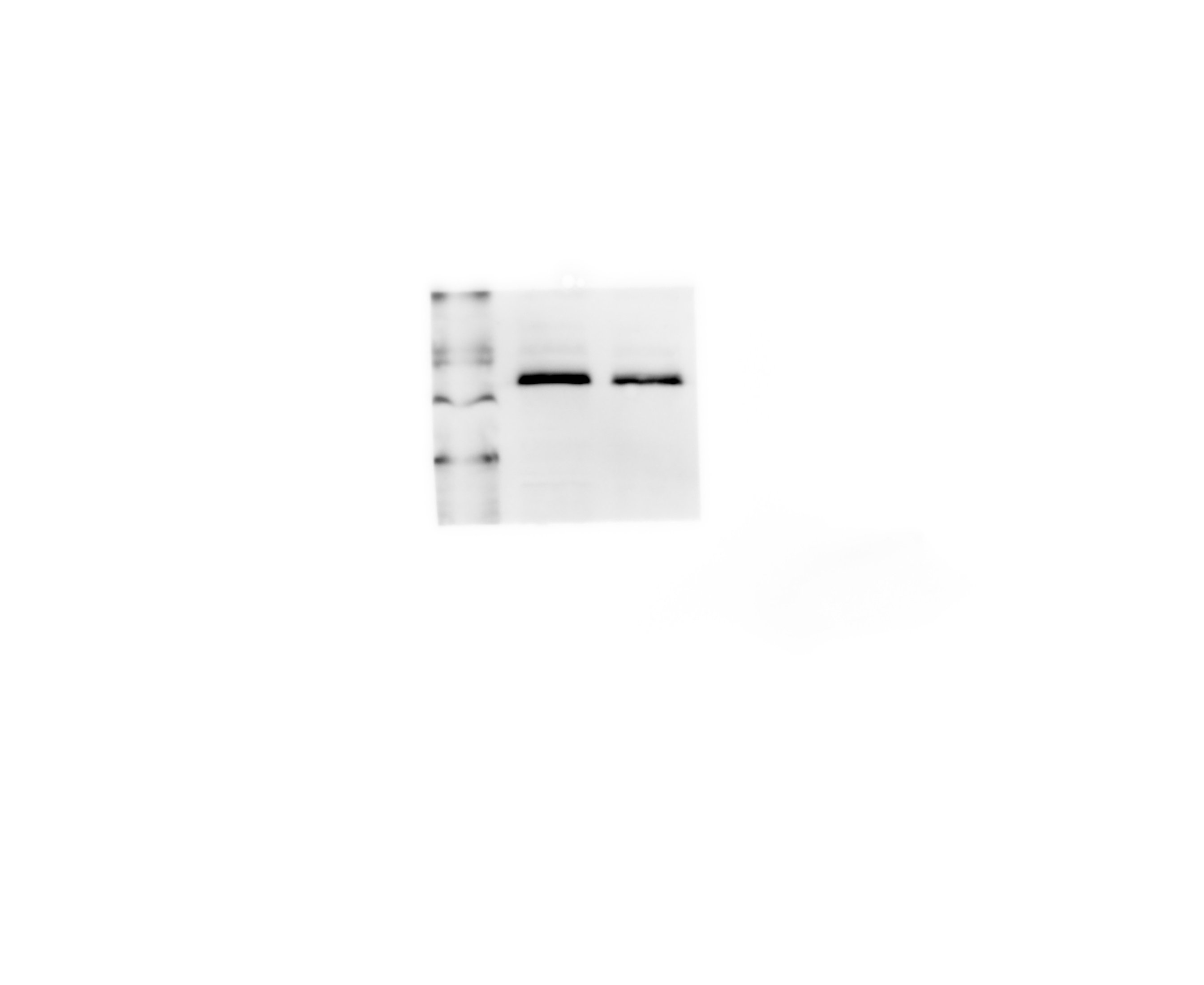

Supplement: Supplemental Material [file KBIE_A_2059614_SM8919.zip › Supplementary Material/Figure 1H/HOS P-PI3K.jpg]

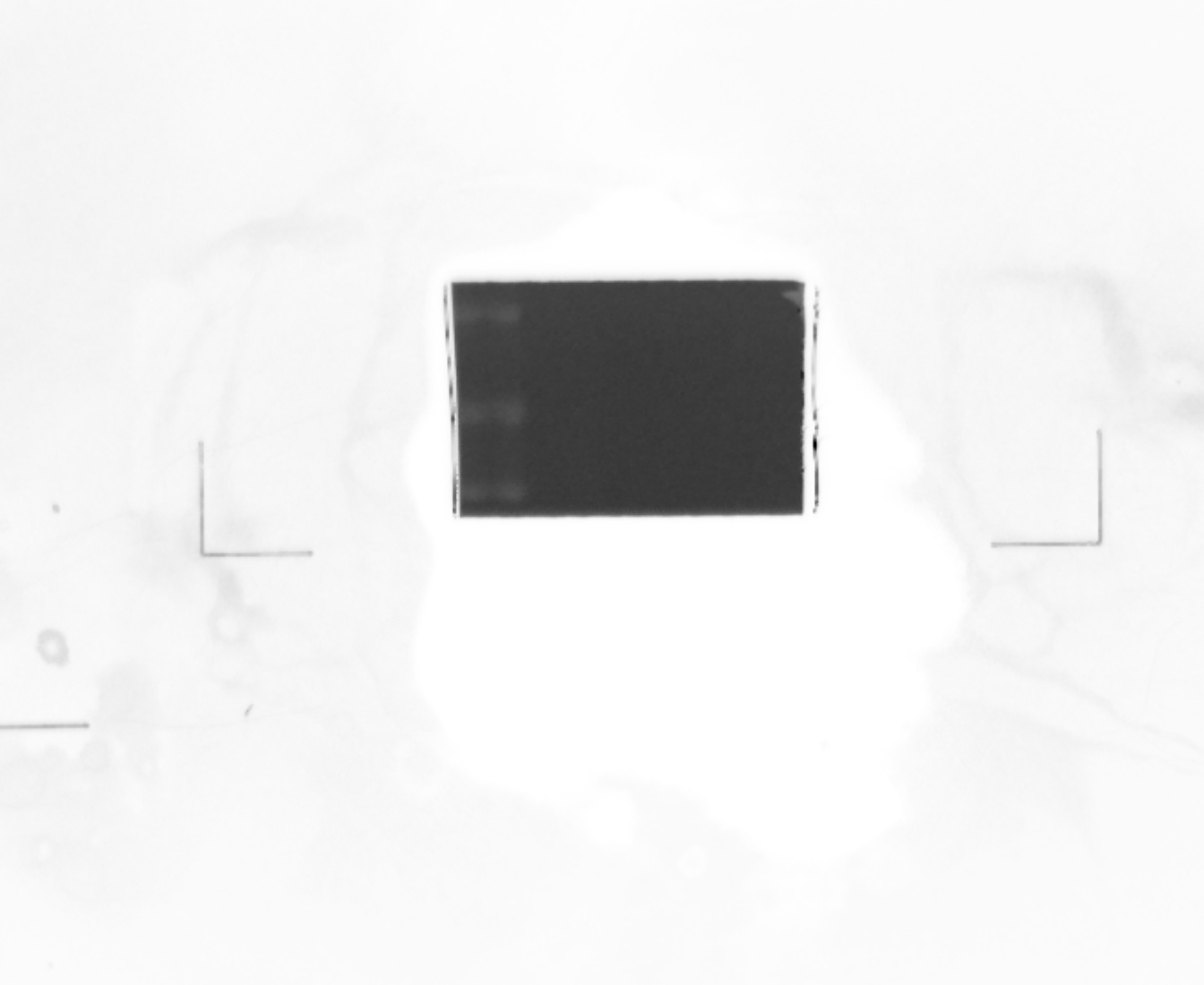

Supplement: Supplemental Material [file KBIE_A_2059614_SM8919.zip › Supplementary Material/Figure 1H/HOS PI3K-bright field.jpg]

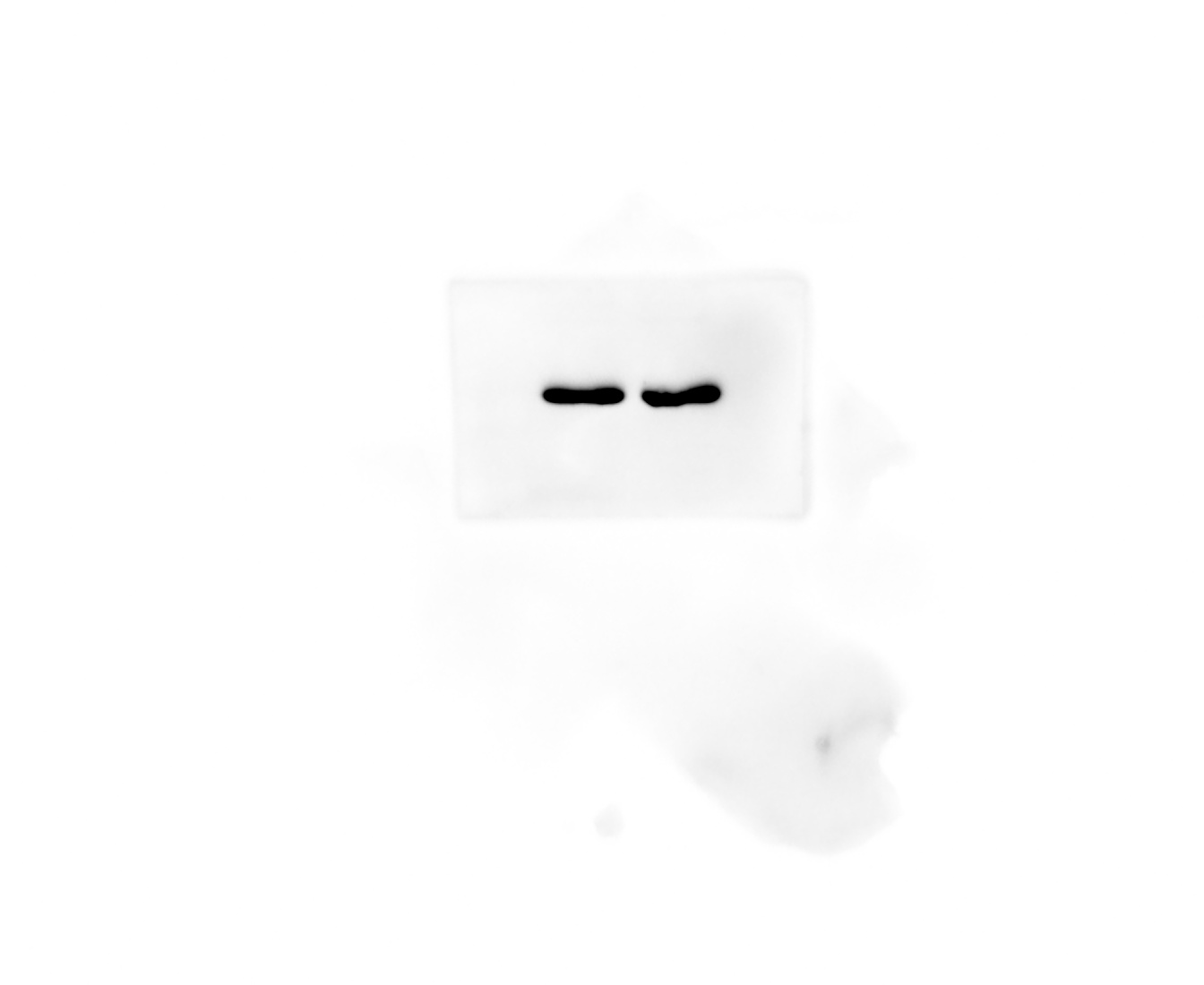

Supplement: Supplemental Material [file KBIE_A_2059614_SM8919.zip › Supplementary Material/Figure 1H/HOS PI3K.jpg]

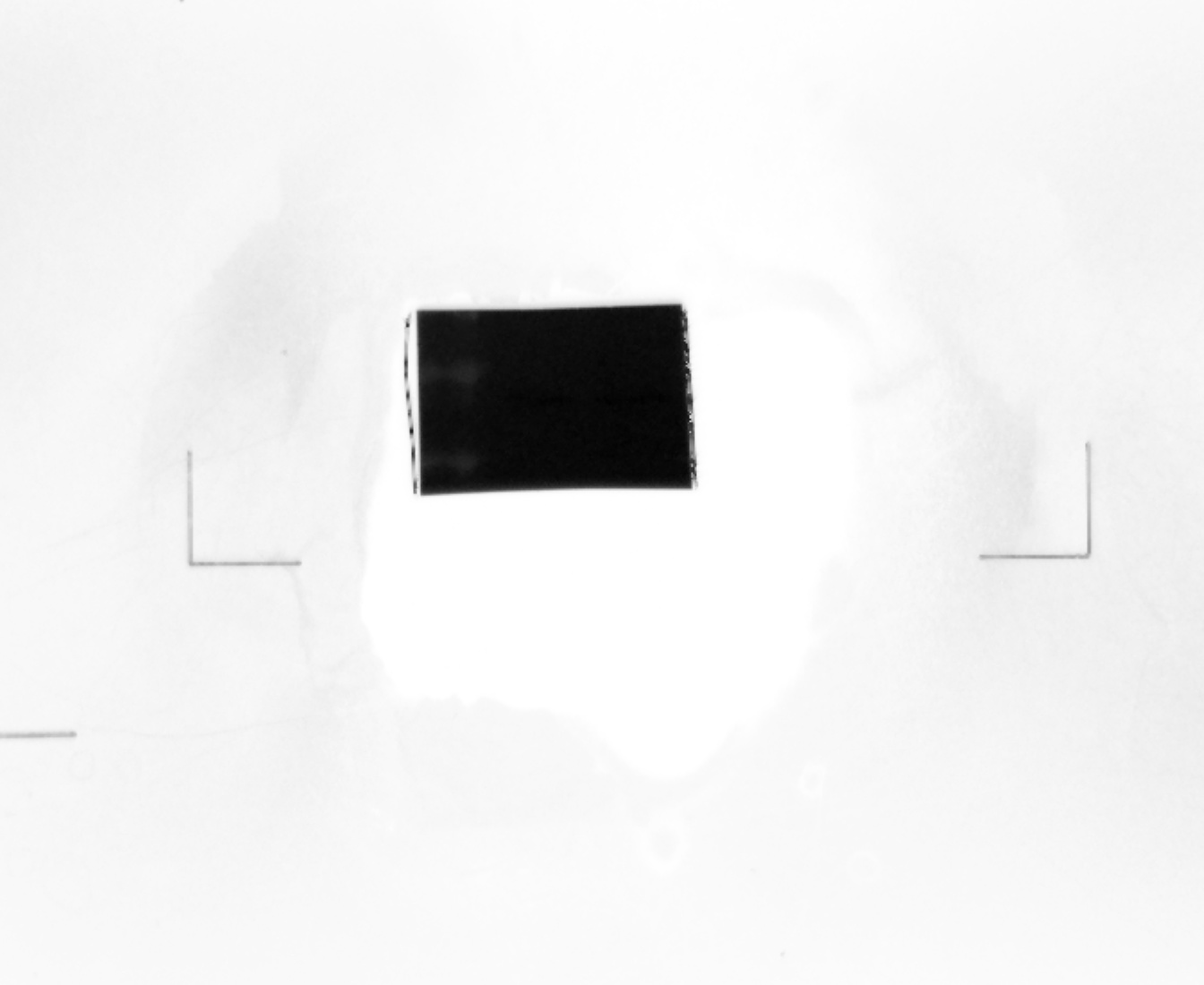

Supplement: Supplemental Material [file KBIE_A_2059614_SM8919.zip › Supplementary Material/Figure 1H/Saos-2 AKT-bright field.jpg]

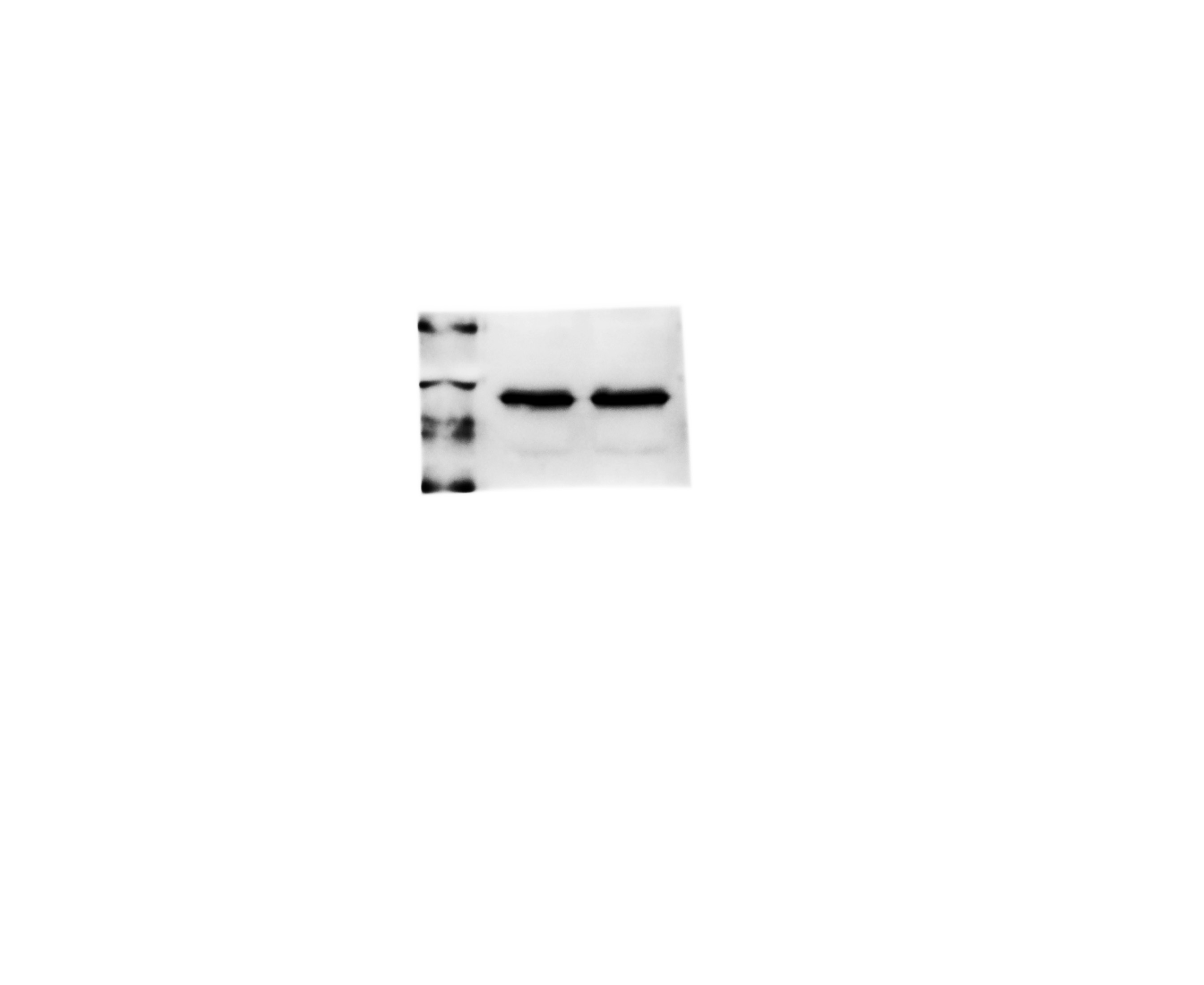

Supplement: Supplemental Material [file KBIE_A_2059614_SM8919.zip › Supplementary Material/Figure 1H/Saos-2 AKT.jpg]

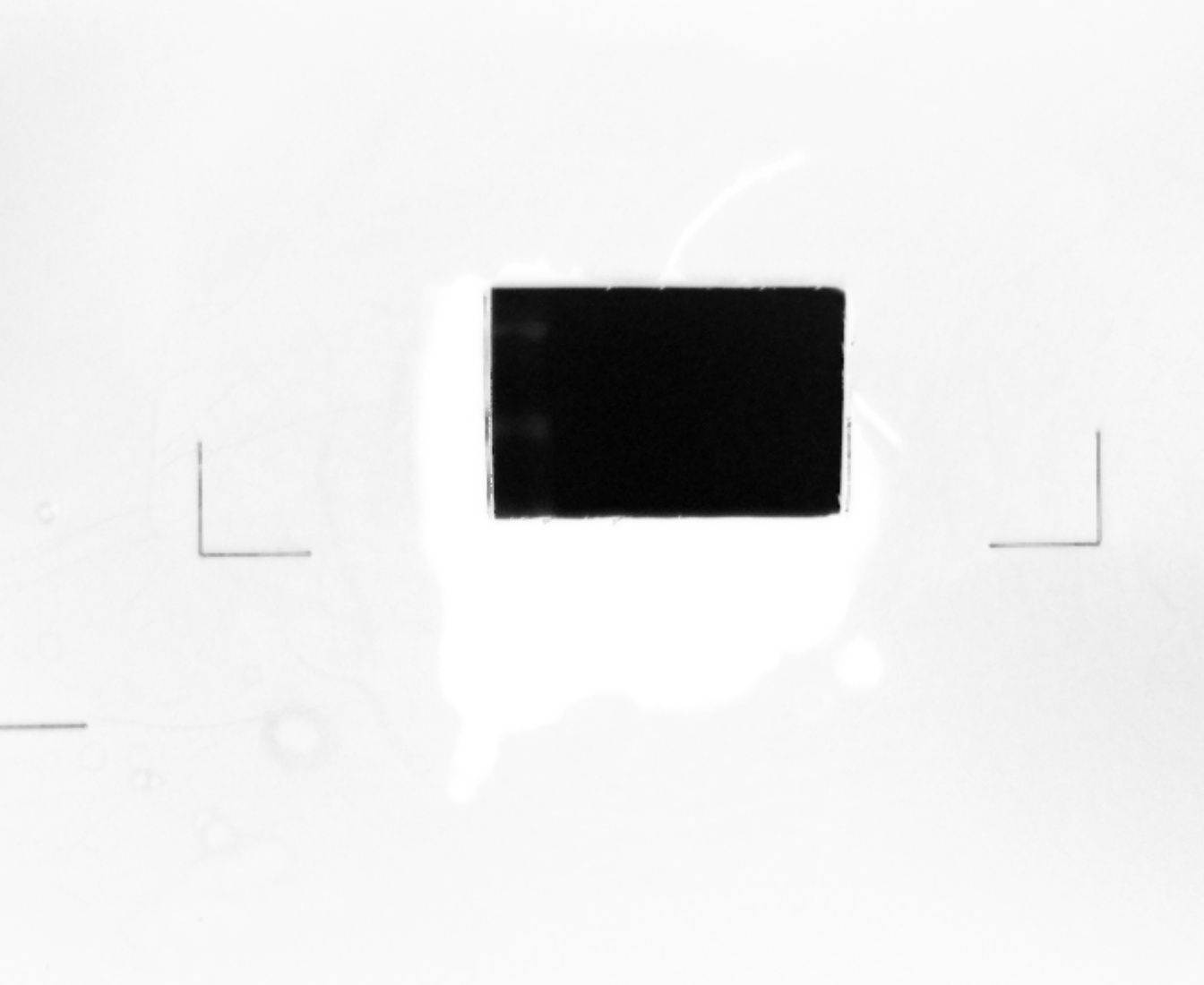

Supplement: Supplemental Material [file KBIE_A_2059614_SM8919.zip › Supplementary Material/Figure 1H/Saos-2 GAPDH-bright field.jpg]

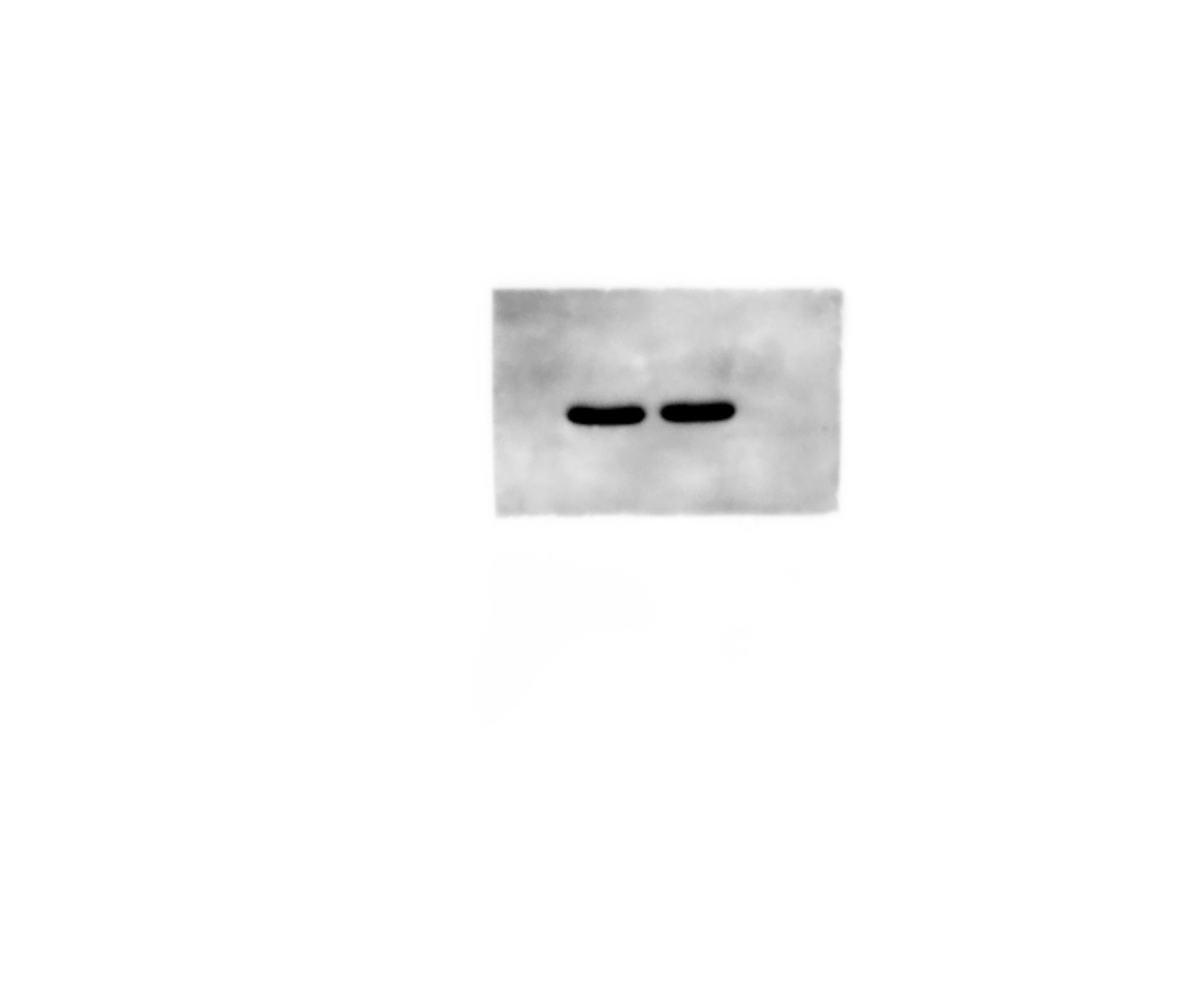

Supplement: Supplemental Material [file KBIE_A_2059614_SM8919.zip › Supplementary Material/Figure 1H/Saos-2 GAPDH.jpg]

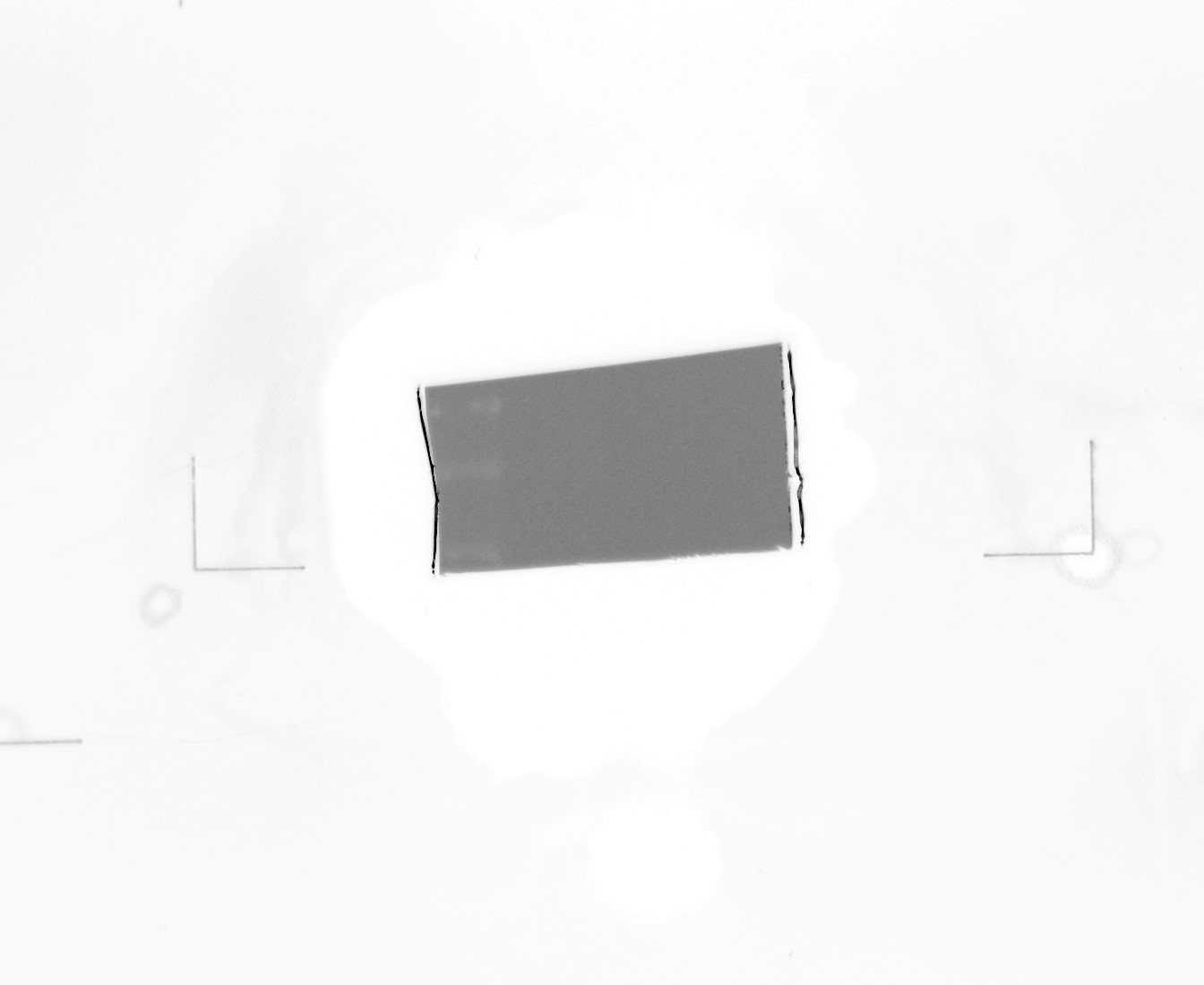

Supplement: Supplemental Material [file KBIE_A_2059614_SM8919.zip › Supplementary Material/Figure 1H/Saos-2 P-AKT-bright field.jpg]

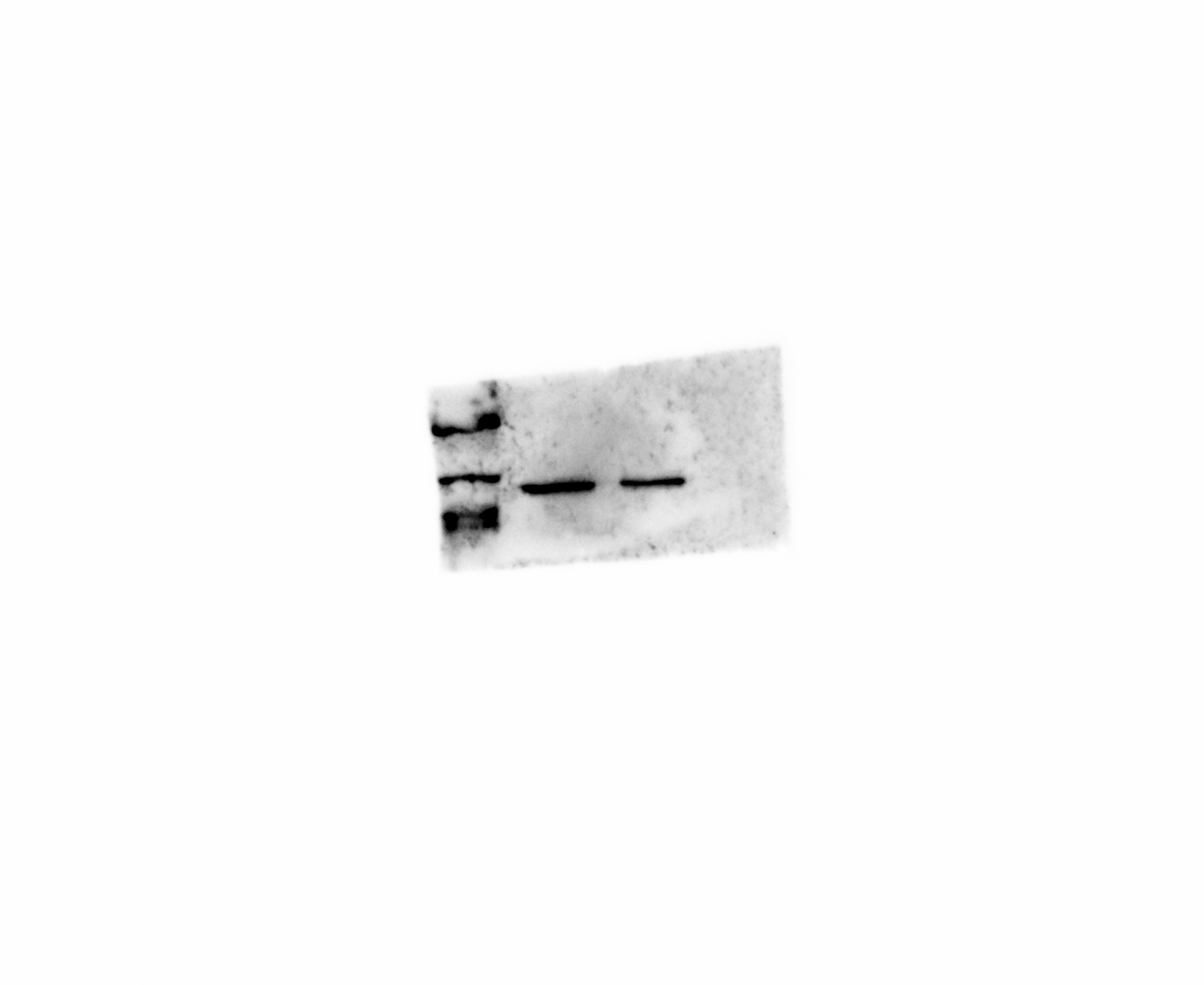

Supplement: Supplemental Material [file KBIE_A_2059614_SM8919.zip › Supplementary Material/Figure 1H/Saos-2 P-AKT.jpg]

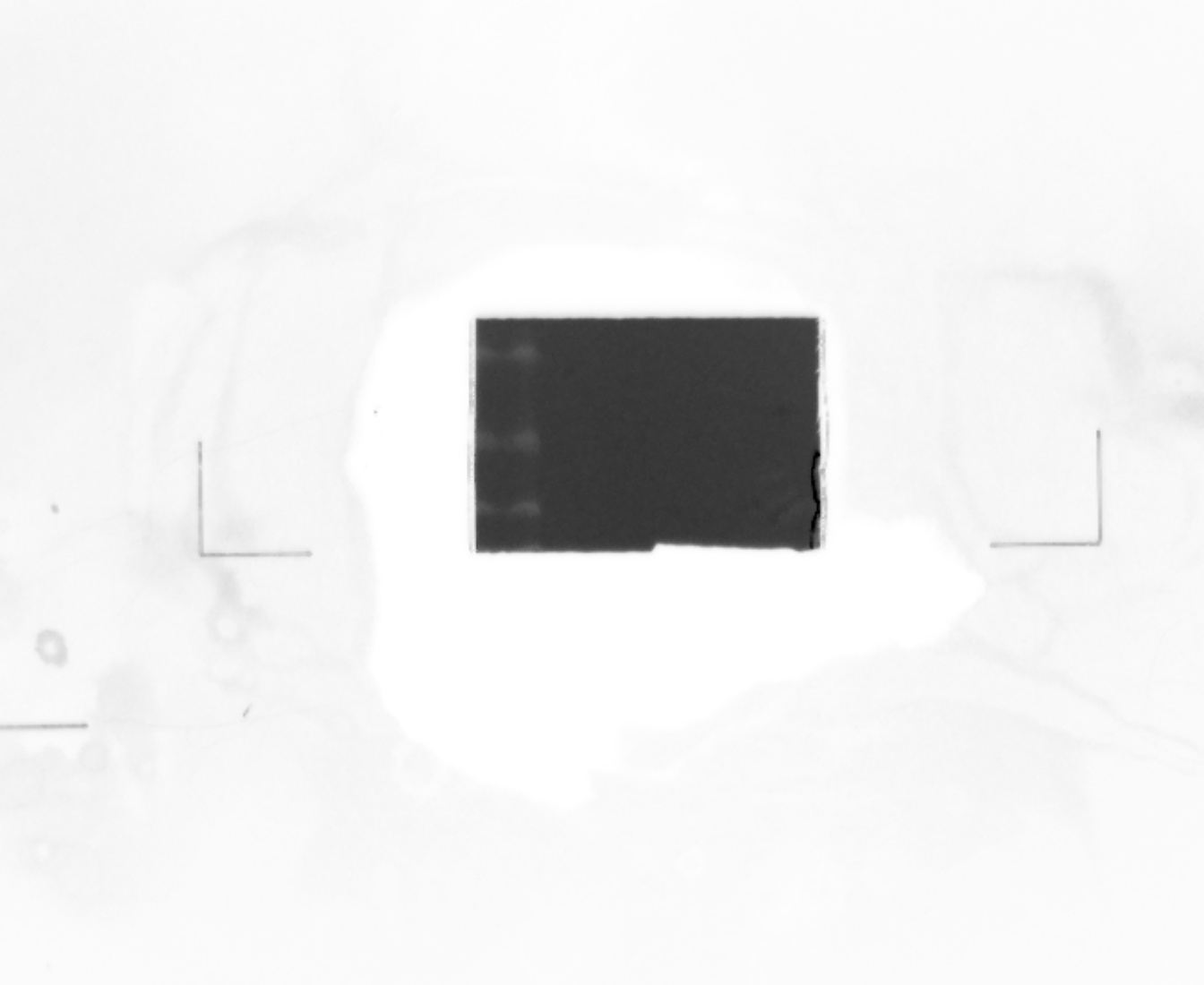

Supplement: Supplemental Material [file KBIE_A_2059614_SM8919.zip › Supplementary Material/Figure 1H/Saos-2 P-PI3K-bright field.jpg]

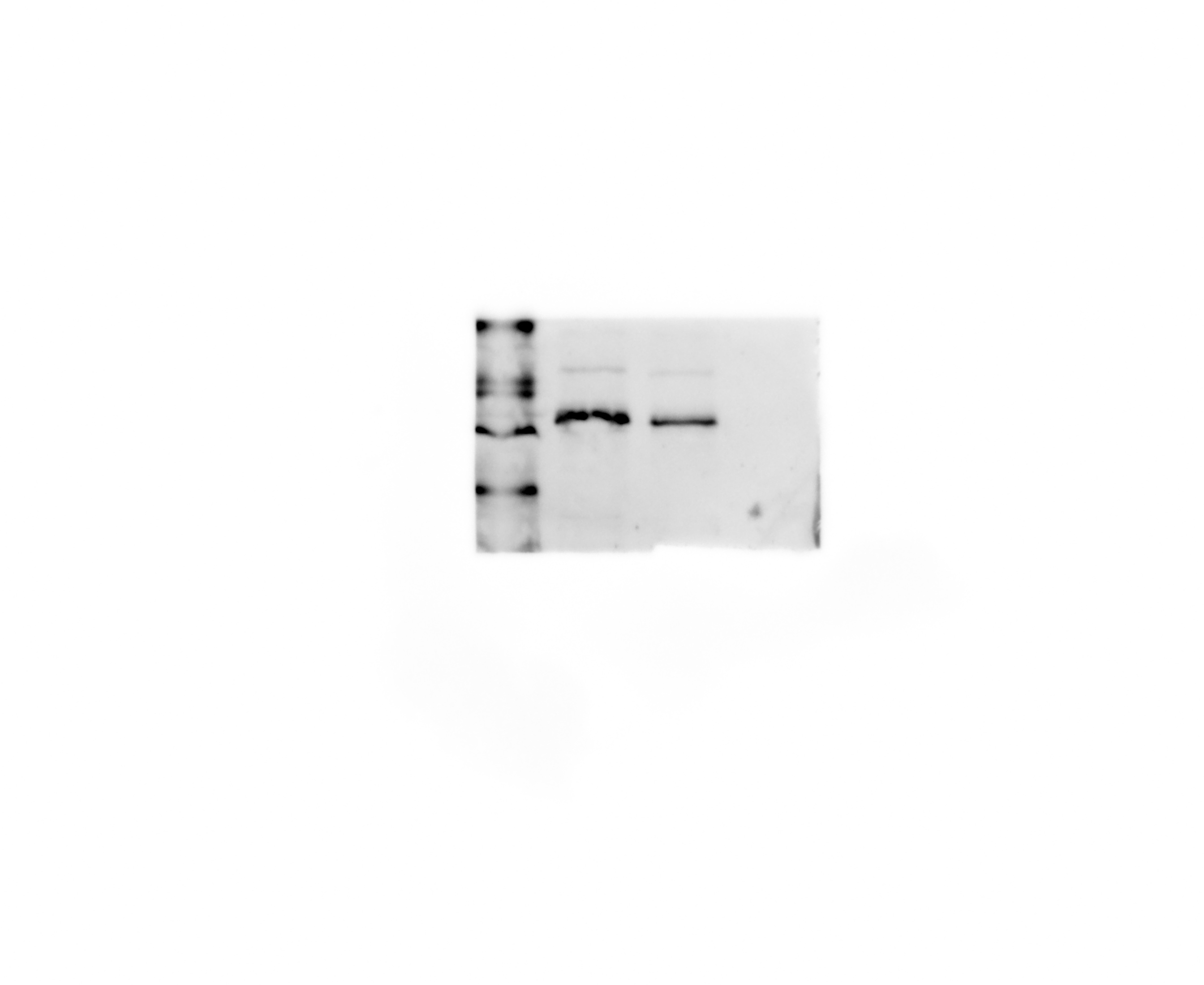

Supplement: Supplemental Material [file KBIE_A_2059614_SM8919.zip › Supplementary Material/Figure 1H/Saos-2 P-PI3K.jpg]

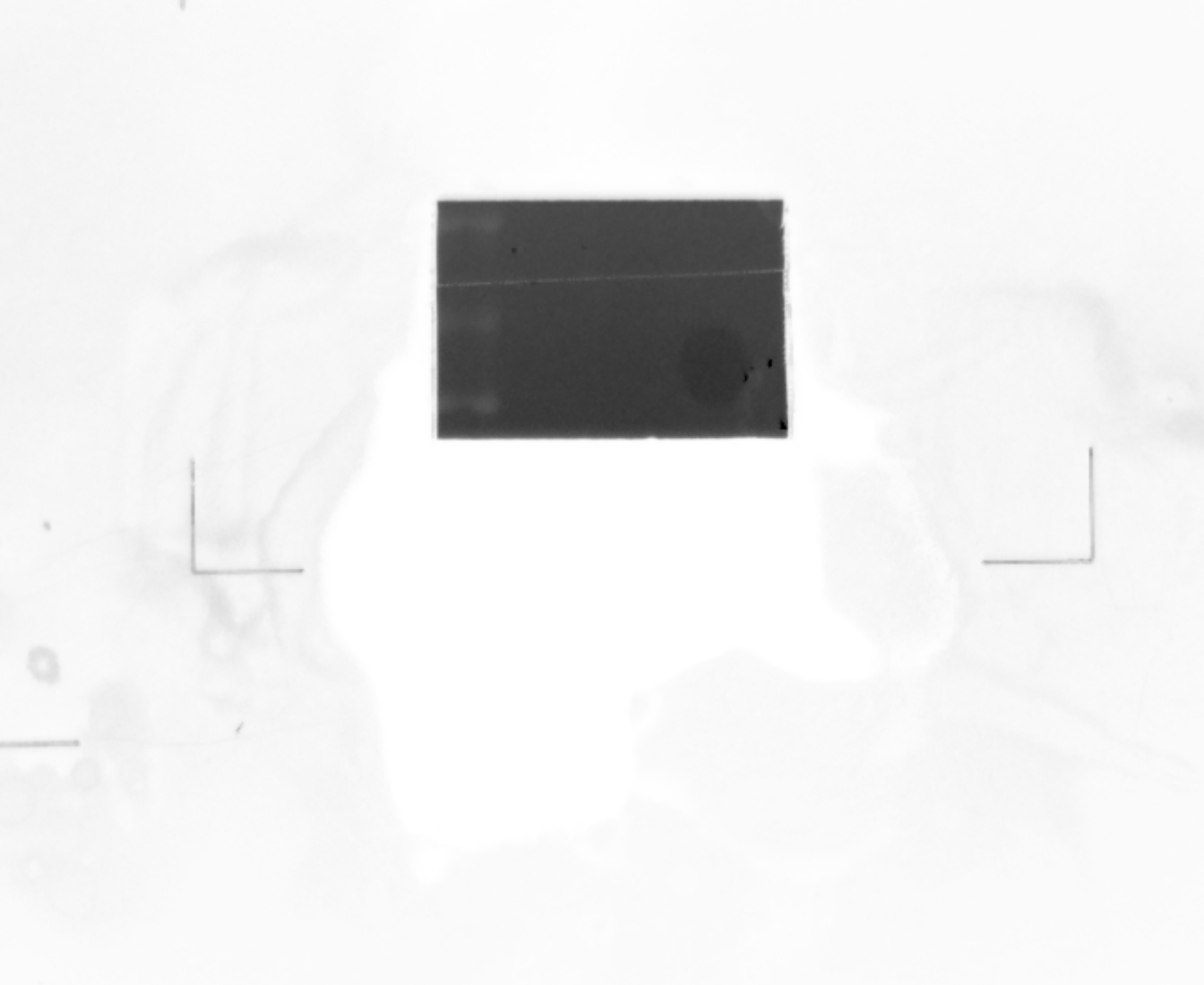

Supplement: Supplemental Material [file KBIE_A_2059614_SM8919.zip › Supplementary Material/Figure 1H/Saos-2 PI3K-bright field.jpg]

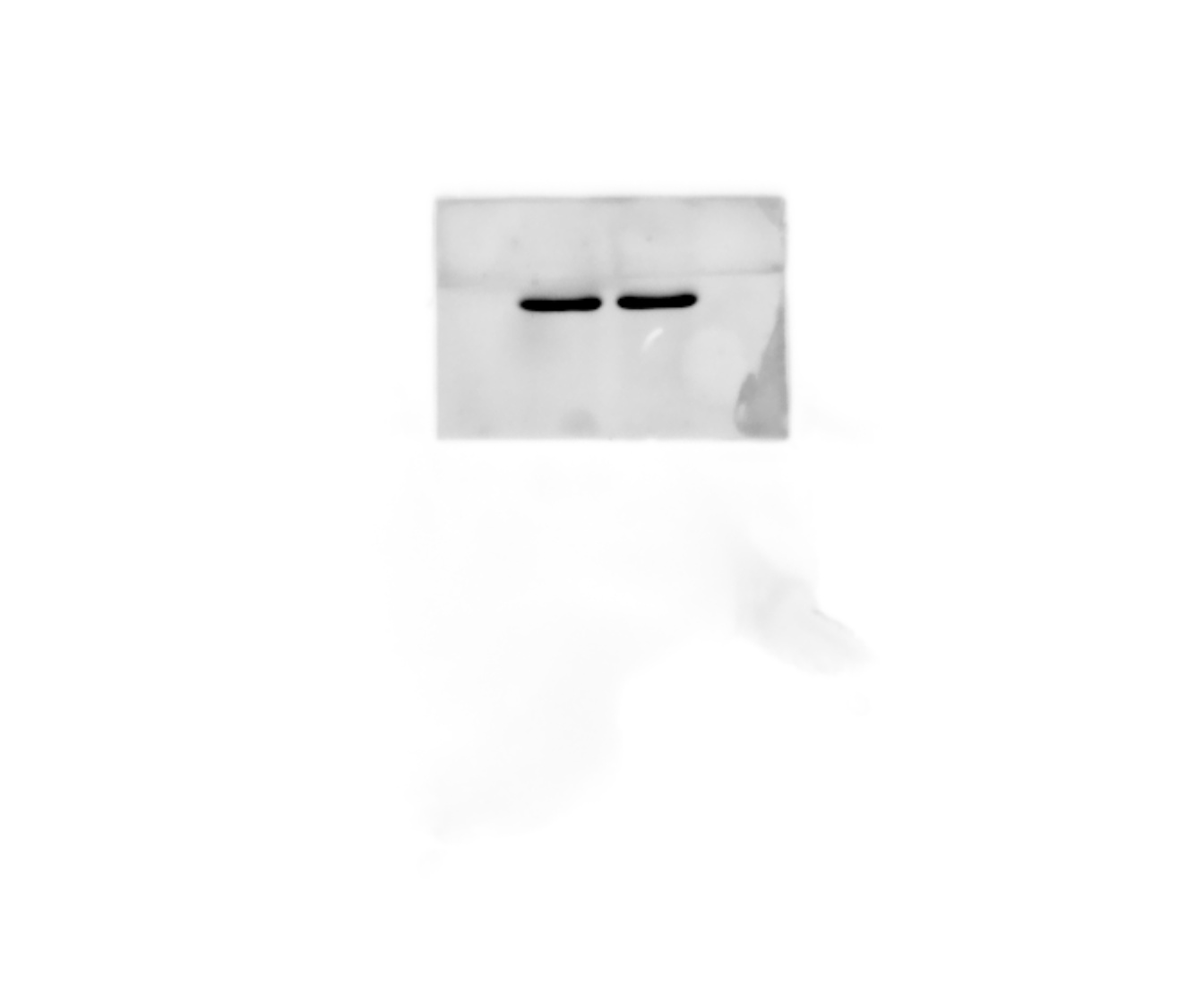

Supplement: Supplemental Material [file KBIE_A_2059614_SM8919.zip › Supplementary Material/Figure 1H/Saos-2 PI3K.jpg]

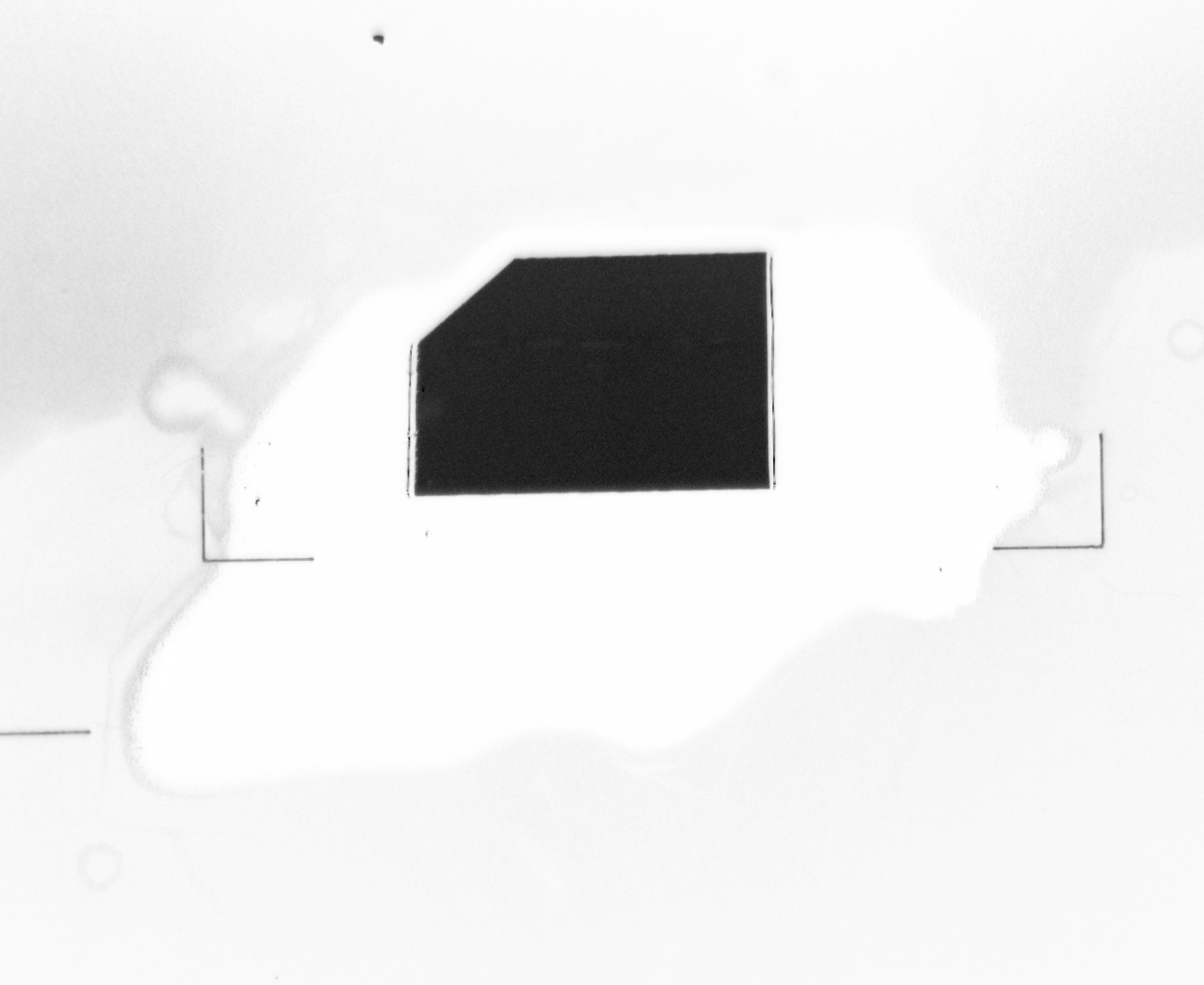

Supplement: Supplemental Material [file KBIE_A_2059614_SM8919.zip › Supplementary Material/Figure 4D/HOS AKT-bright field.jpg]

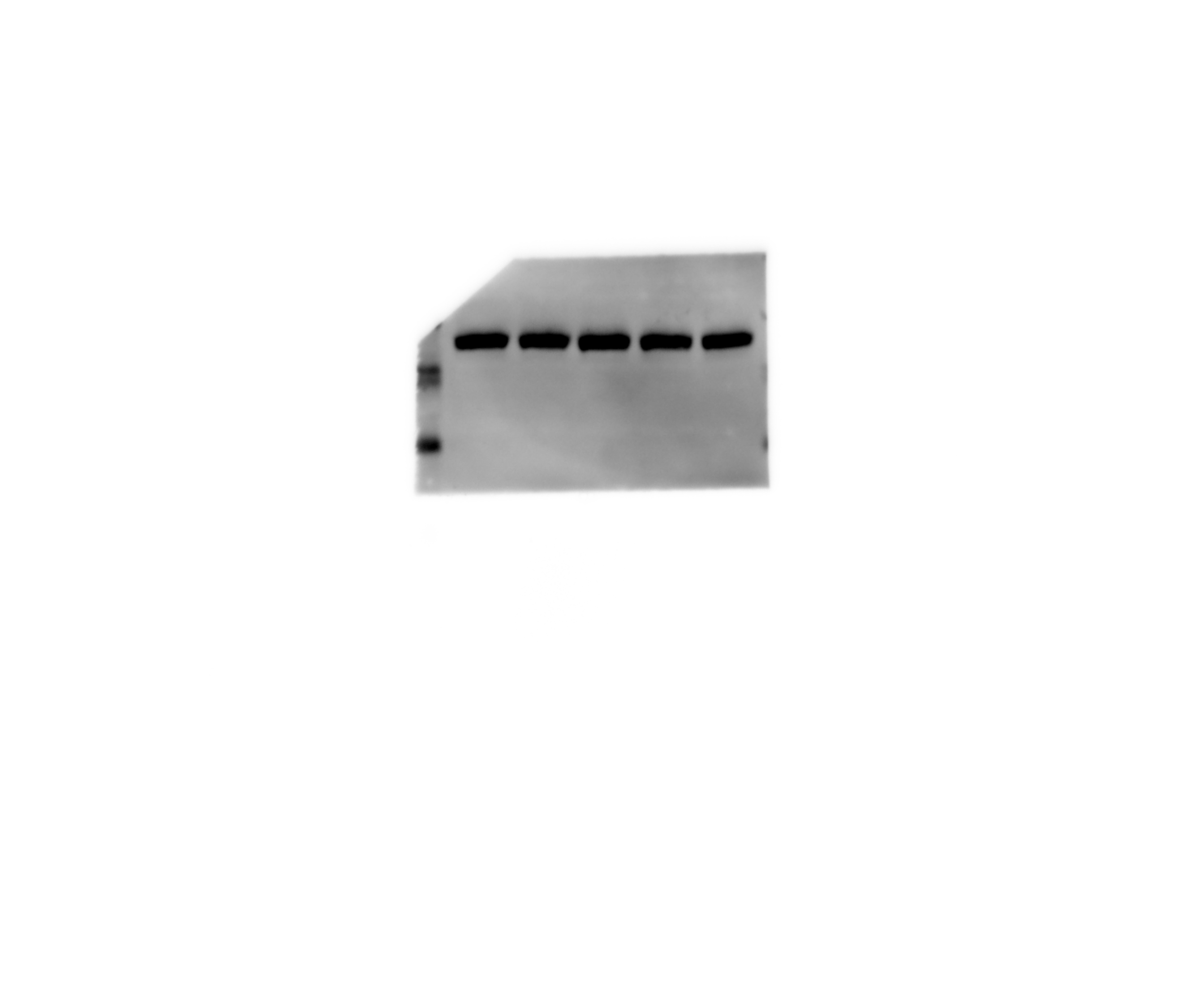

Supplement: Supplemental Material [file KBIE_A_2059614_SM8919.zip › Supplementary Material/Figure 4D/HOS AKT.jpg]

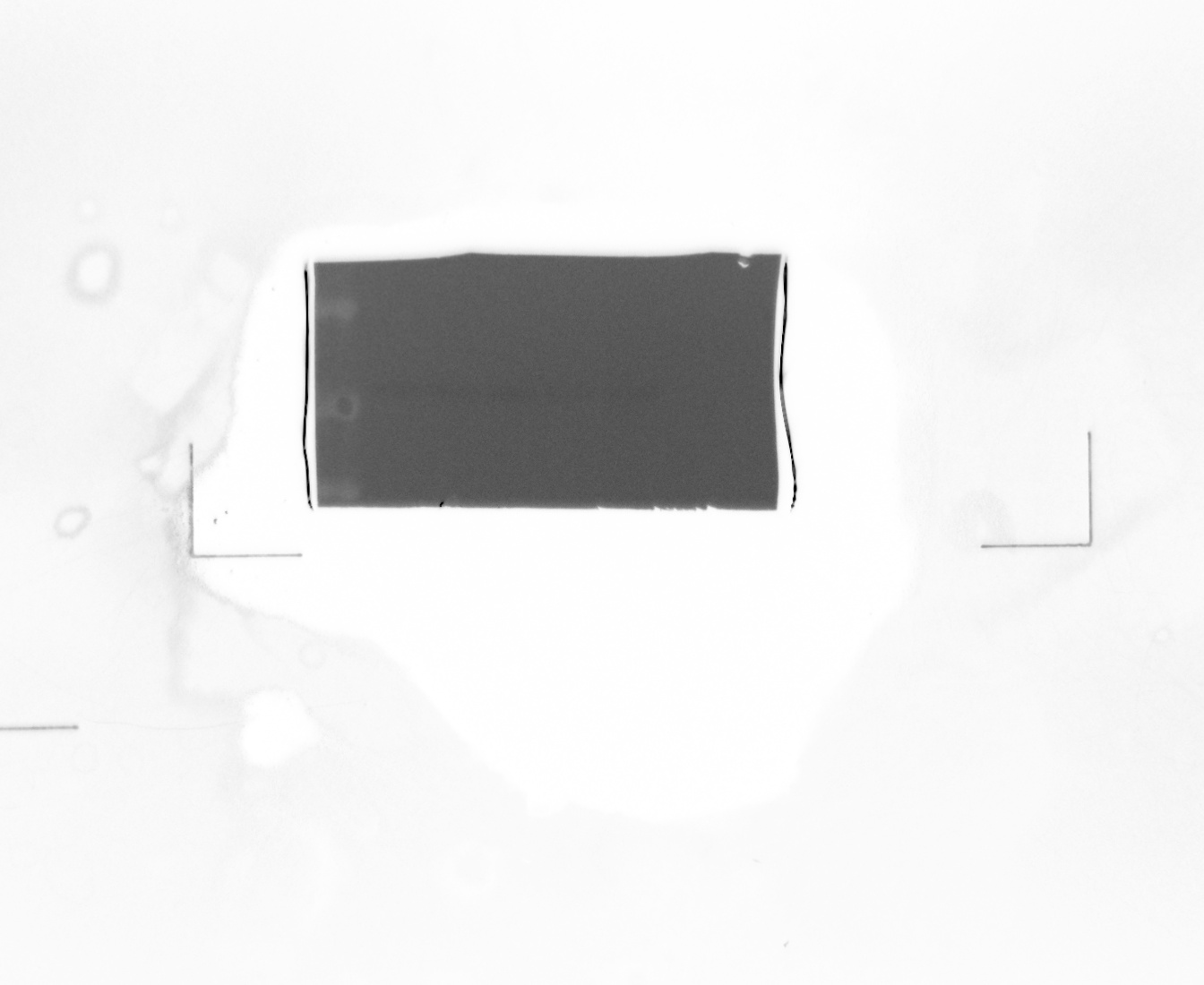

Supplement: Supplemental Material [file KBIE_A_2059614_SM8919.zip › Supplementary Material/Figure 4D/HOS GAPDH-bright field.jpg]

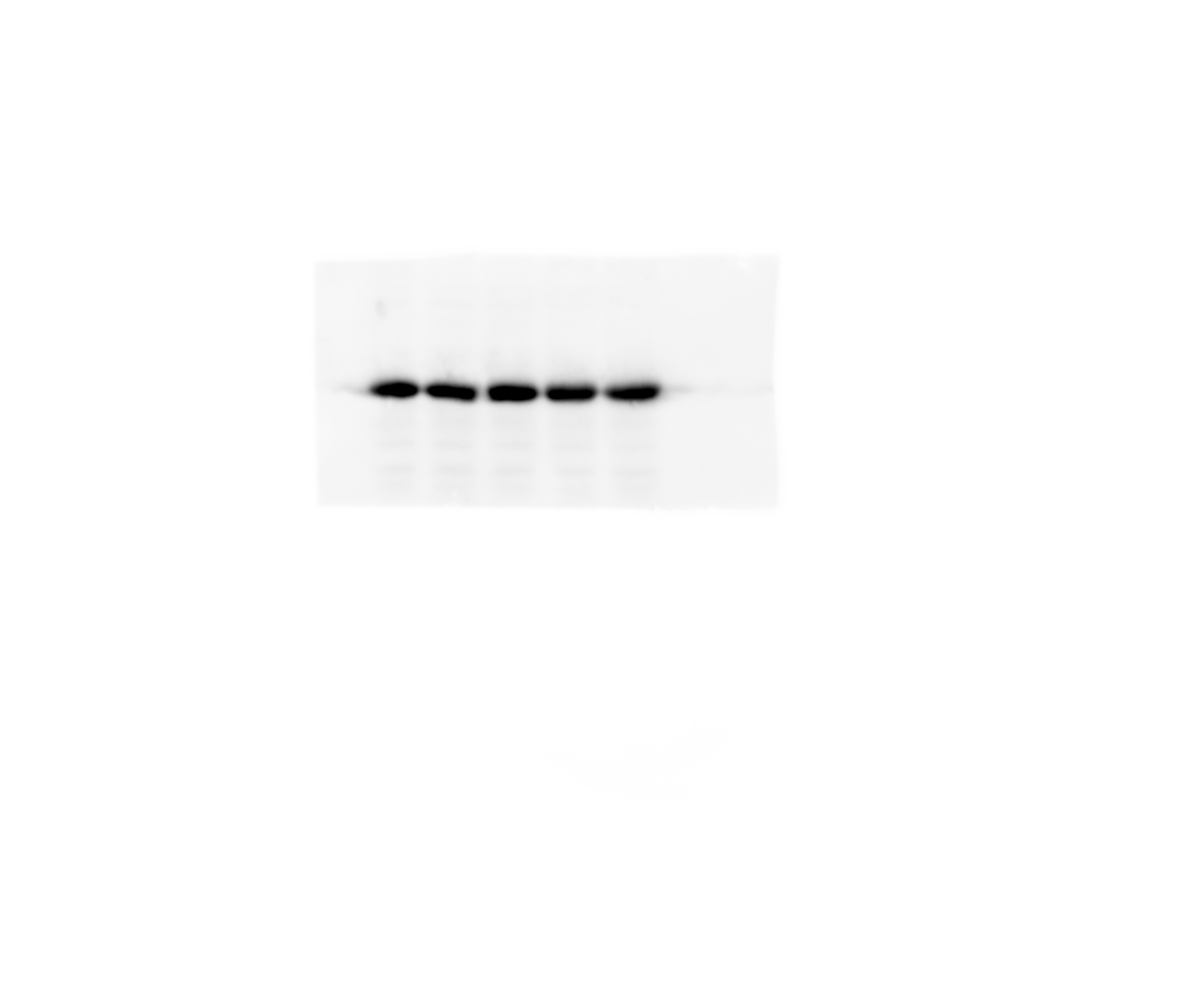

Supplement: Supplemental Material [file KBIE_A_2059614_SM8919.zip › Supplementary Material/Figure 4D/HOS GAPDH.jpg]

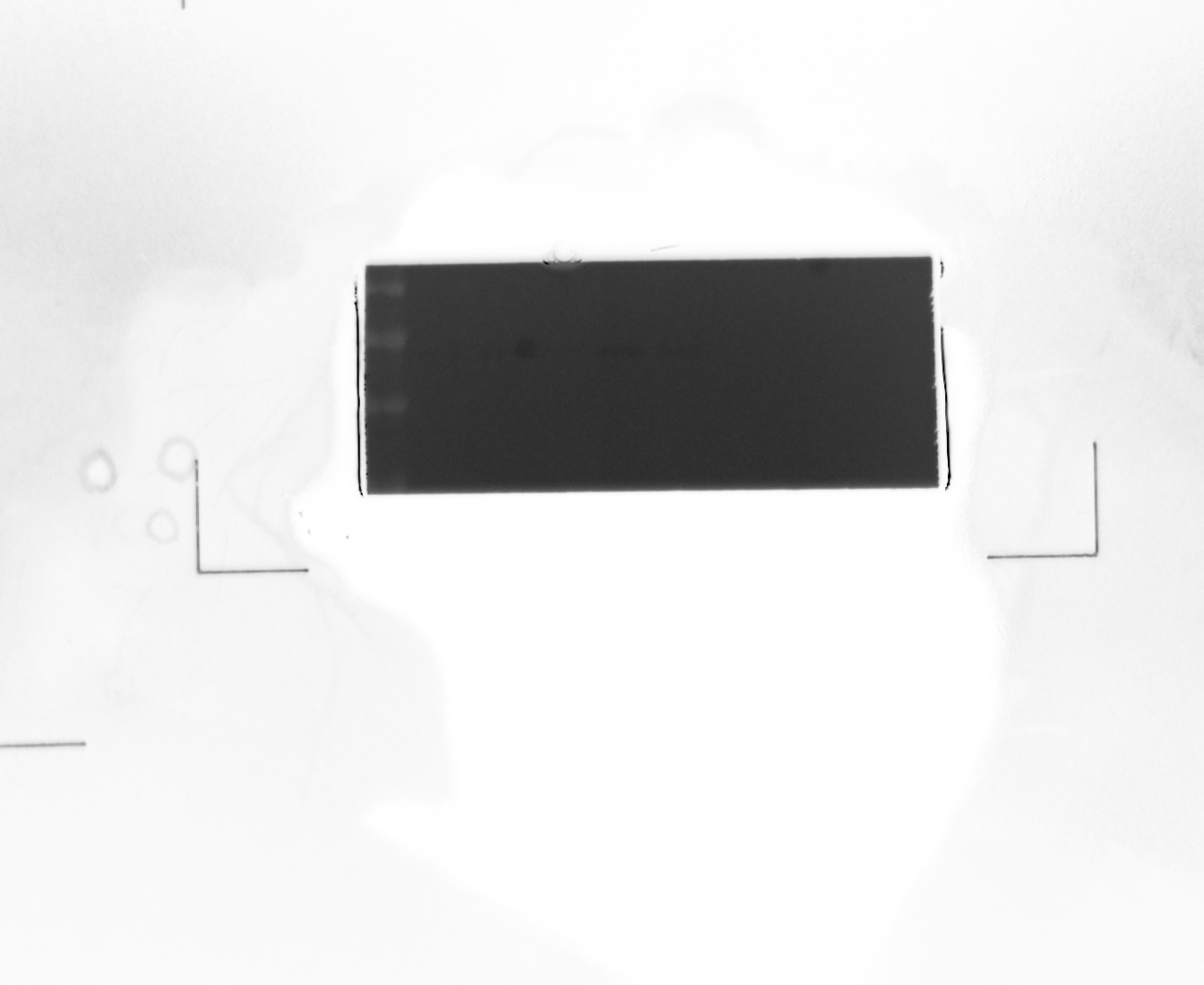

Supplement: Supplemental Material [file KBIE_A_2059614_SM8919.zip › Supplementary Material/Figure 4D/HOS P-AKT-bright field.jpg]

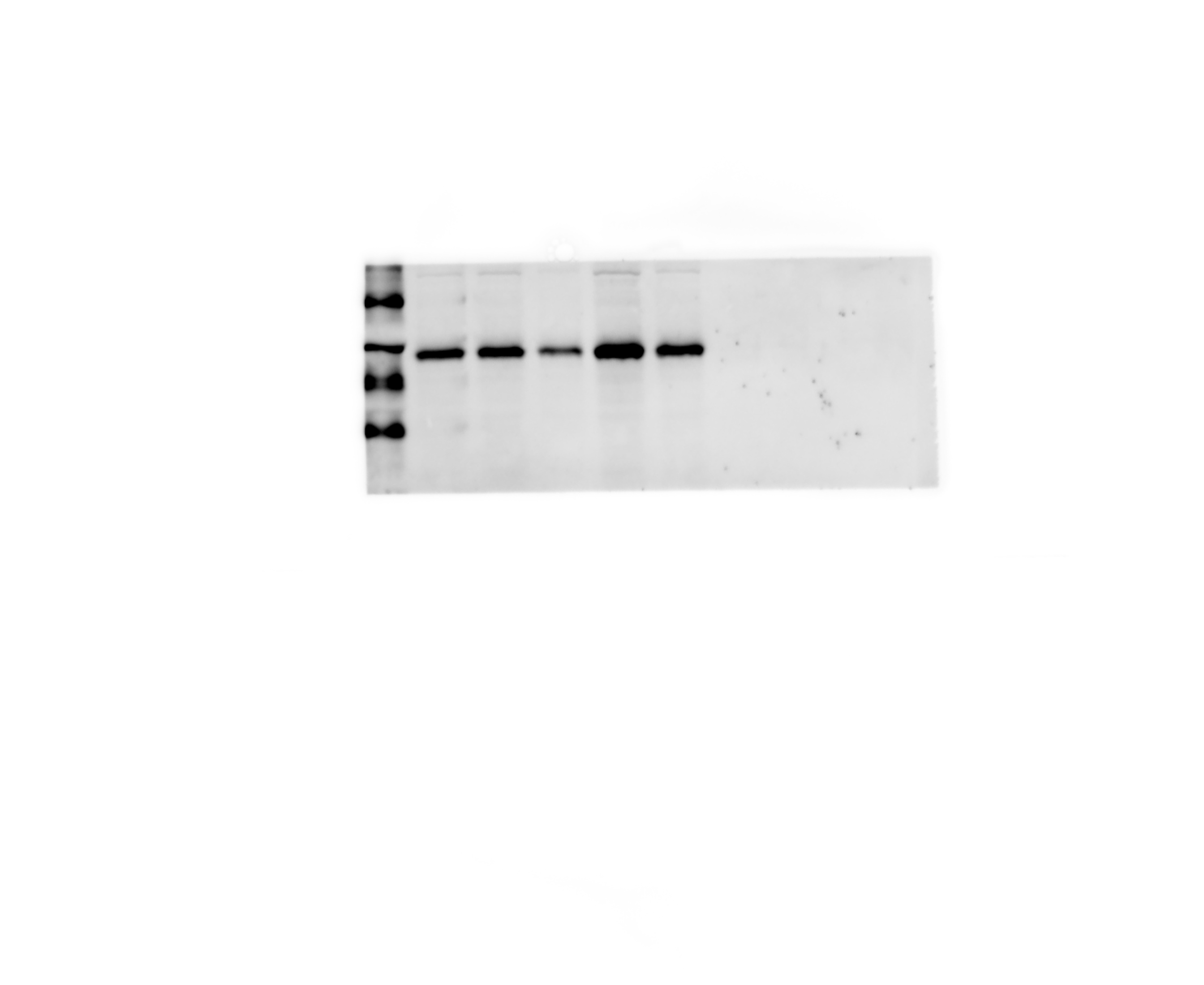

Supplement: Supplemental Material [file KBIE_A_2059614_SM8919.zip › Supplementary Material/Figure 4D/HOS P-AKT.jpg]

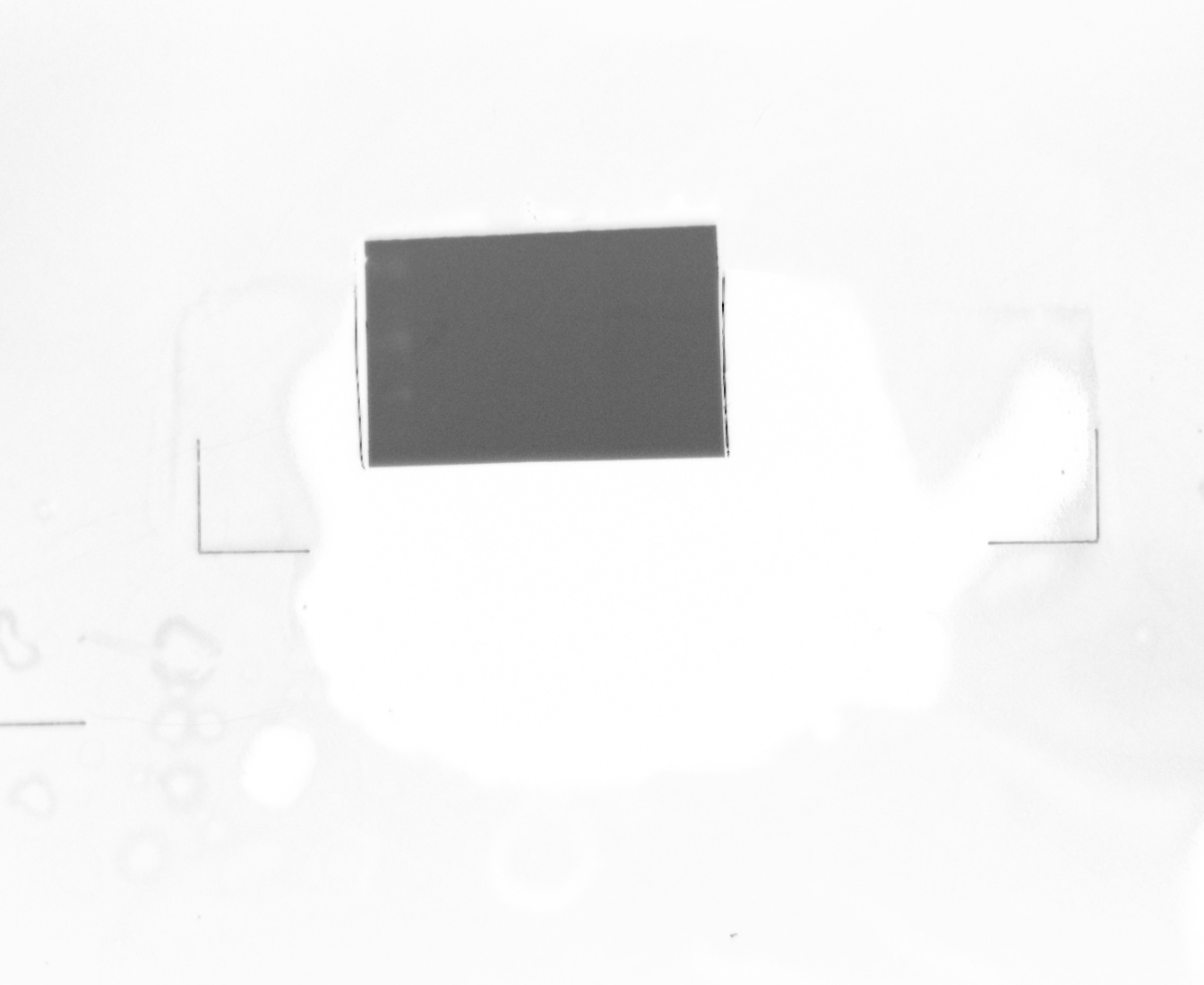

Supplement: Supplemental Material [file KBIE_A_2059614_SM8919.zip › Supplementary Material/Figure 4D/HOS P-PI3K-bright field.jpg]

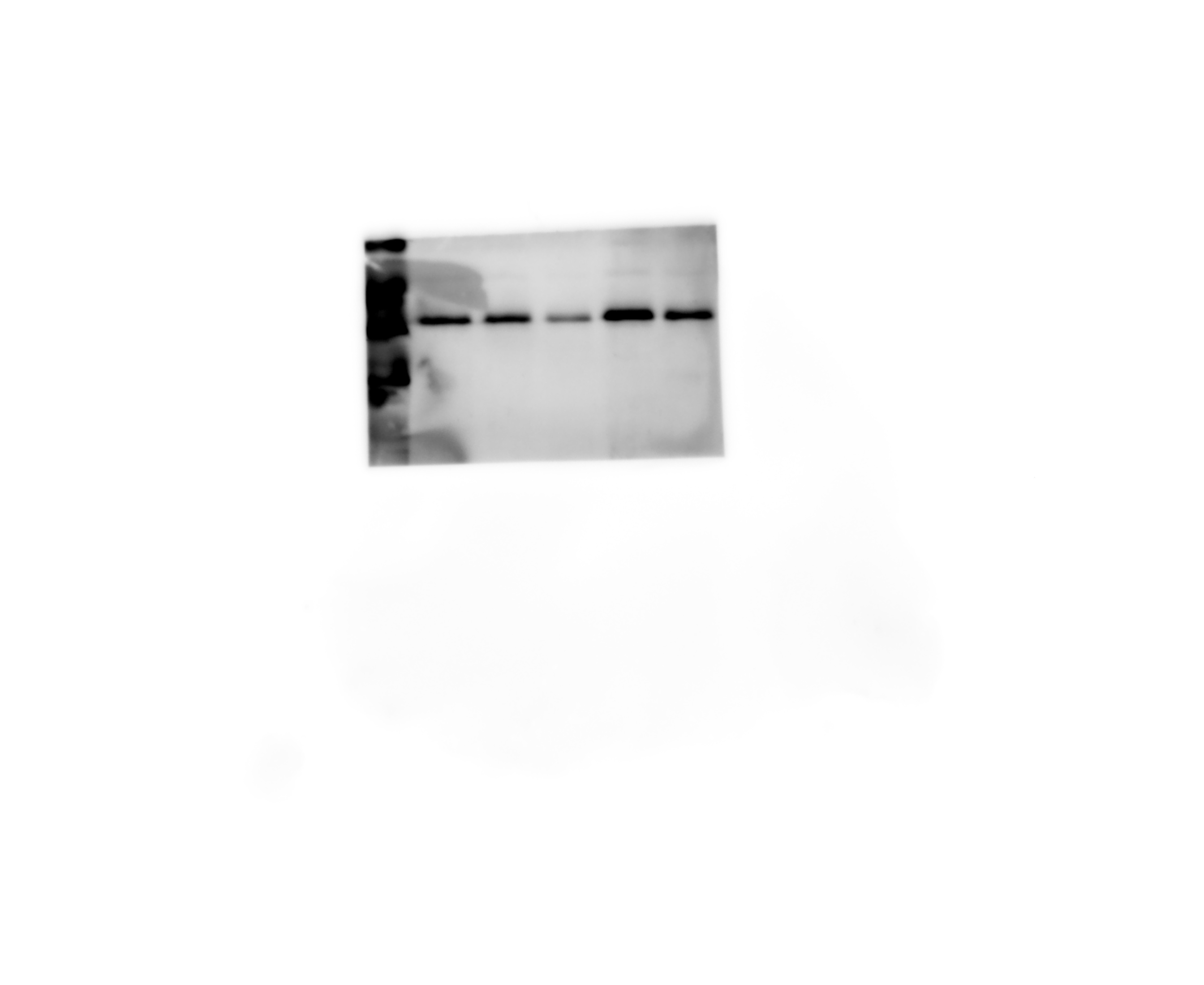

Supplement: Supplemental Material [file KBIE_A_2059614_SM8919.zip › Supplementary Material/Figure 4D/HOS P-PI3K.jpg]

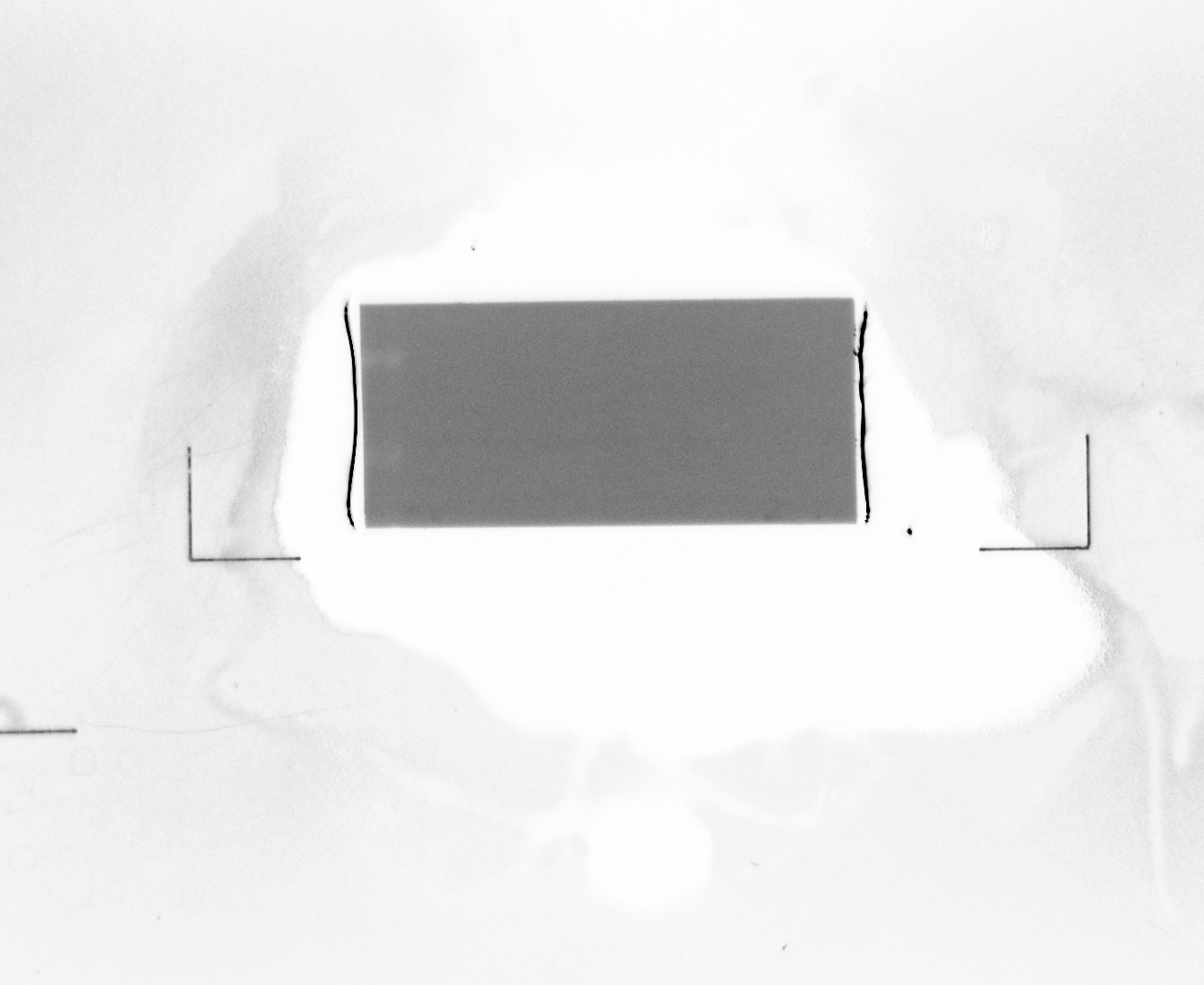

Supplement: Supplemental Material [file KBIE_A_2059614_SM8919.zip › Supplementary Material/Figure 4D/HOS PI3K-bright field.jpg]

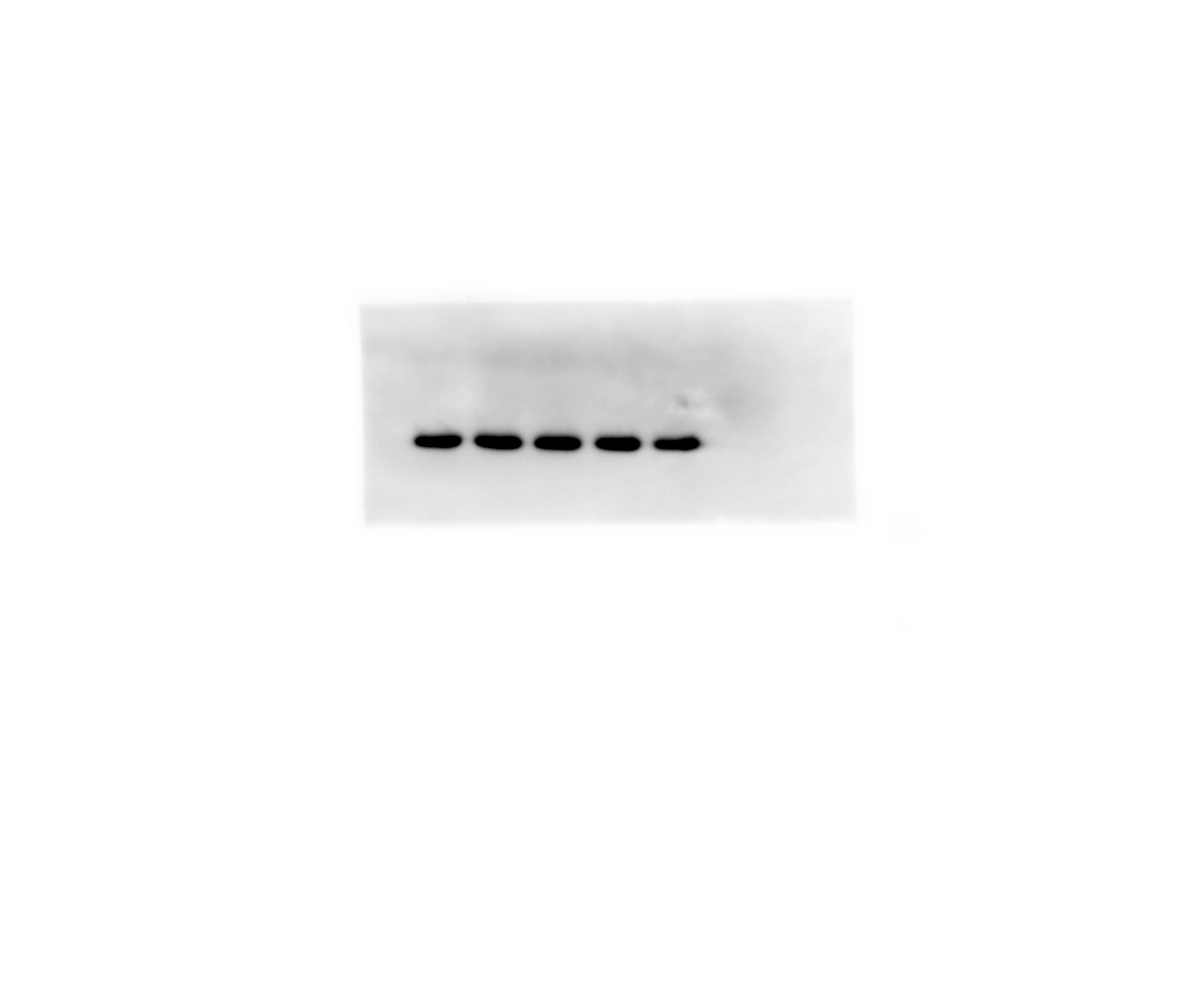

Supplement: Supplemental Material [file KBIE_A_2059614_SM8919.zip › Supplementary Material/Figure 4D/HOS PI3K.jpg]

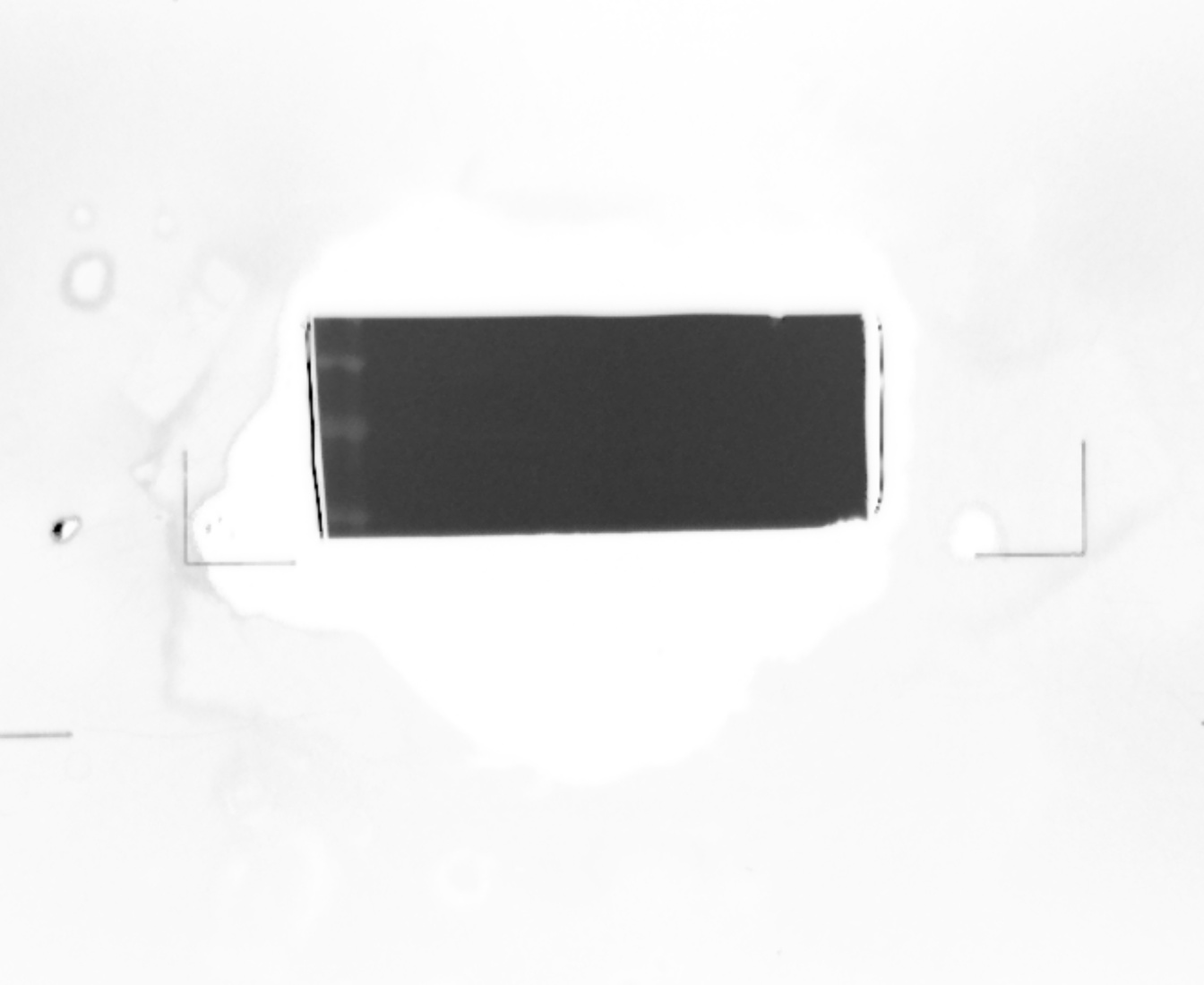

Supplement: Supplemental Material [file KBIE_A_2059614_SM8919.zip › Supplementary Material/Figure 4D/Saos-2 AKT-bright field.jpg]

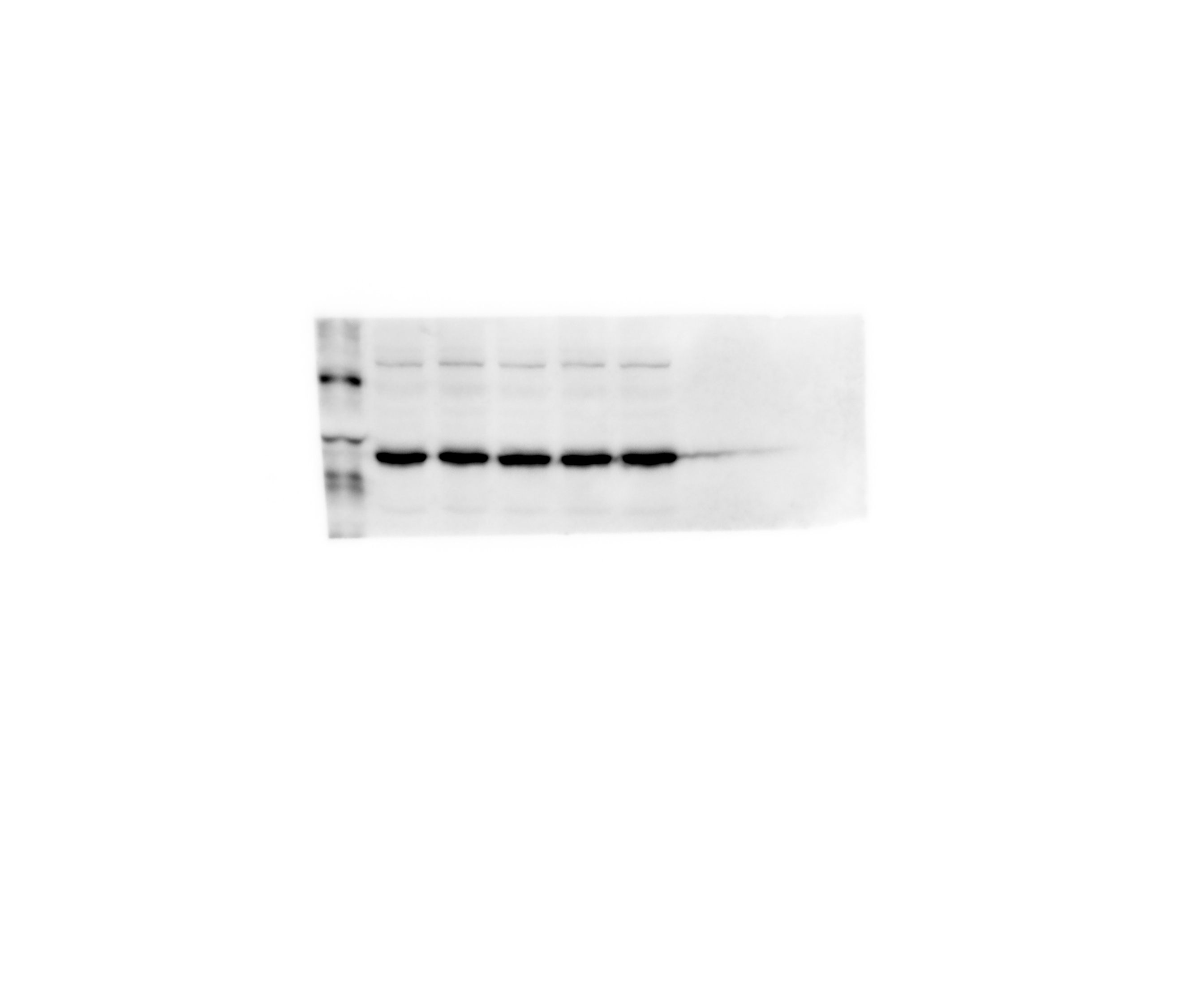

Supplement: Supplemental Material [file KBIE_A_2059614_SM8919.zip › Supplementary Material/Figure 4D/Saos-2 AKT.jpg]

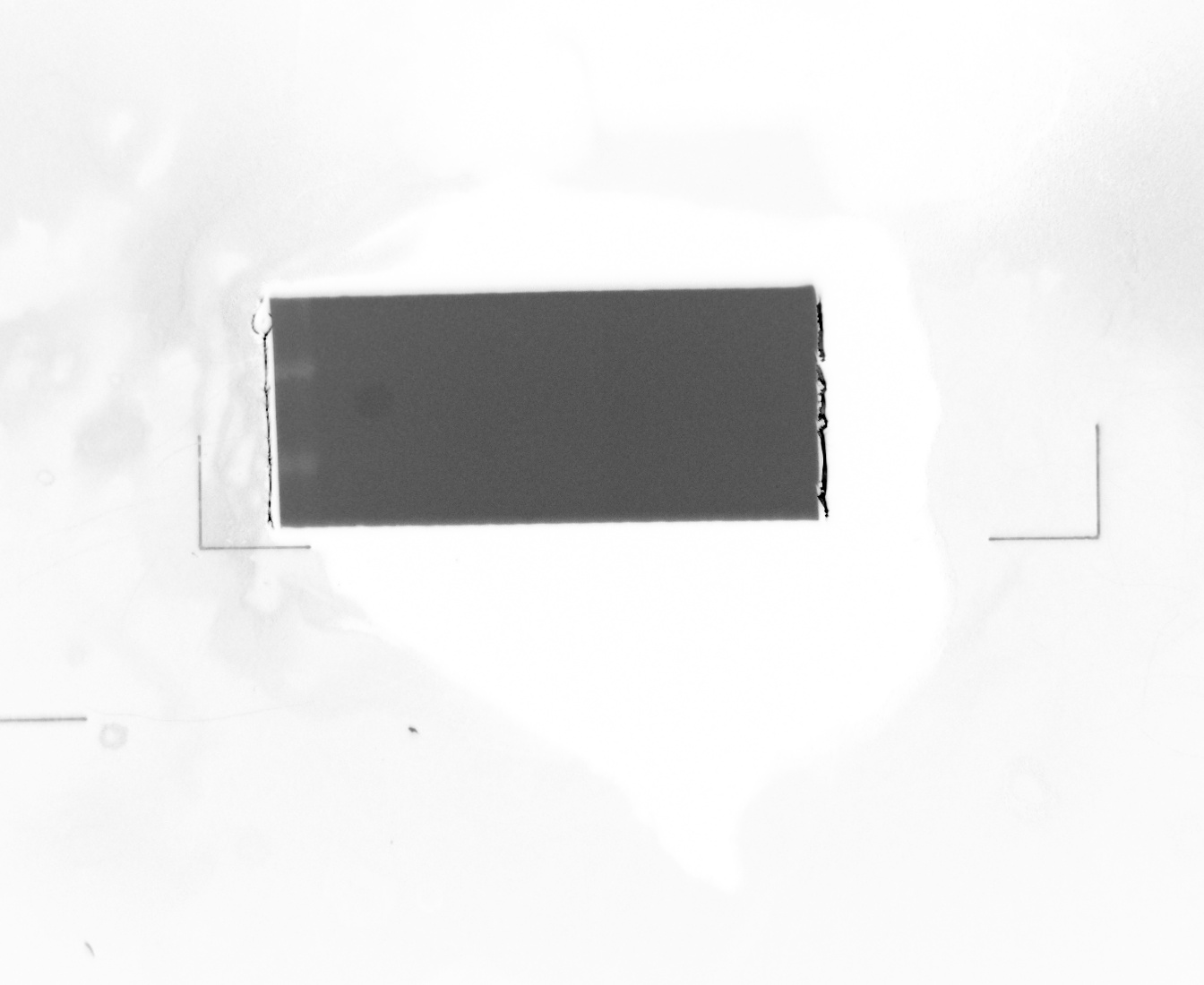

Supplement: Supplemental Material [file KBIE_A_2059614_SM8919.zip › Supplementary Material/Figure 4D/Saos-2 GAPDH-bright field.jpg]

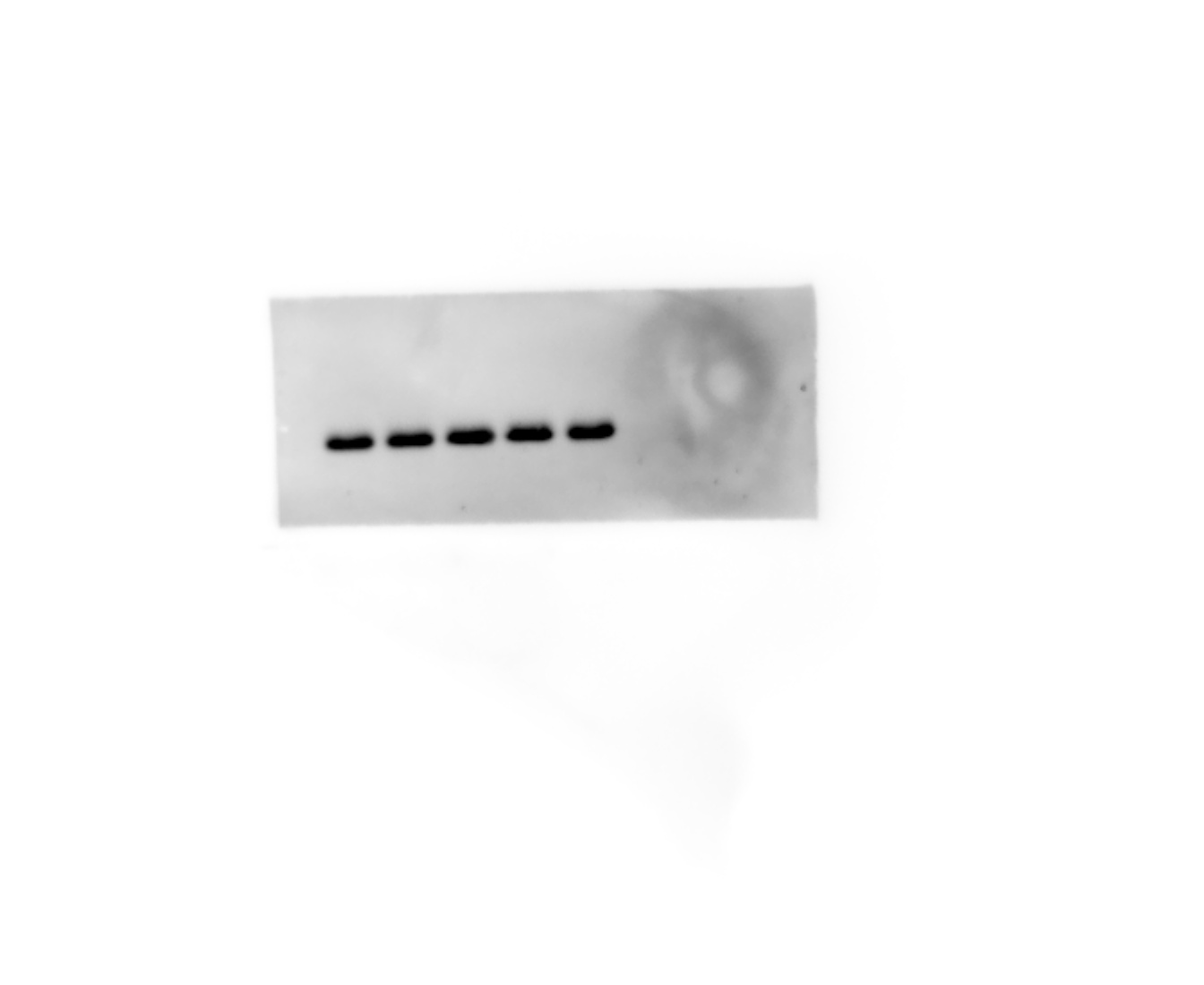

Supplement: Supplemental Material [file KBIE_A_2059614_SM8919.zip › Supplementary Material/Figure 4D/Saos-2 GAPDH.jpg]

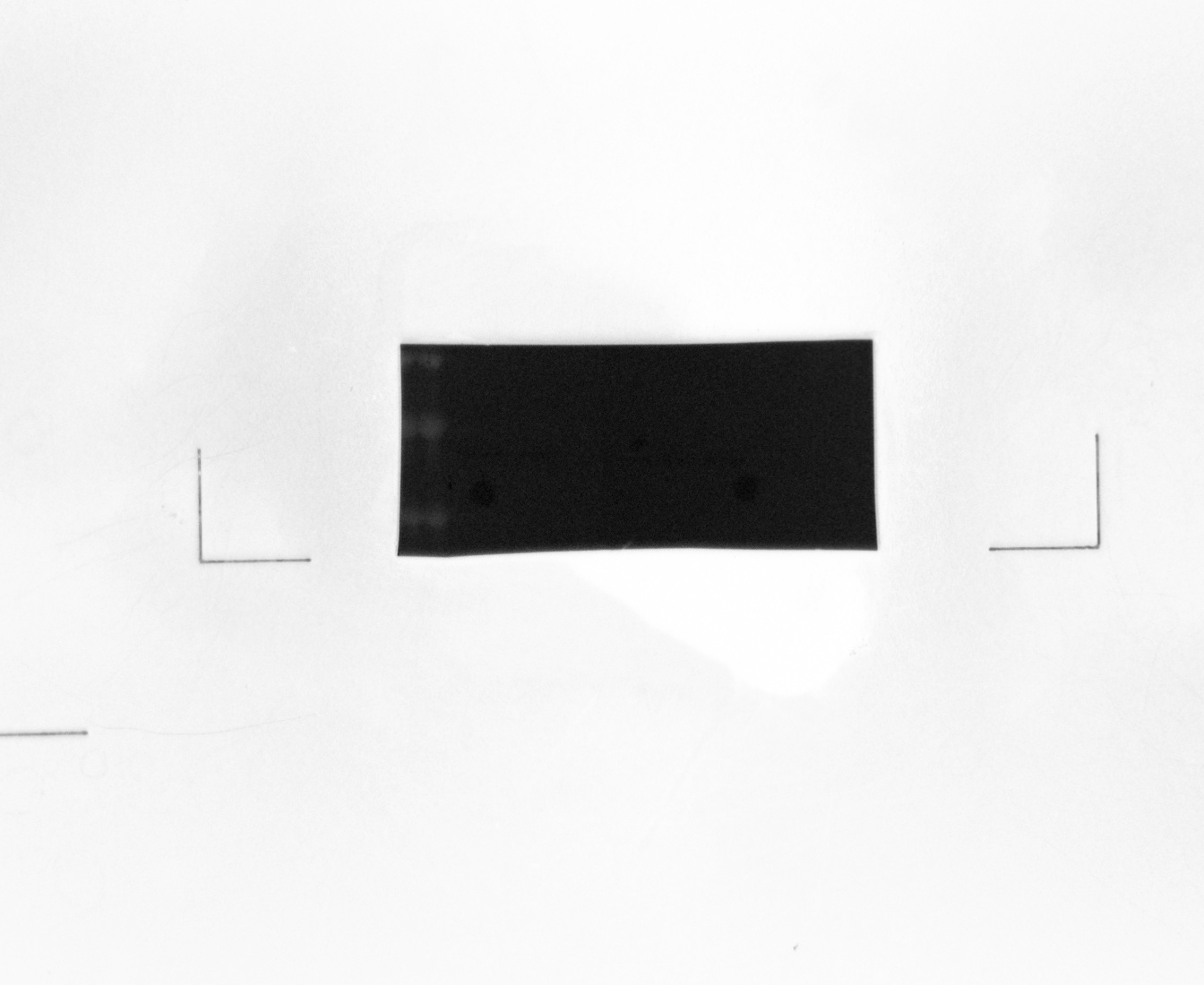

Supplement: Supplemental Material [file KBIE_A_2059614_SM8919.zip › Supplementary Material/Figure 4D/Saos-2 P-AKT-bright field.jpg]

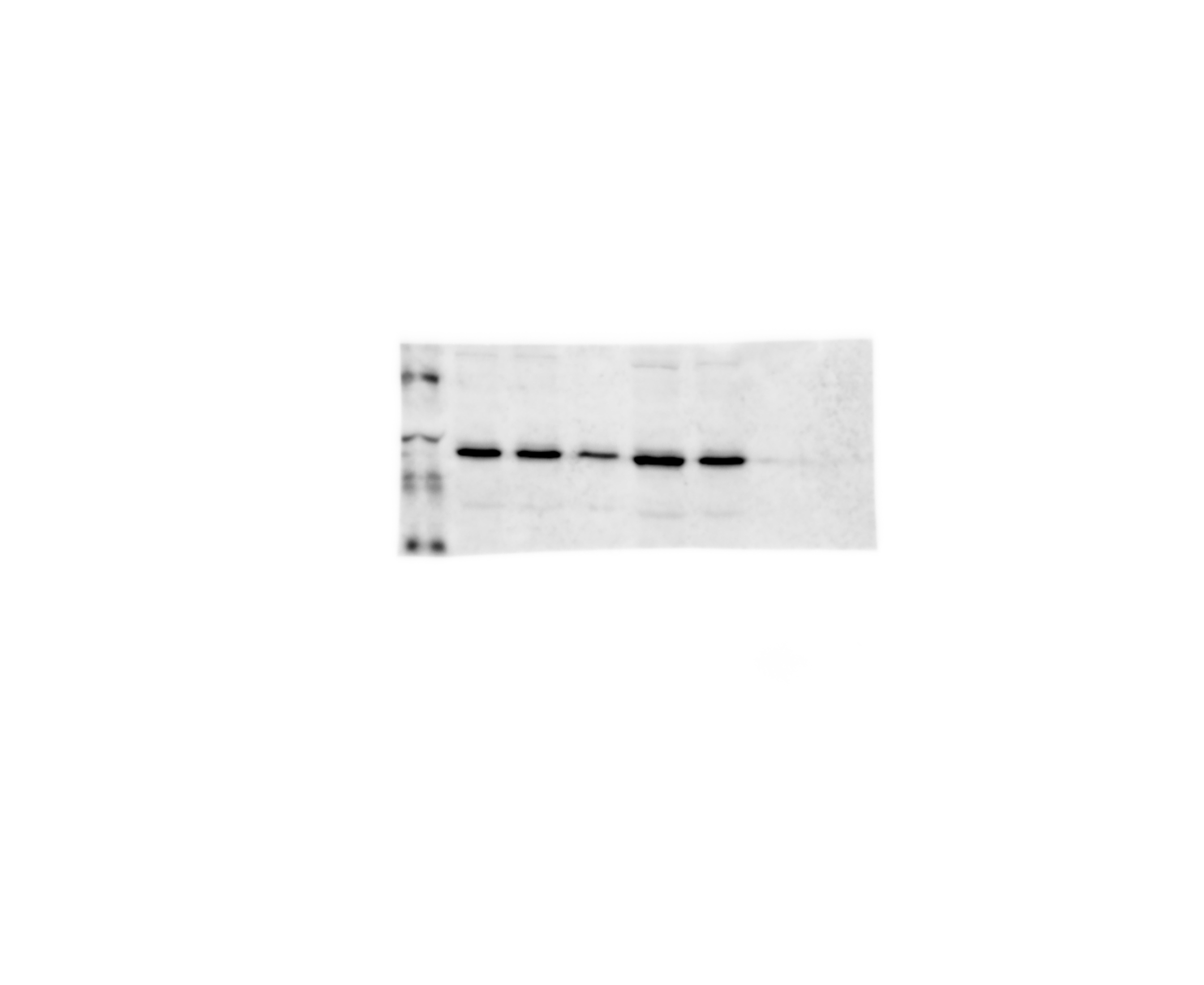

Supplement: Supplemental Material [file KBIE_A_2059614_SM8919.zip › Supplementary Material/Figure 4D/Saos-2 P-AKT.jpg]

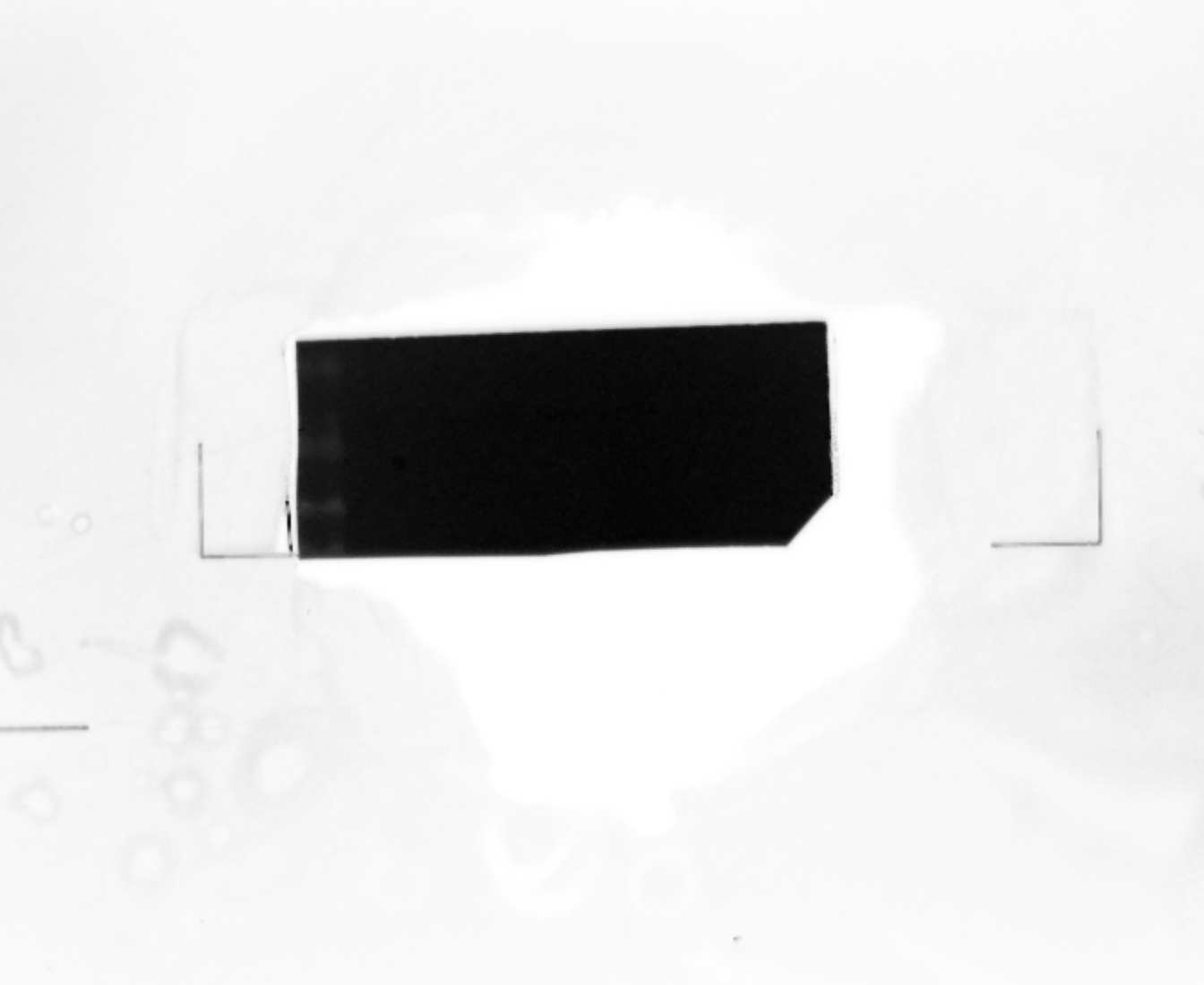

Supplement: Supplemental Material [file KBIE_A_2059614_SM8919.zip › Supplementary Material/Figure 4D/Saos-2 P-PI3K-bright field.jpg]

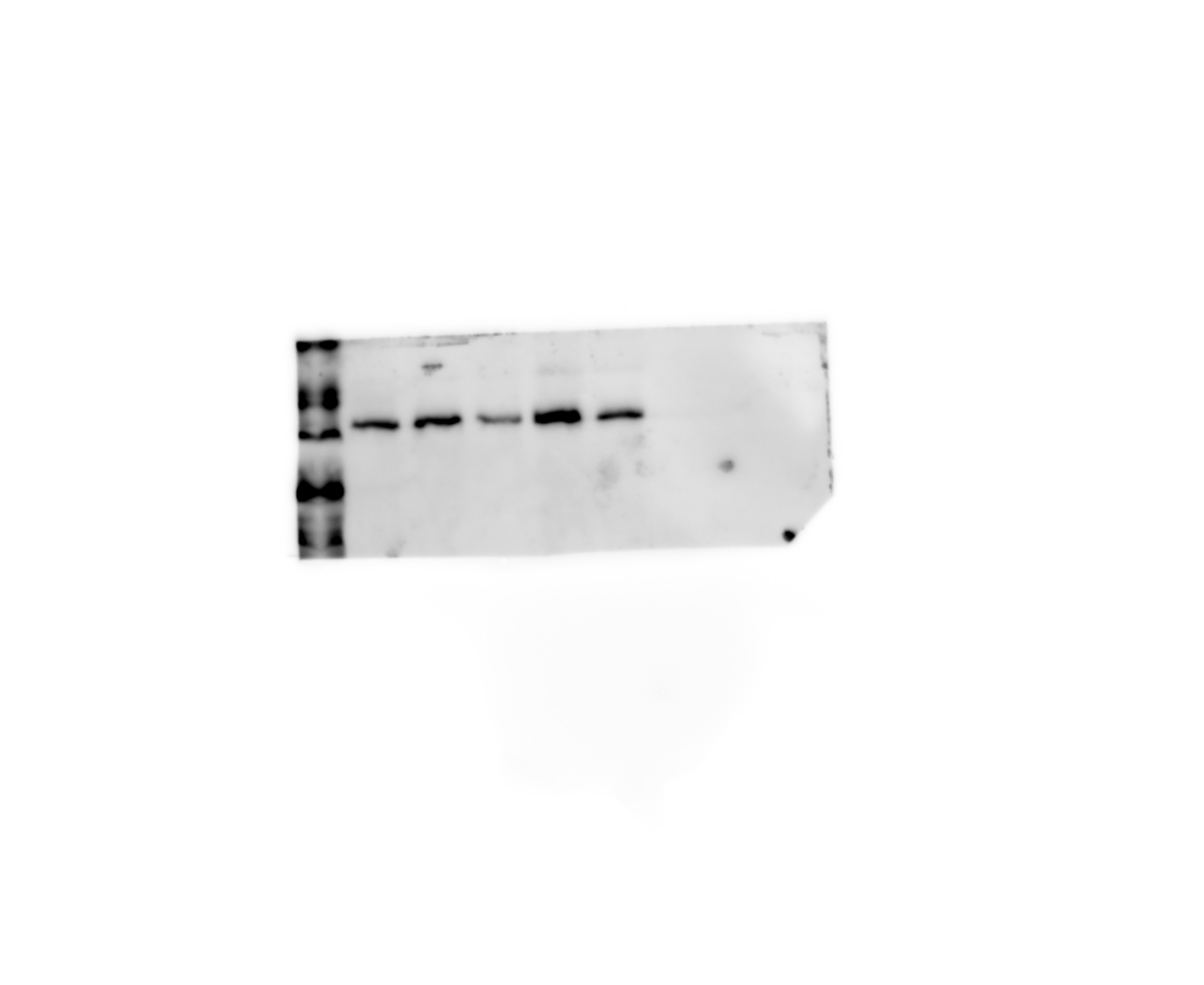

Supplement: Supplemental Material [file KBIE_A_2059614_SM8919.zip › Supplementary Material/Figure 4D/Saos-2 P-PI3K.jpg]

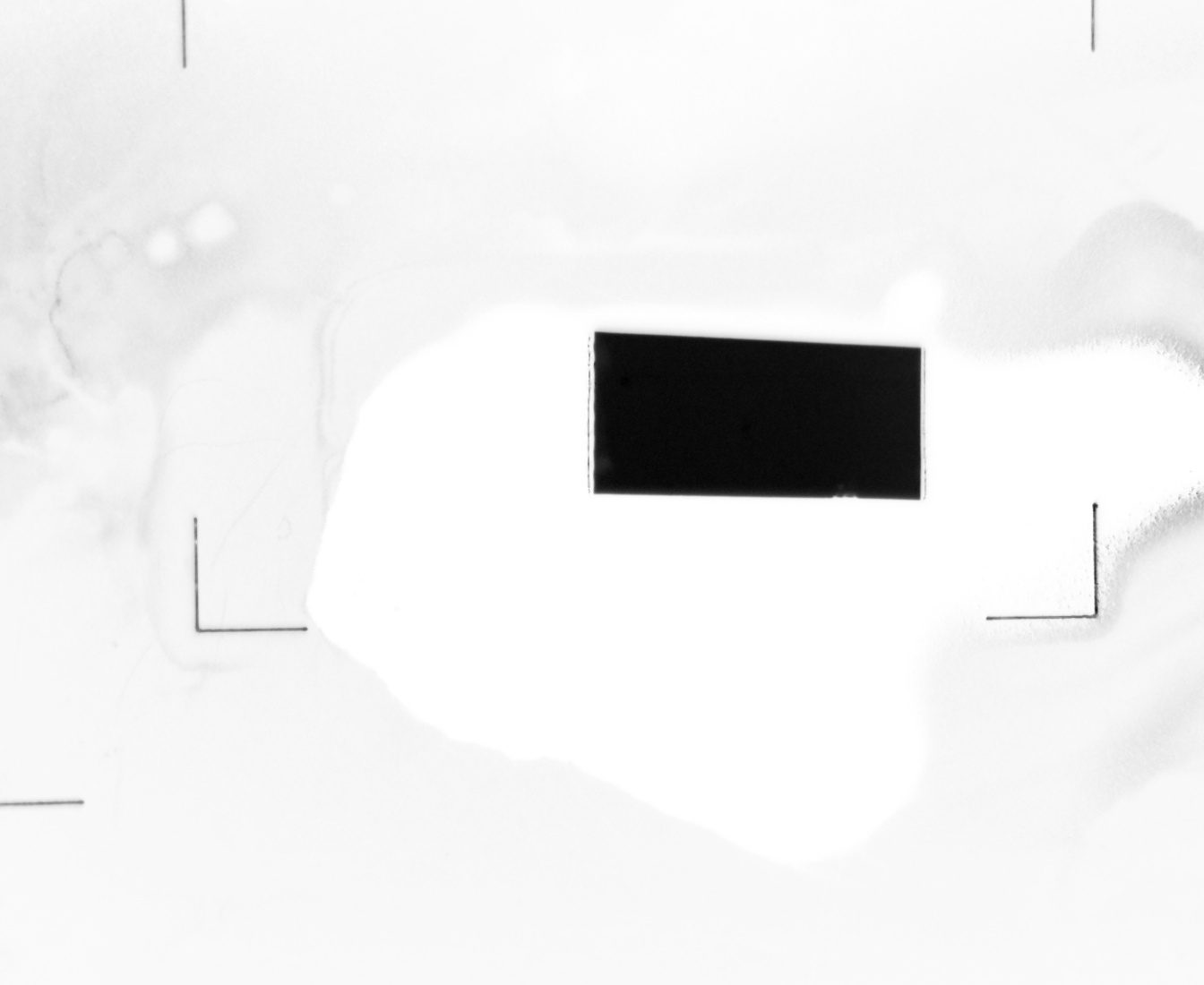

Supplement: Supplemental Material [file KBIE_A_2059614_SM8919.zip › Supplementary Material/Figure 4D/Saos-2 PI3K-bright field.jpg]

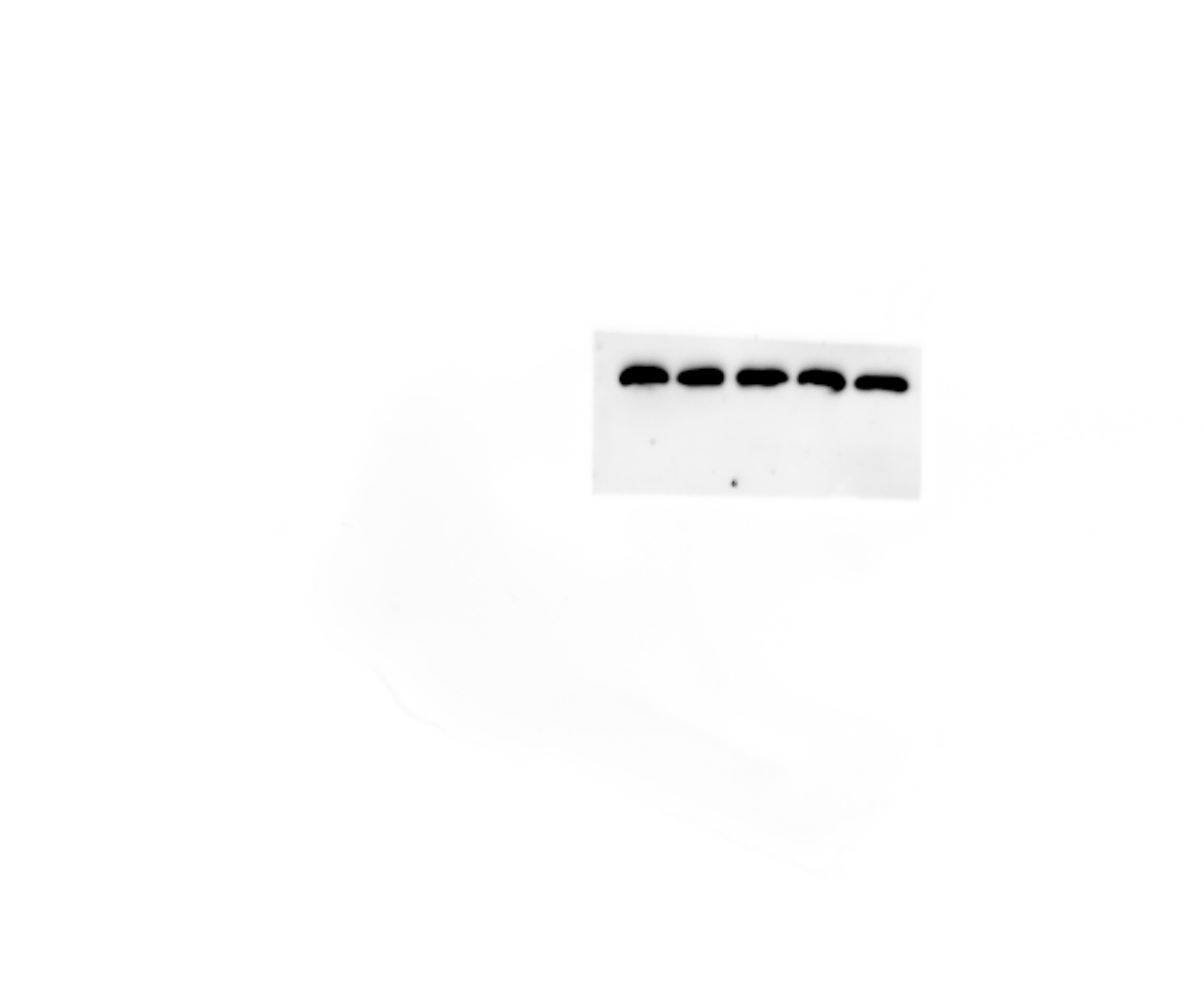

Supplement: Supplemental Material [file KBIE_A_2059614_SM8919.zip › Supplementary Material/Figure 4D/Saos-2 PI3K.jpg]

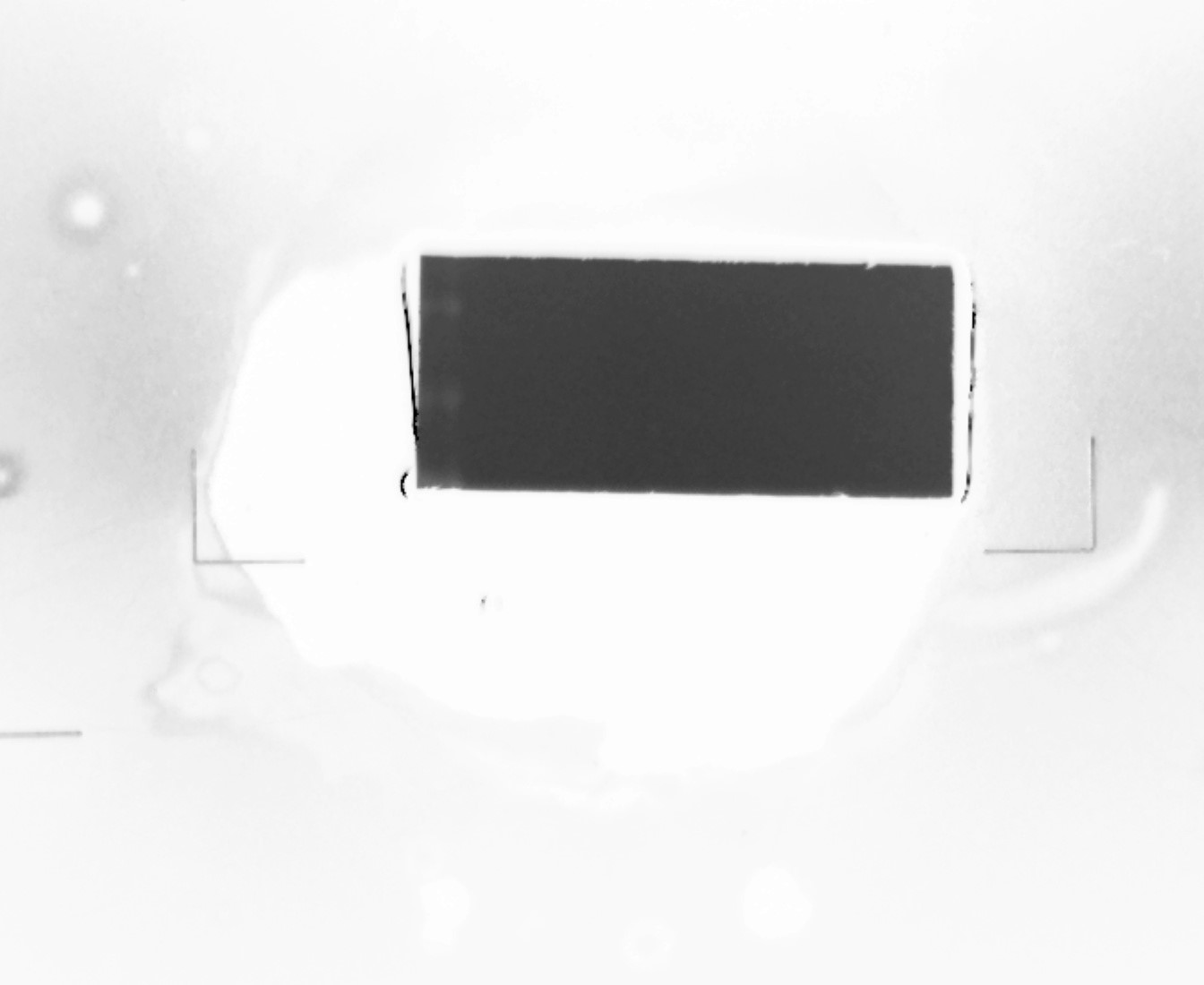

Supplement: Supplemental Material [file KBIE_A_2059614_SM8919.zip › Supplementary Material/Figure 6A/HOS GAPDH-bright field.jpg]

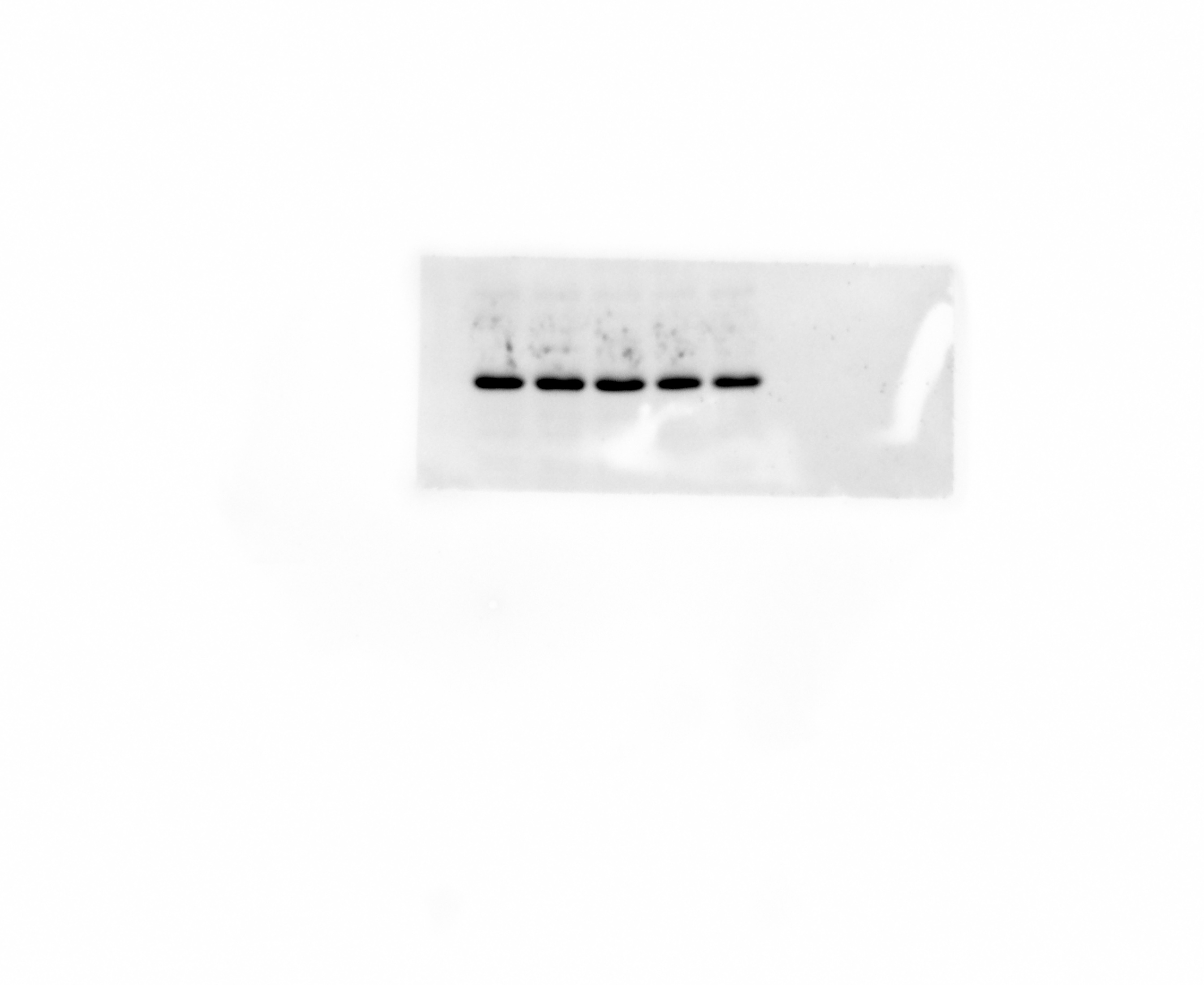

Supplement: Supplemental Material [file KBIE_A_2059614_SM8919.zip › Supplementary Material/Figure 6A/HOS GAPDH.jpg]

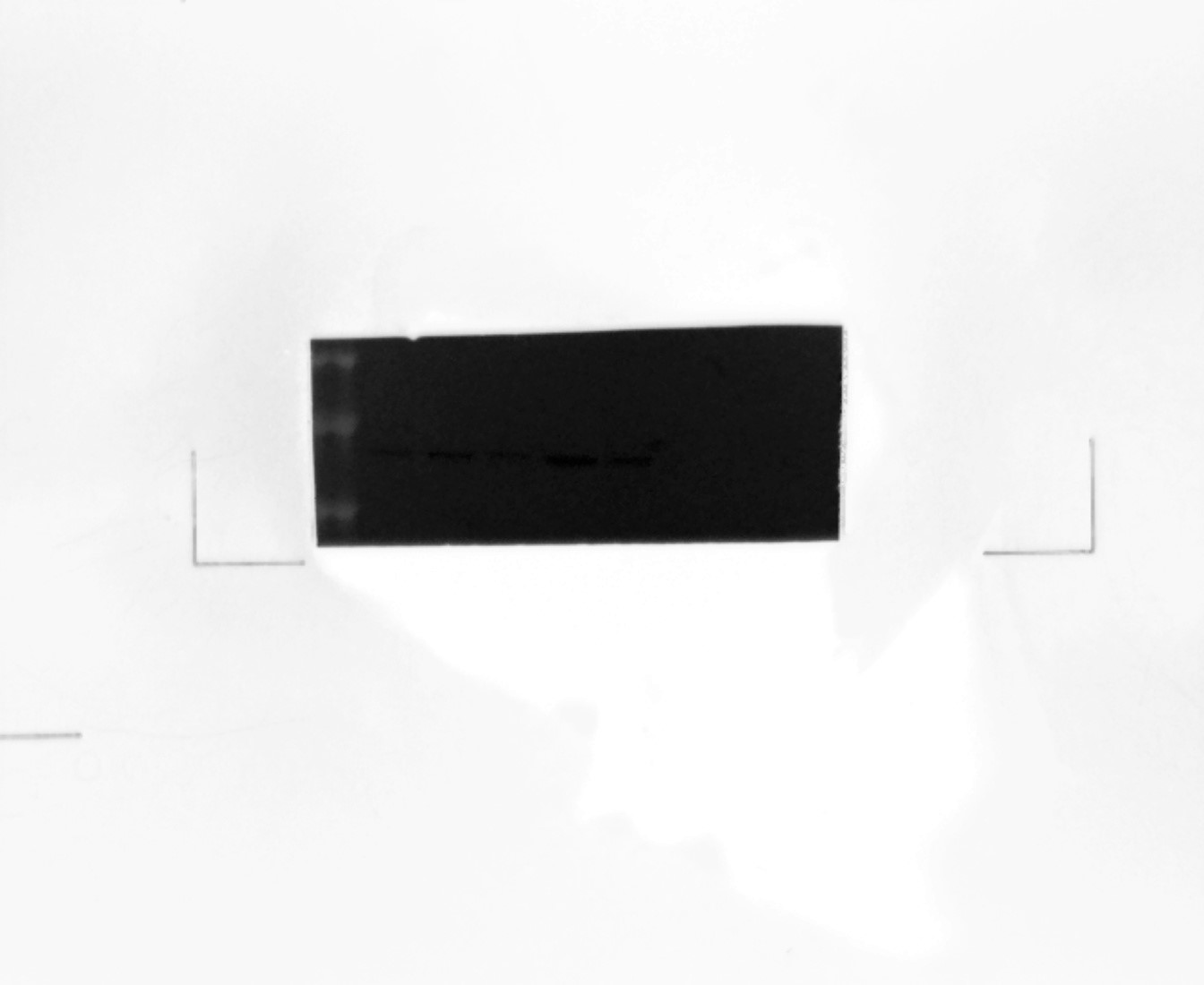

Supplement: Supplemental Material [file KBIE_A_2059614_SM8919.zip › Supplementary Material/Figure 6A/HOS SYK-bright field.jpg]

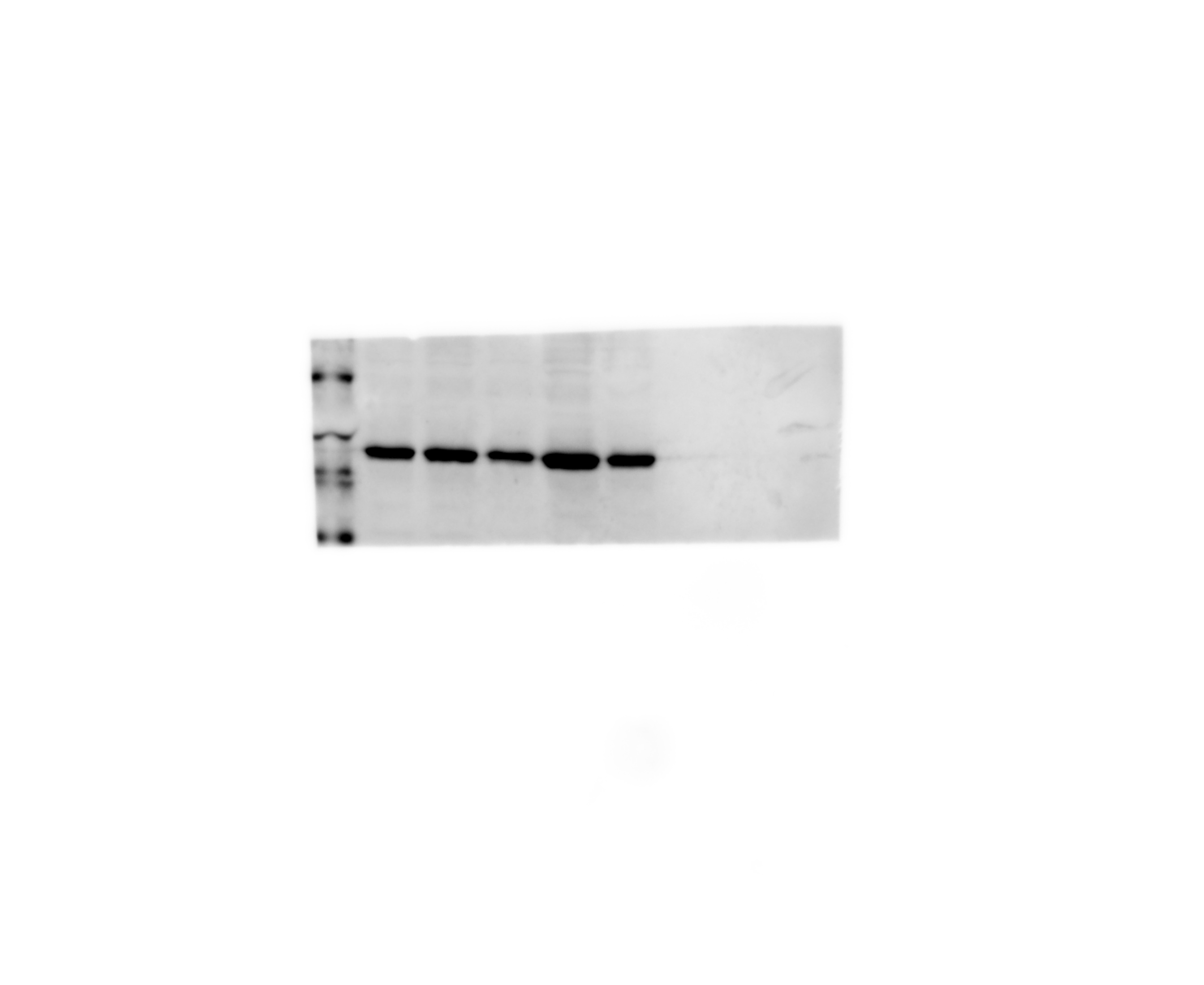

Supplement: Supplemental Material [file KBIE_A_2059614_SM8919.zip › Supplementary Material/Figure 6A/HOS SYK.jpg]

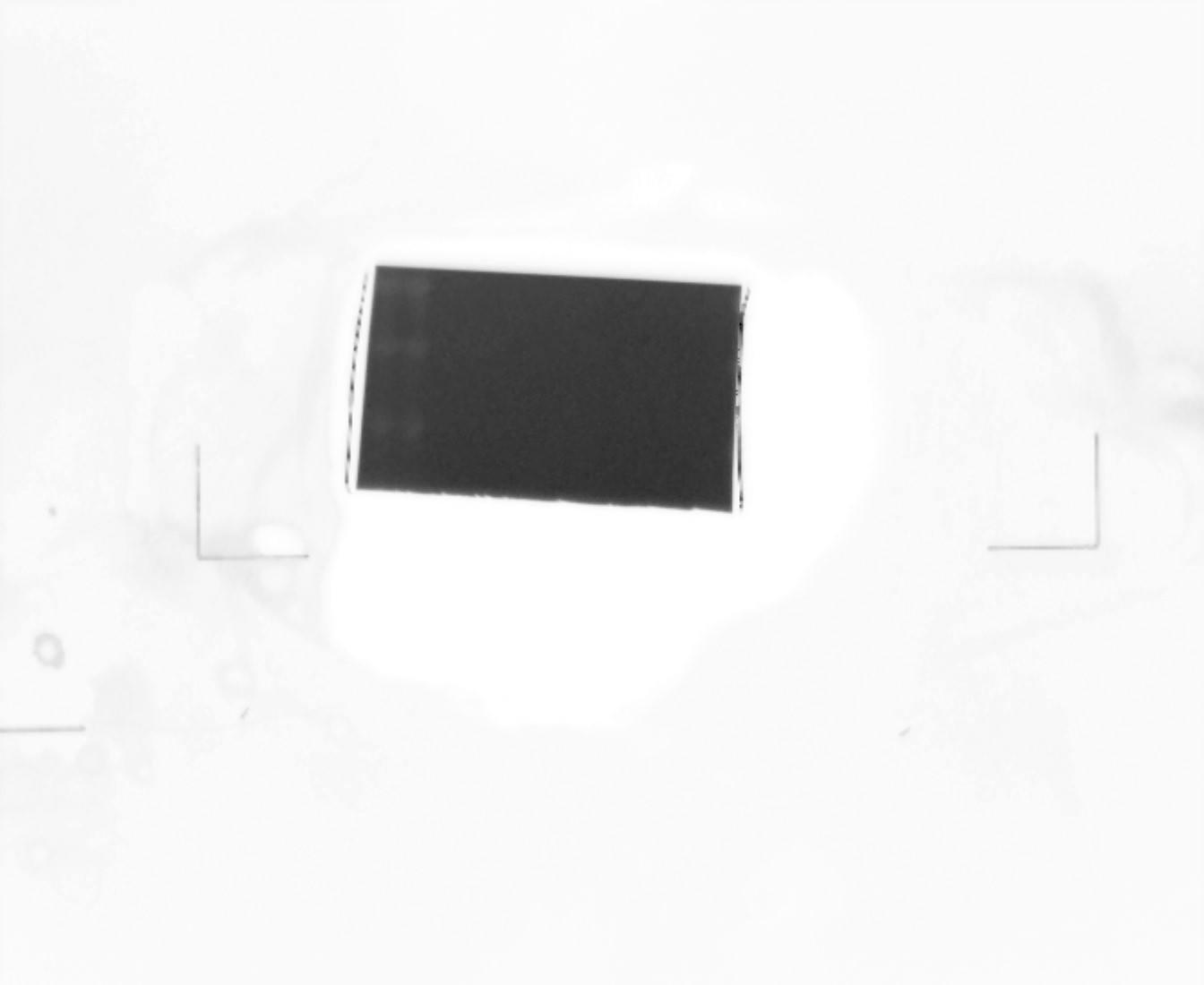

Supplement: Supplemental Material [file KBIE_A_2059614_SM8919.zip › Supplementary Material/Figure 6A/Saos-2 GAPDH-bright field.jpg]

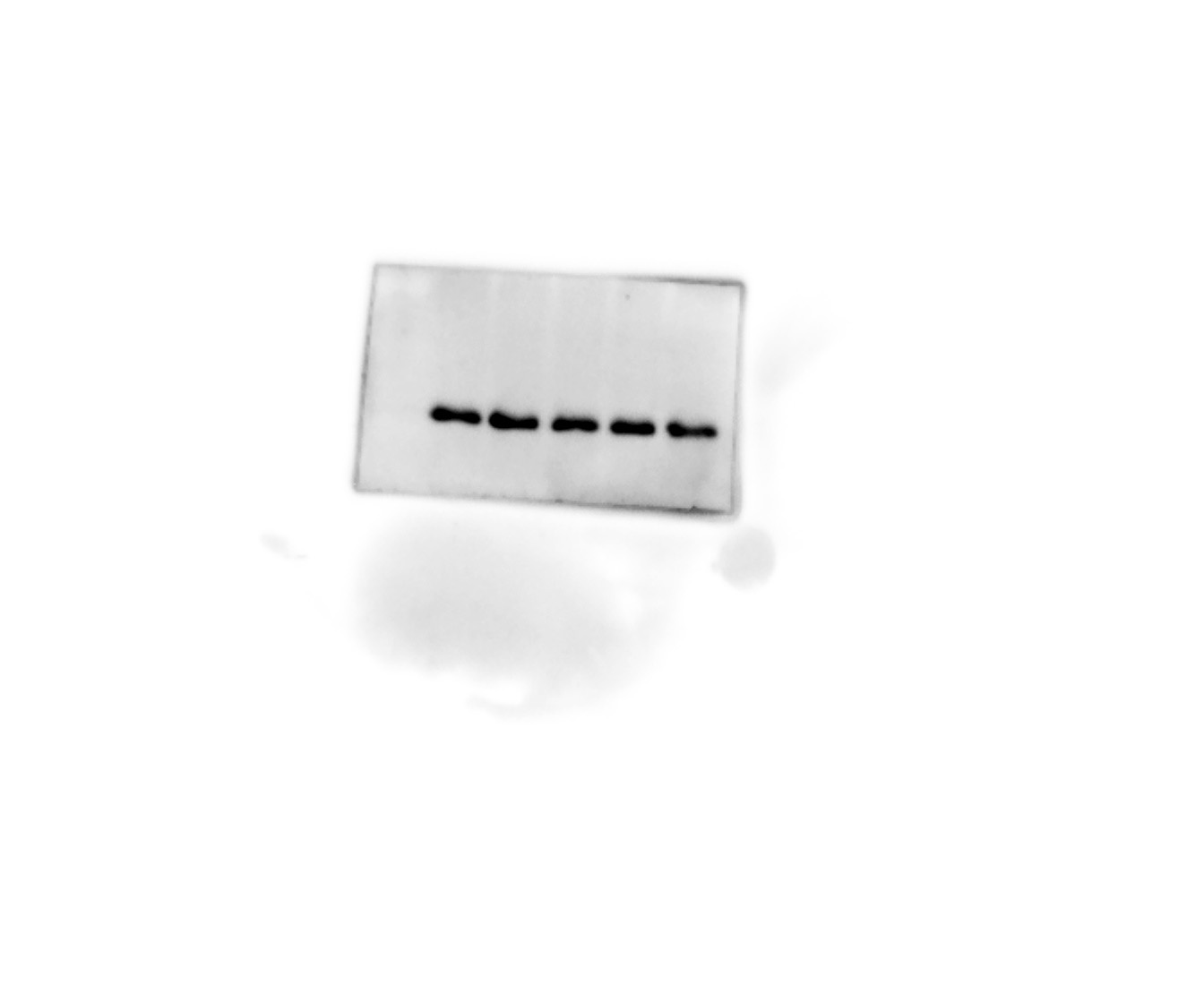

Supplement: Supplemental Material [file KBIE_A_2059614_SM8919.zip › Supplementary Material/Figure 6A/Saos-2 GAPDH.jpg]

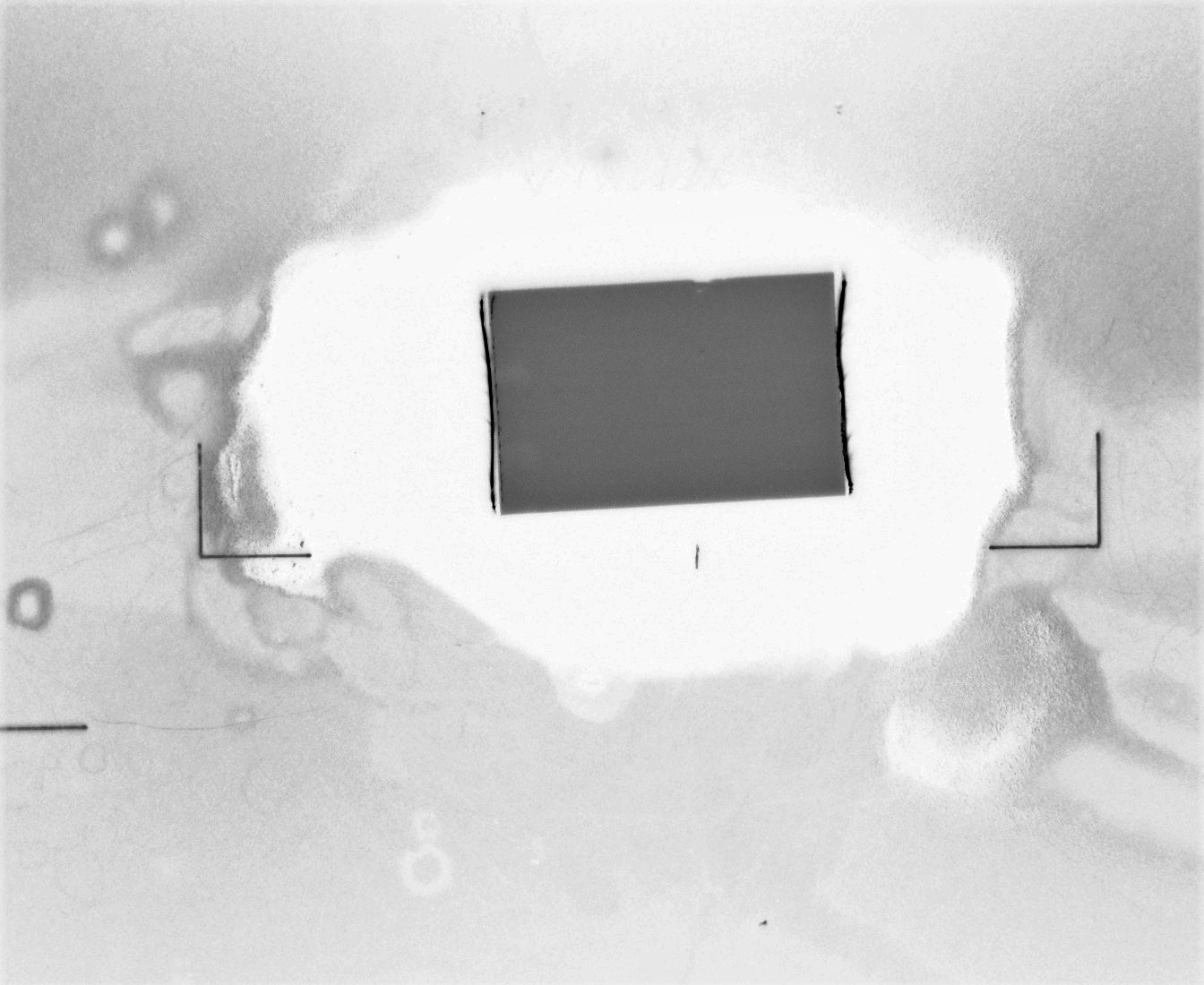

Supplement: Supplemental Material [file KBIE_A_2059614_SM8919.zip › Supplementary Material/Figure 6A/Saos-2 SYK-bright field.jpg]

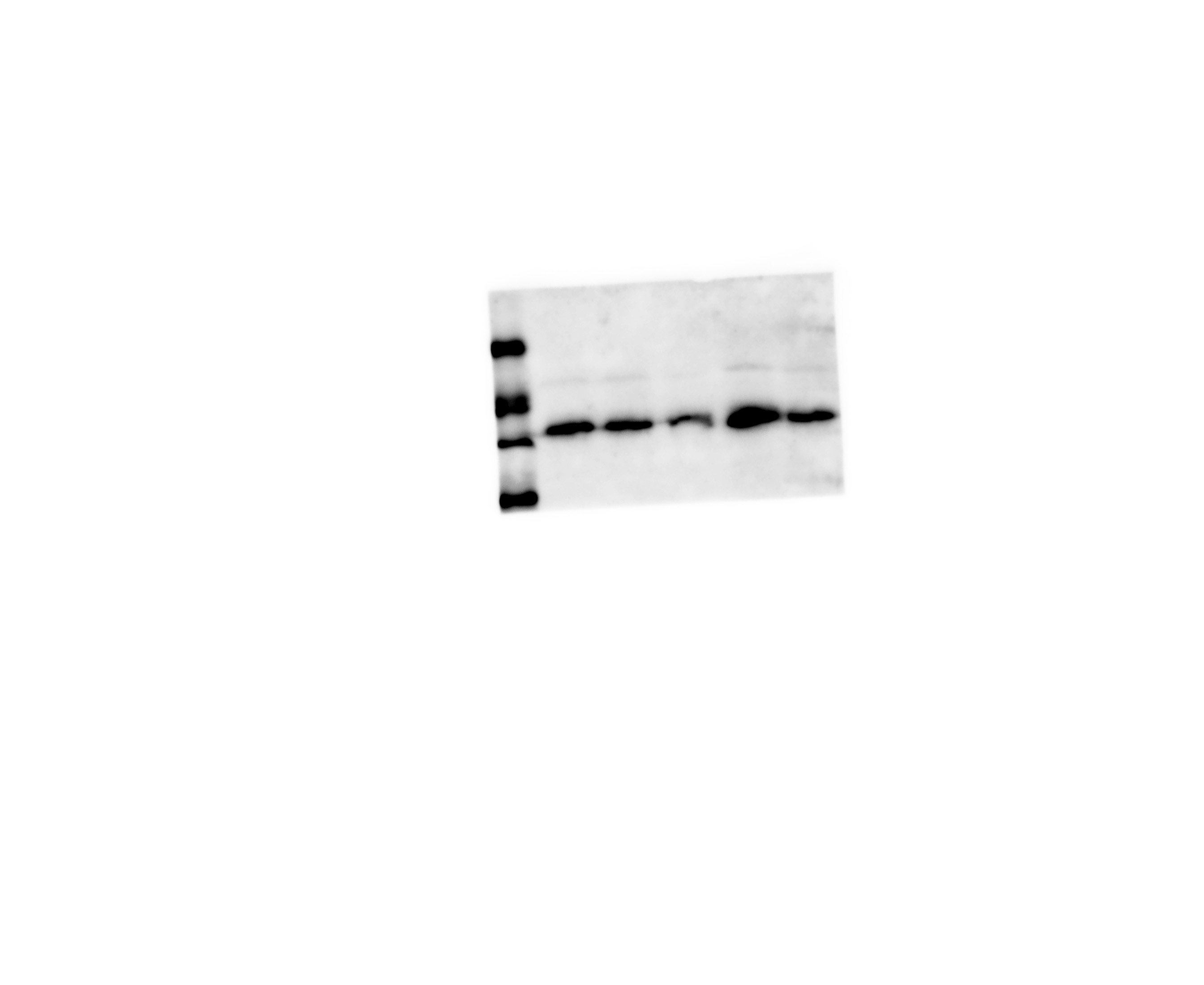

Supplement: Supplemental Material [file KBIE_A_2059614_SM8919.zip › Supplementary Material/Figure 6A/Saos-2 SYK.jpg]

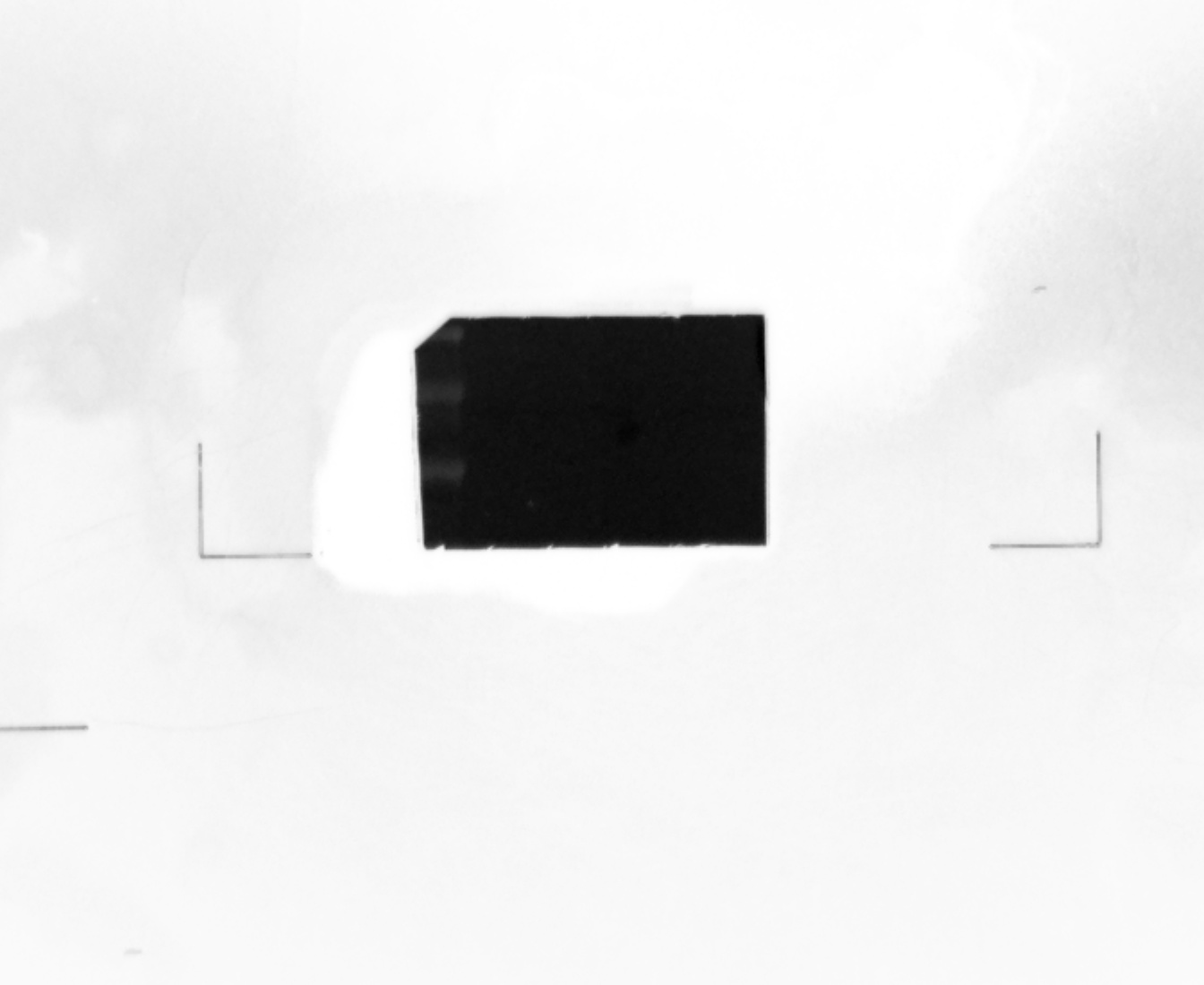

Supplement: Supplemental Material [file KBIE_A_2059614_SM8919.zip › Supplementary Material/Figure 6D/HOS AKT-bright field.jpg]

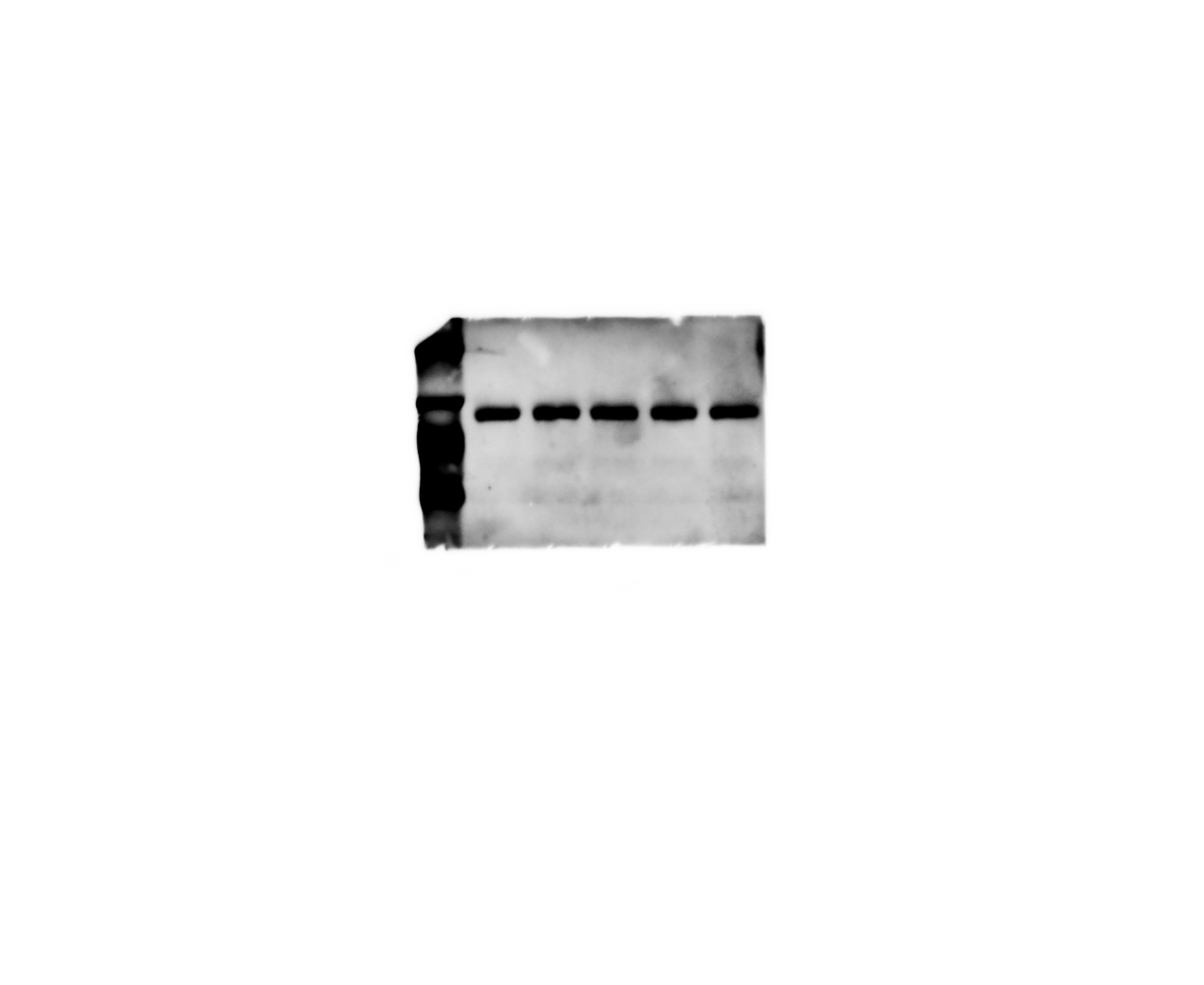

Supplement: Supplemental Material [file KBIE_A_2059614_SM8919.zip › Supplementary Material/Figure 6D/HOS AKT.jpg]

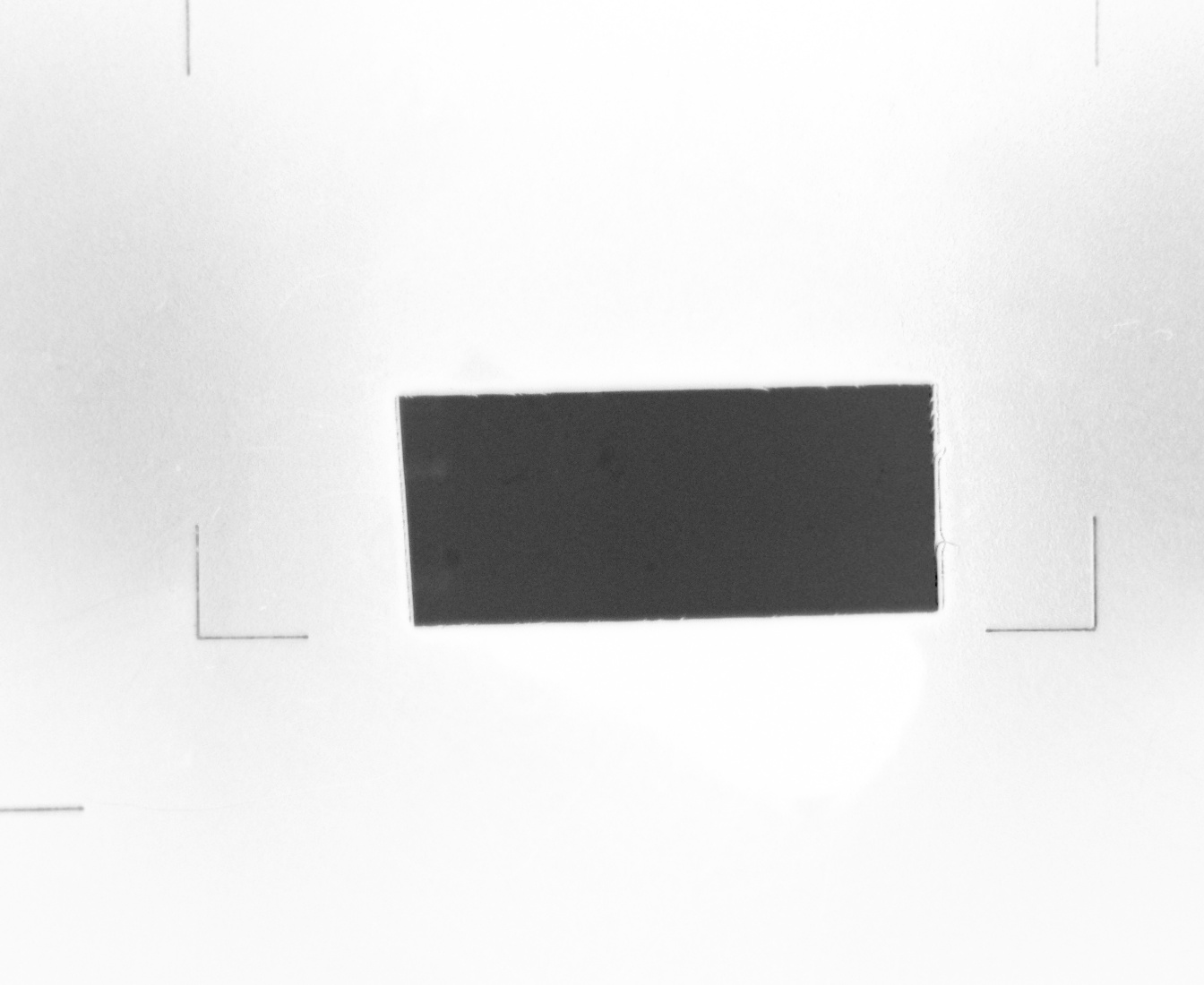

Supplement: Supplemental Material [file KBIE_A_2059614_SM8919.zip › Supplementary Material/Figure 6D/HOS GAPDH-bright field.jpg]

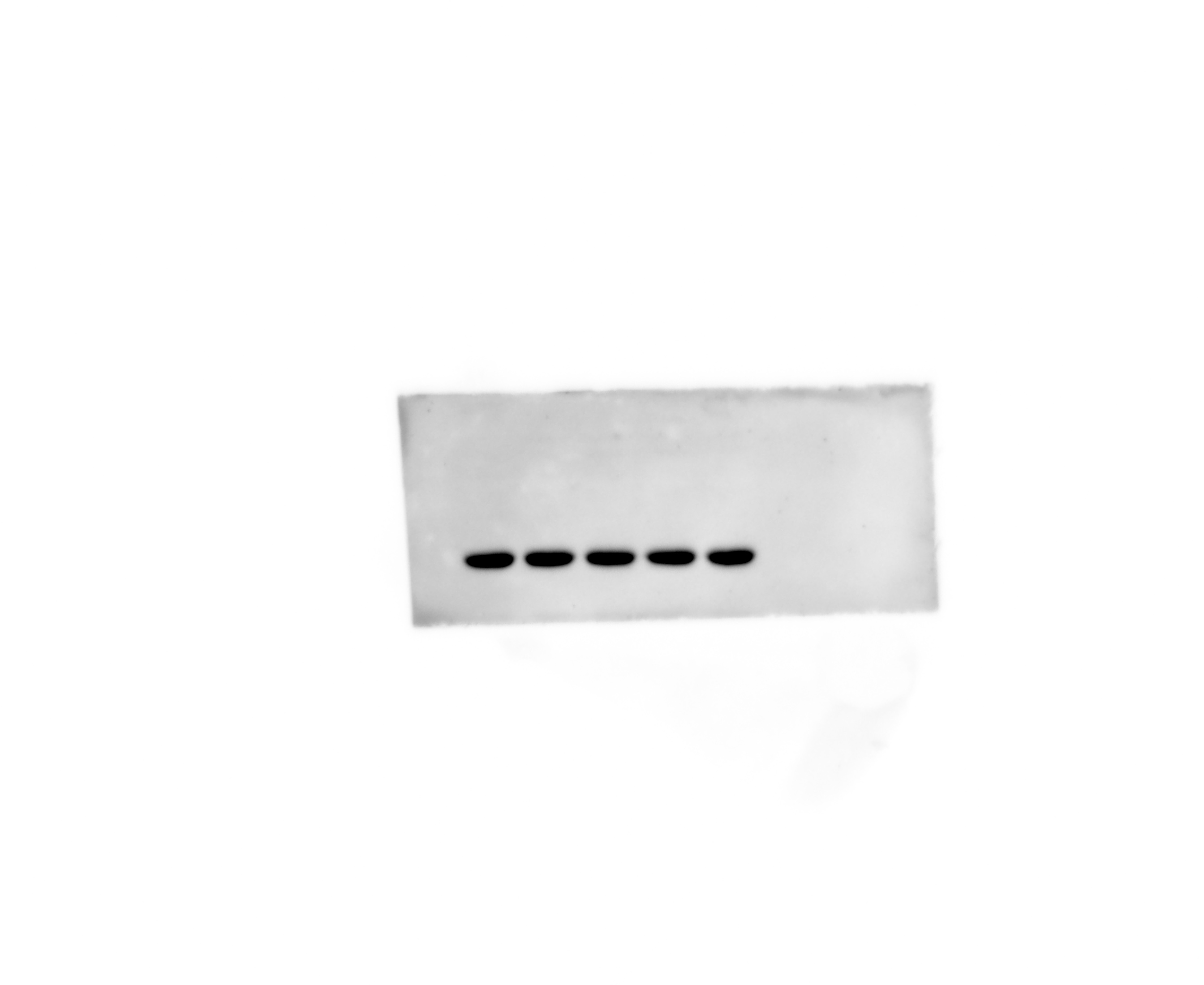

Supplement: Supplemental Material [file KBIE_A_2059614_SM8919.zip › Supplementary Material/Figure 6D/HOS GAPDH.jpg]

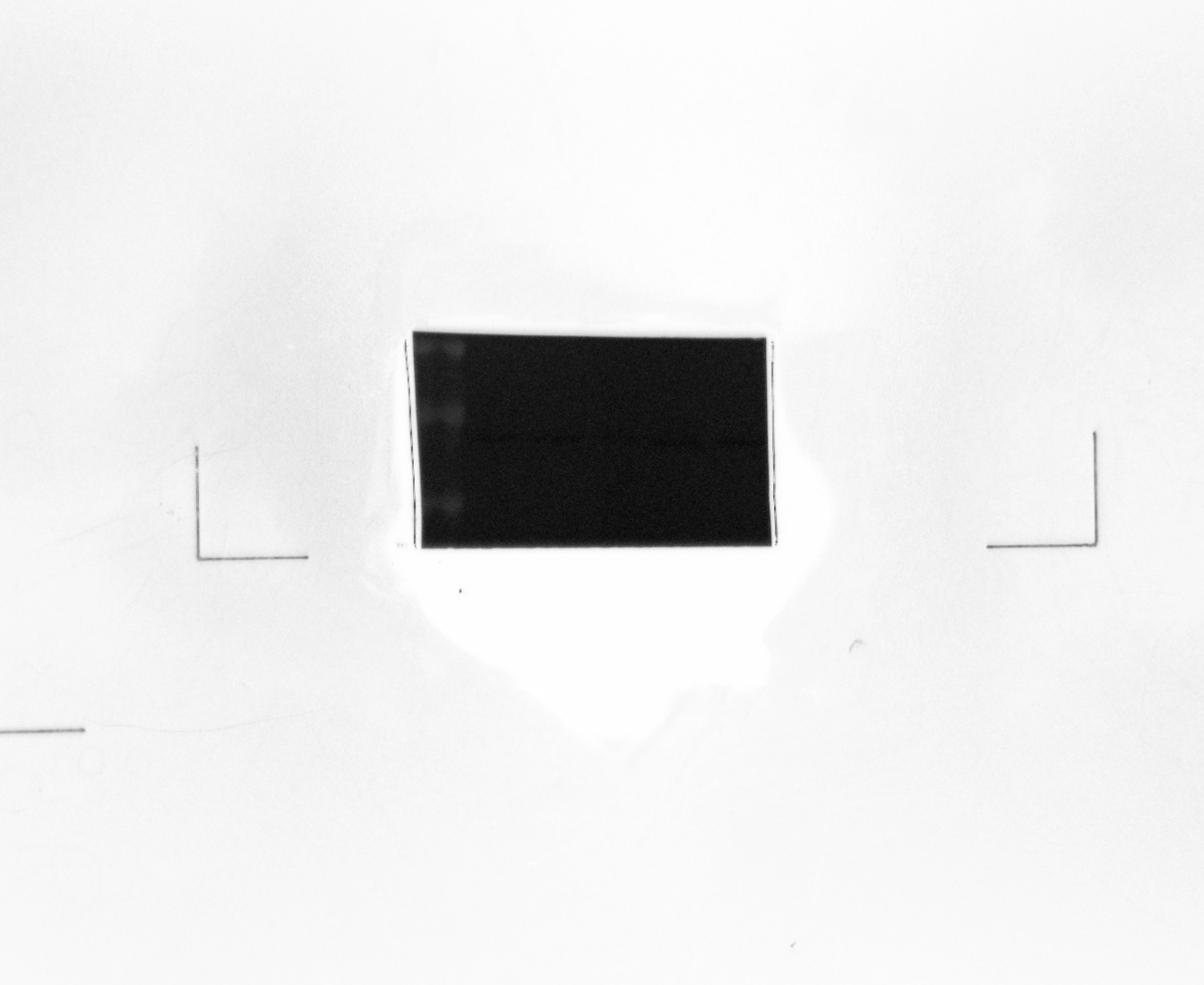

Supplement: Supplemental Material [file KBIE_A_2059614_SM8919.zip › Supplementary Material/Figure 6D/HOS P-AKT-bright field.jpg]

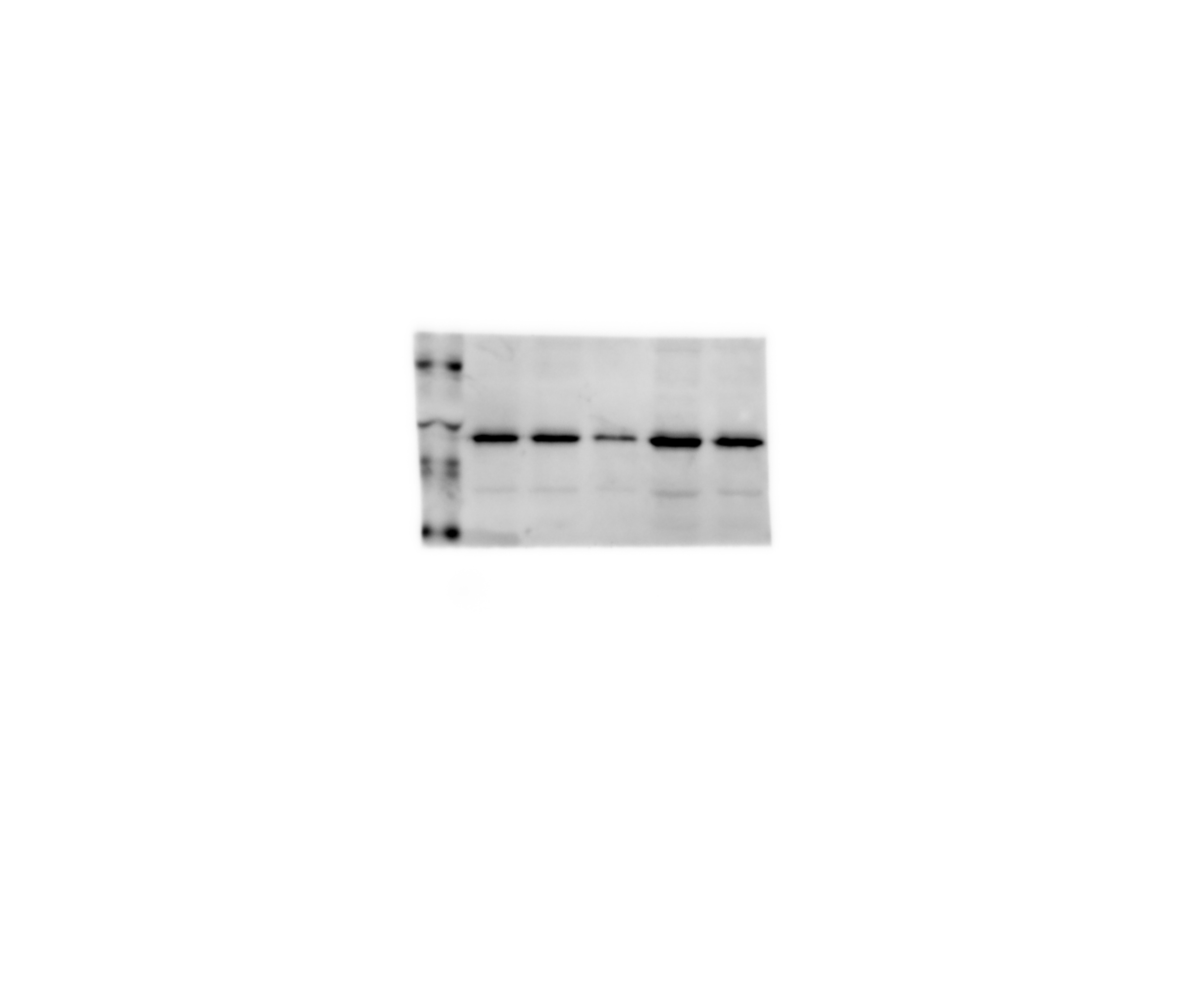

Supplement: Supplemental Material [file KBIE_A_2059614_SM8919.zip › Supplementary Material/Figure 6D/HOS P-AKT.jpg]

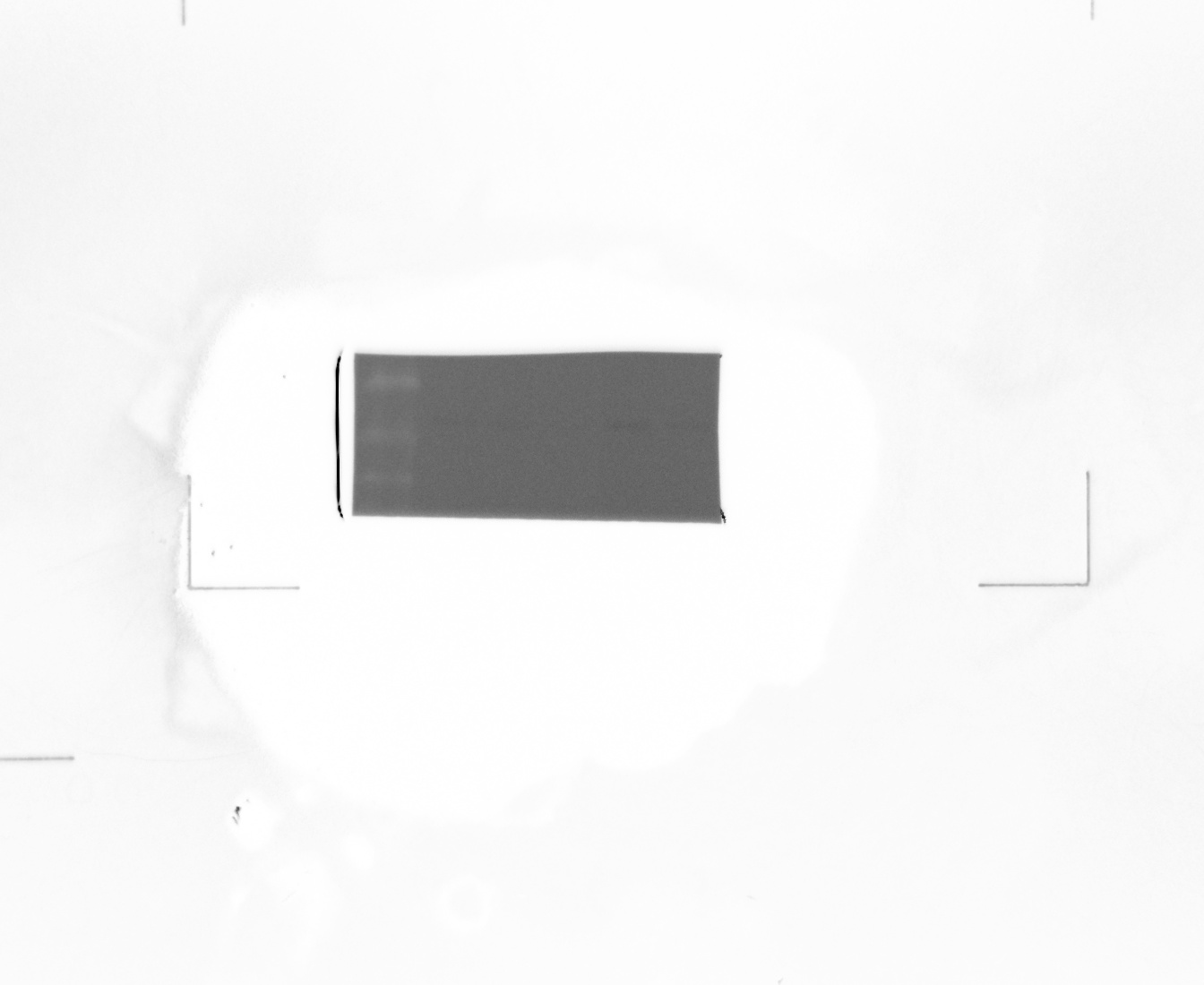

Supplement: Supplemental Material [file KBIE_A_2059614_SM8919.zip › Supplementary Material/Figure 6D/HOS P-PI3K-bright field.jpg]

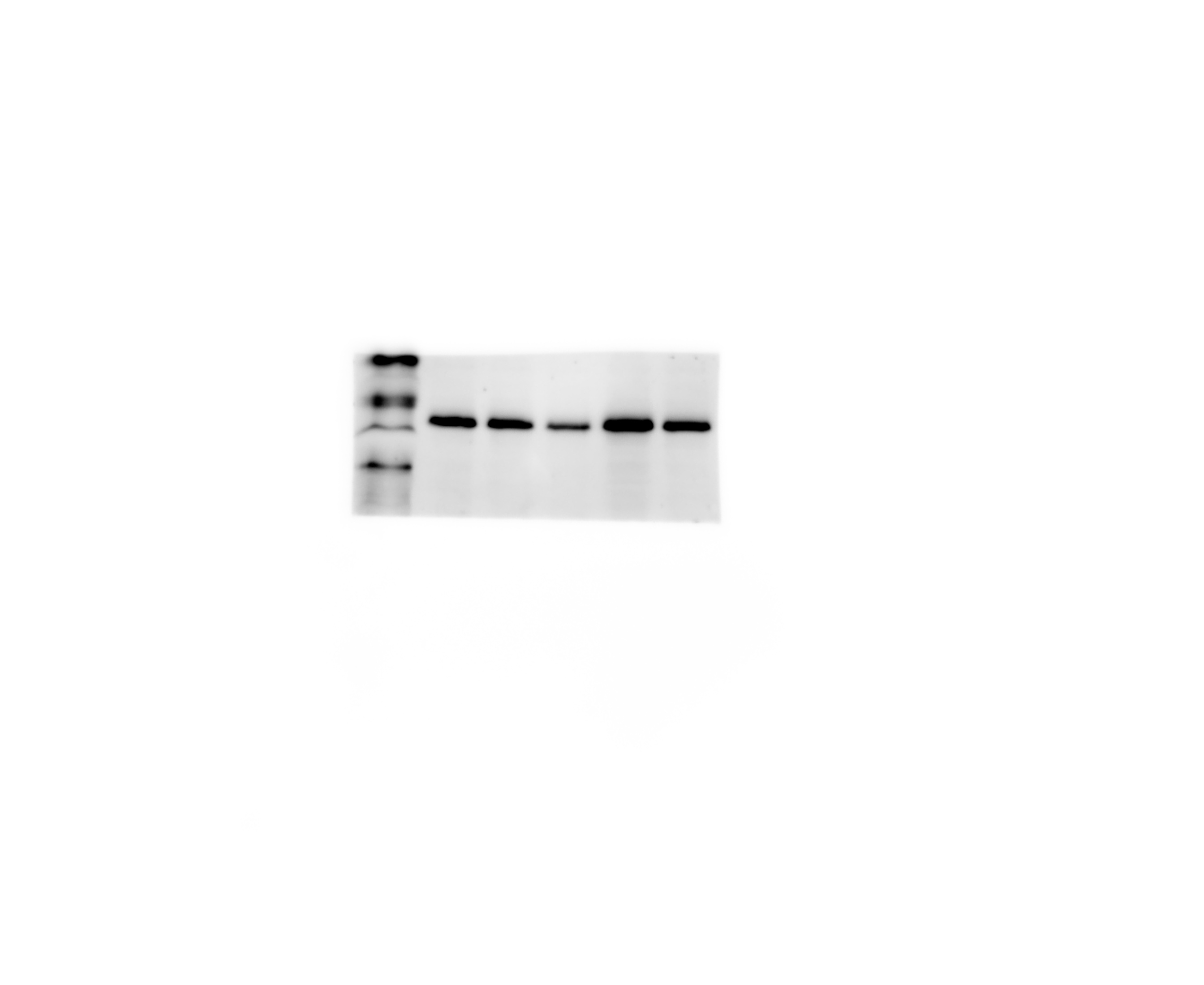

Supplement: Supplemental Material [file KBIE_A_2059614_SM8919.zip › Supplementary Material/Figure 6D/HOS P-PI3K.jpg]

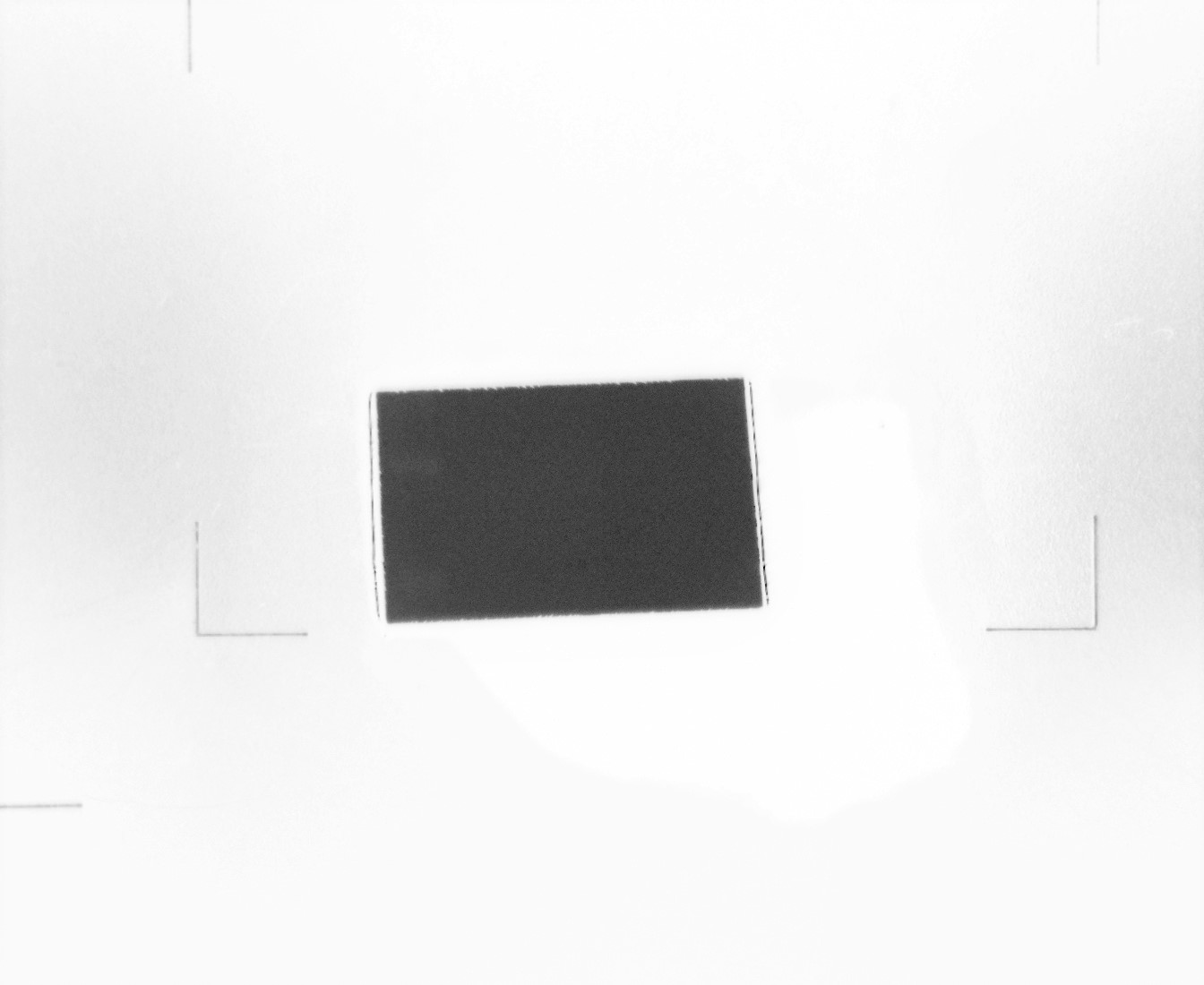

Supplement: Supplemental Material [file KBIE_A_2059614_SM8919.zip › Supplementary Material/Figure 6D/HOS PI3K-bright field.jpg]

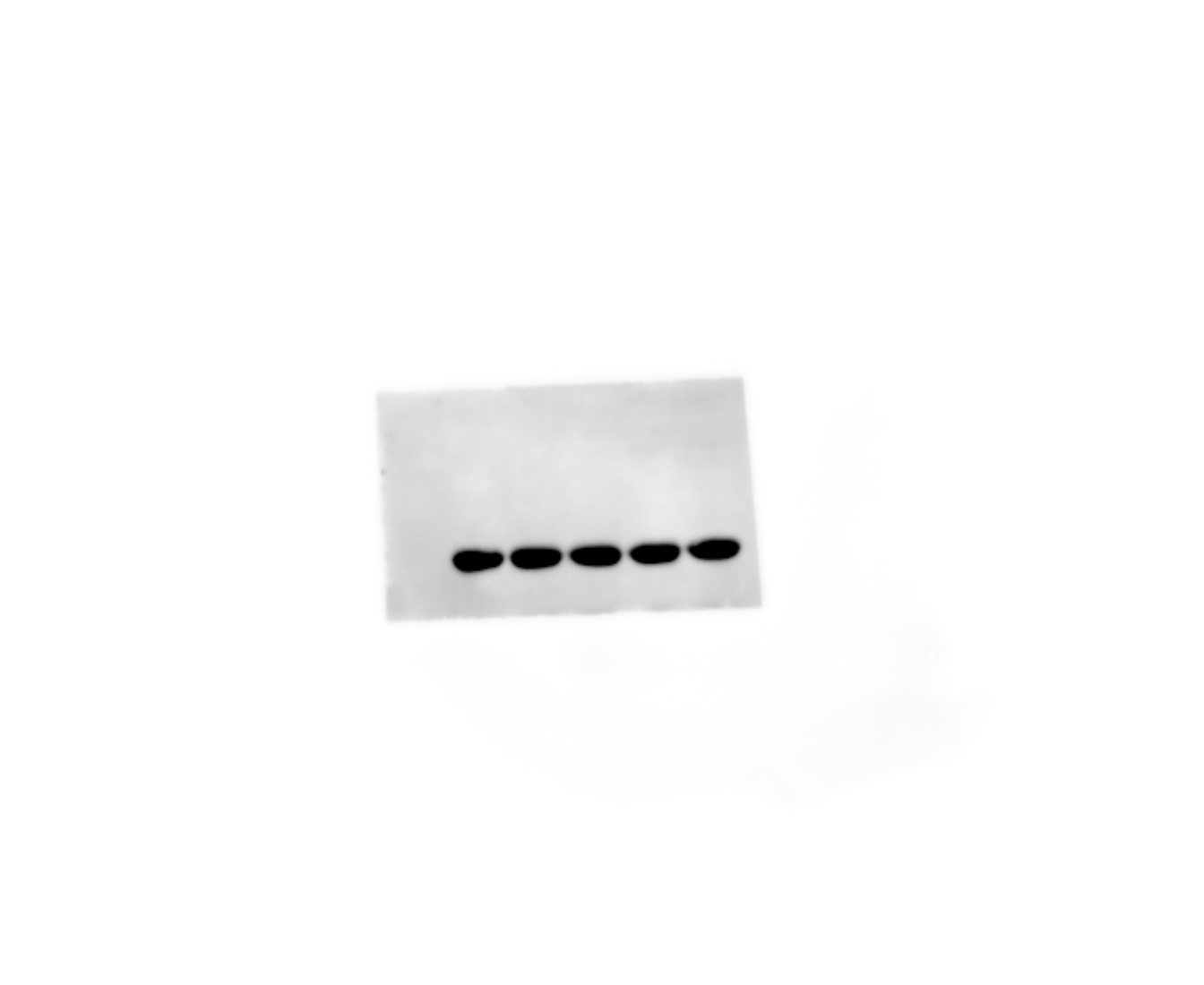

Supplement: Supplemental Material [file KBIE_A_2059614_SM8919.zip › Supplementary Material/Figure 6D/HOS PI3K.jpg]

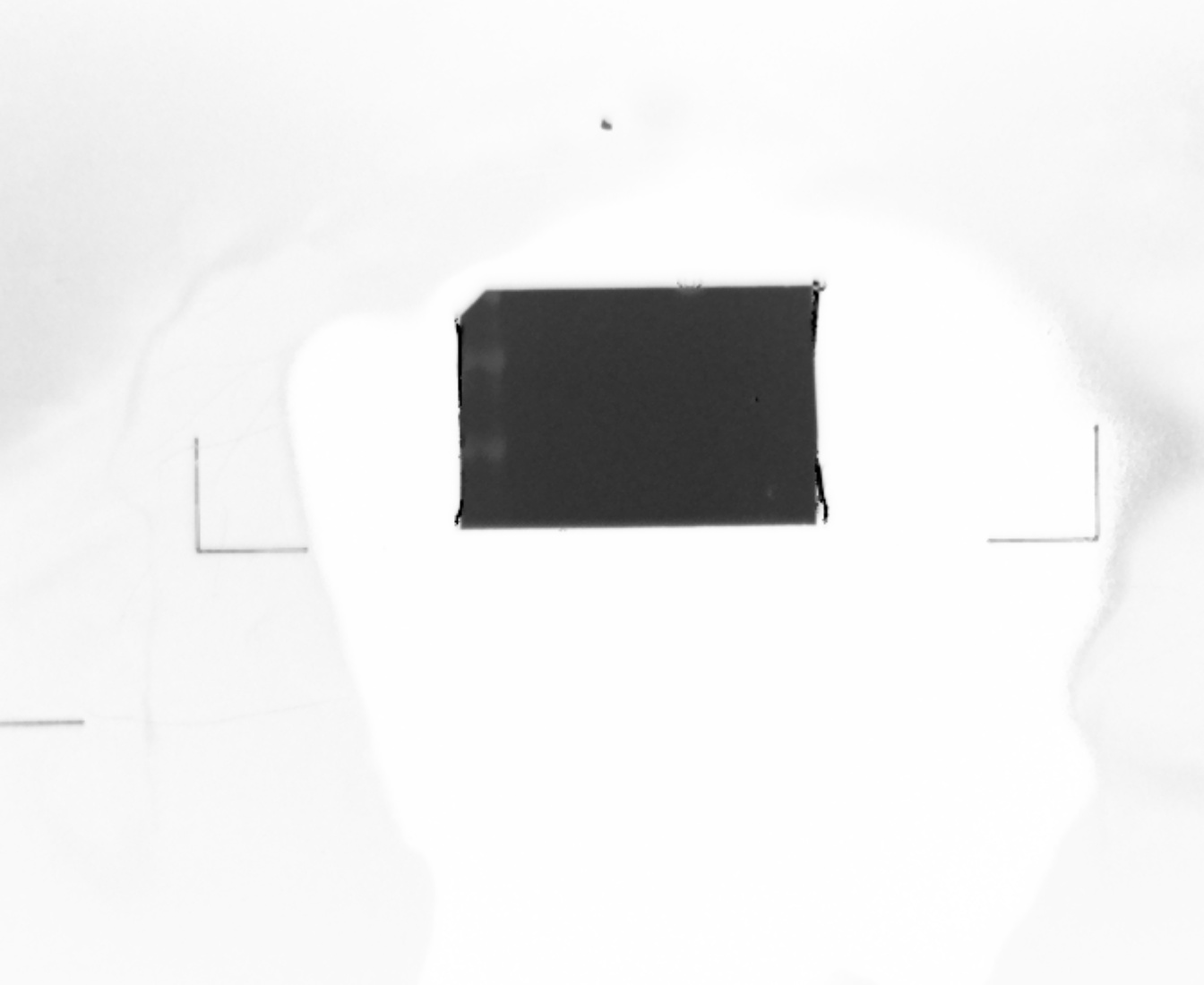

Supplement: Supplemental Material [file KBIE_A_2059614_SM8919.zip › Supplementary Material/Figure 6D/Saos-2 AKT-bright field.jpg]

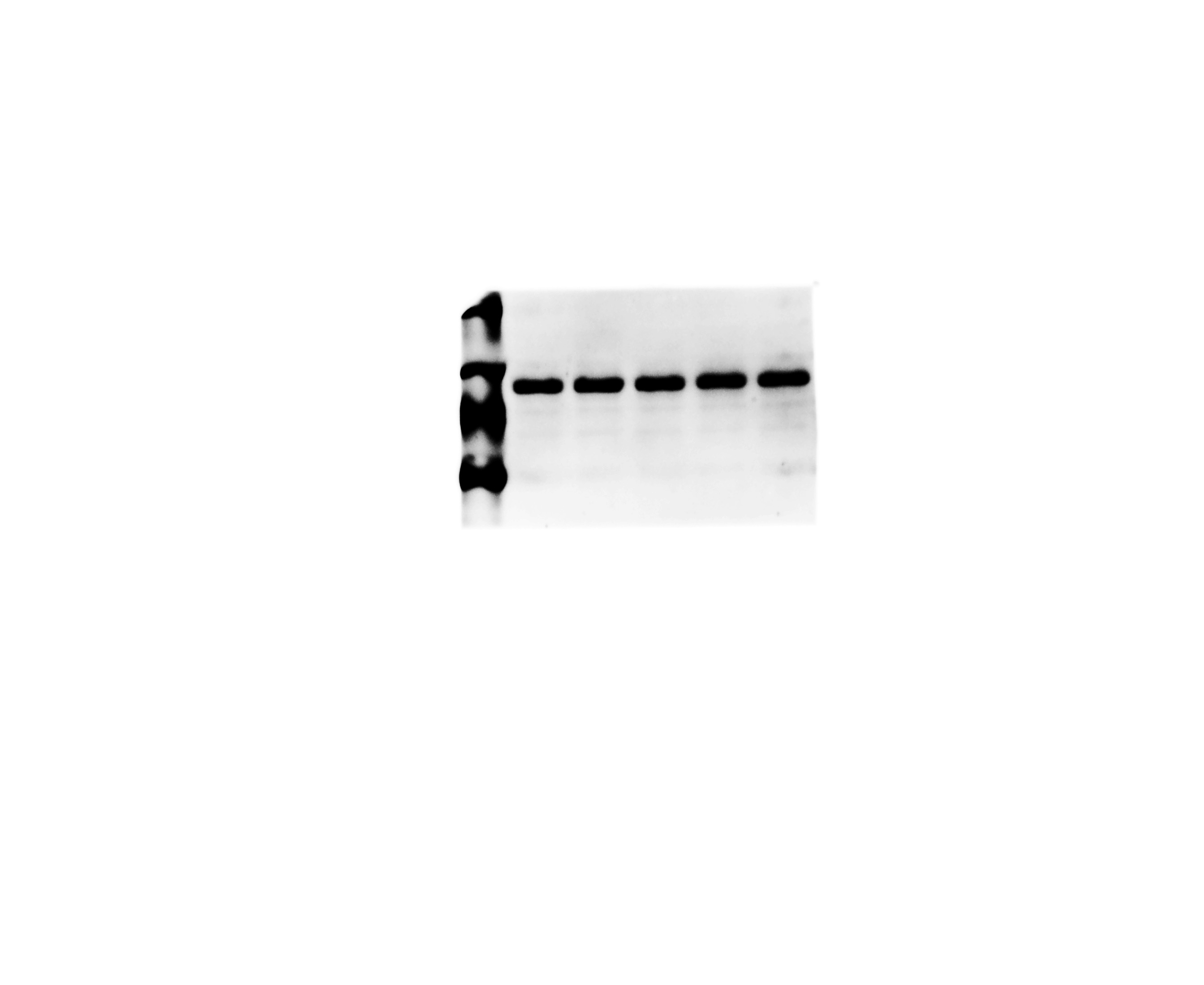

Supplement: Supplemental Material [file KBIE_A_2059614_SM8919.zip › Supplementary Material/Figure 6D/Saos-2 AKT.jpg]

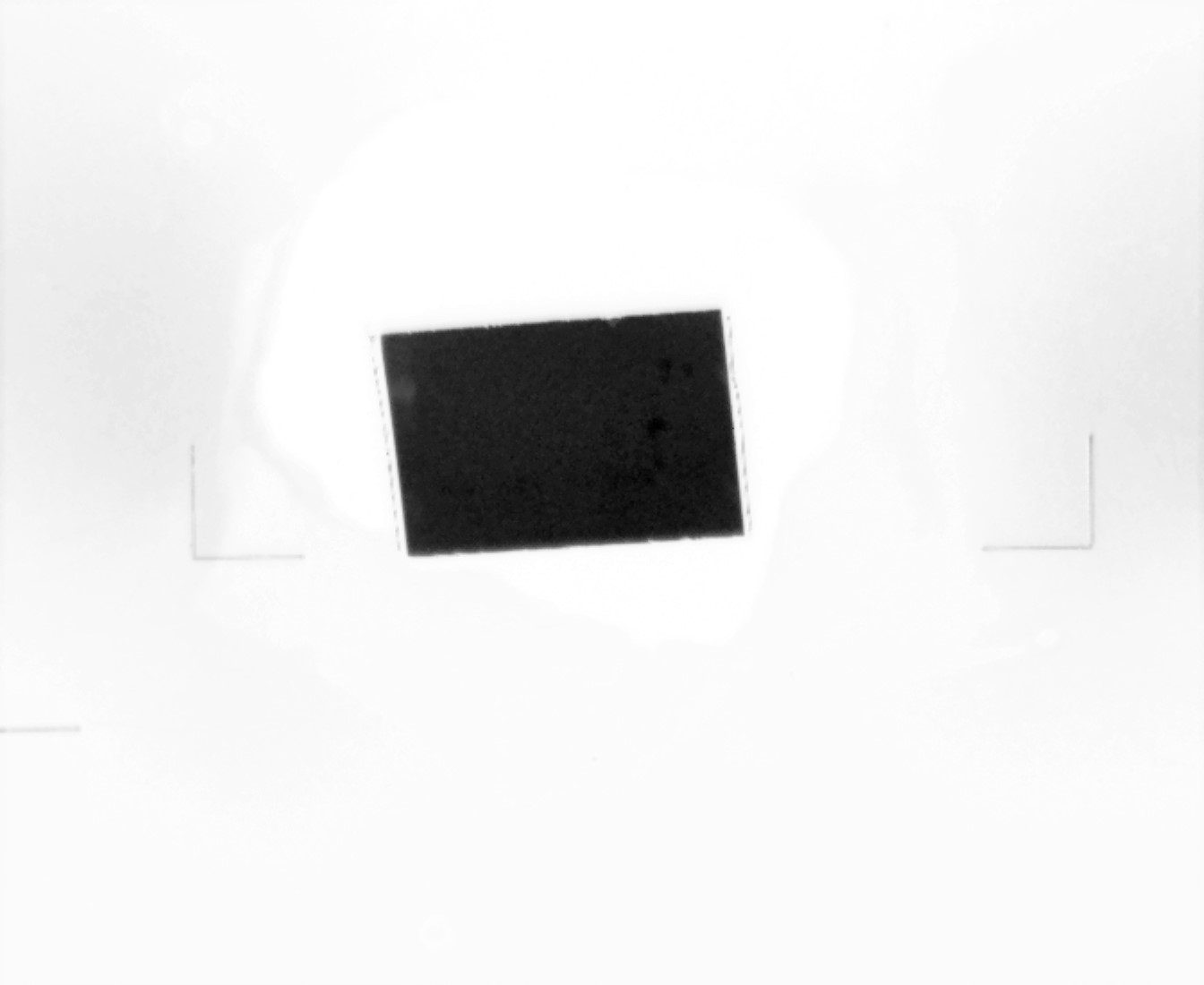

Supplement: Supplemental Material [file KBIE_A_2059614_SM8919.zip › Supplementary Material/Figure 6D/Saos-2 GAPDH-bright field.jpg]

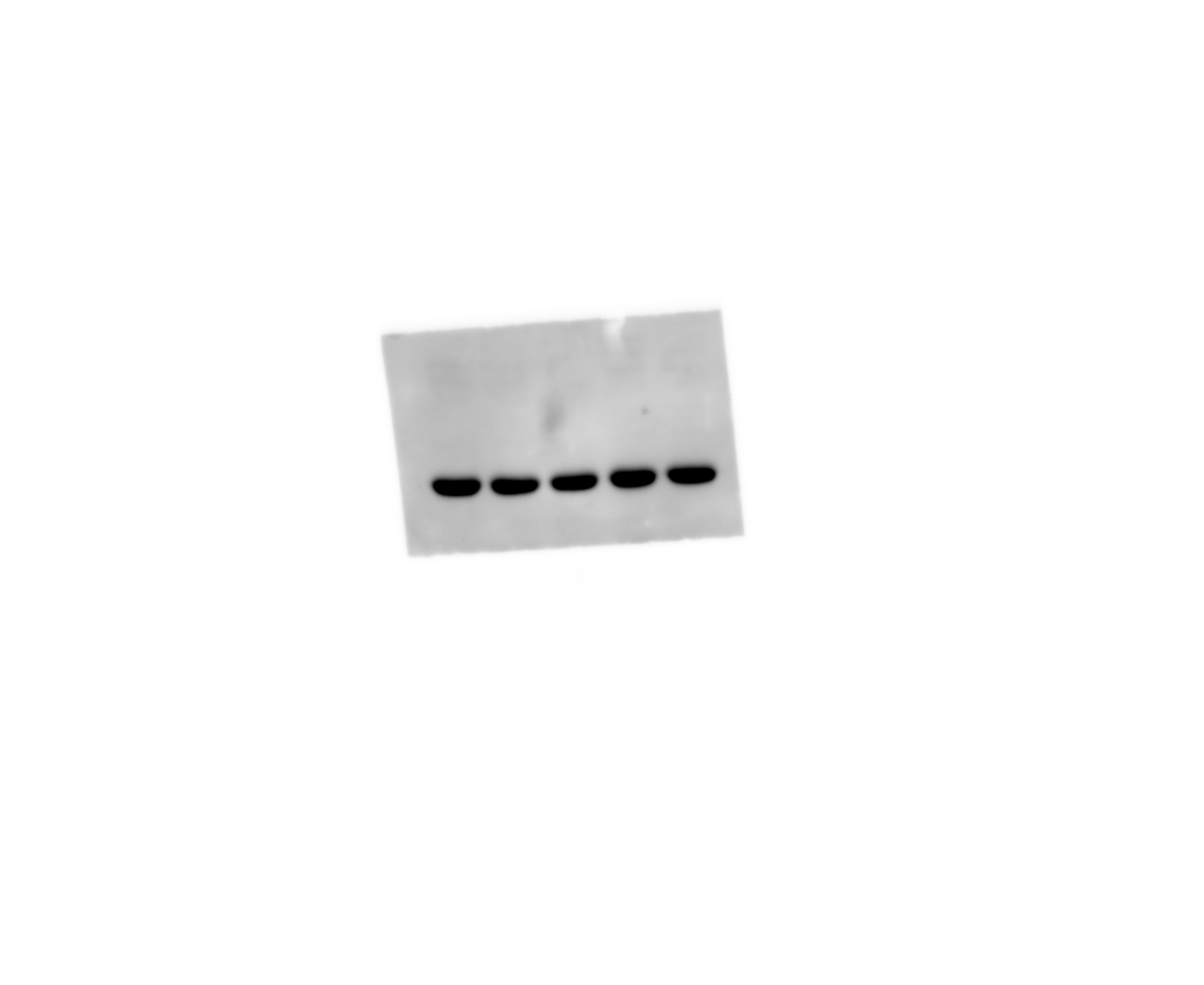

Supplement: Supplemental Material [file KBIE_A_2059614_SM8919.zip › Supplementary Material/Figure 6D/Saos-2 GAPDH.jpg]

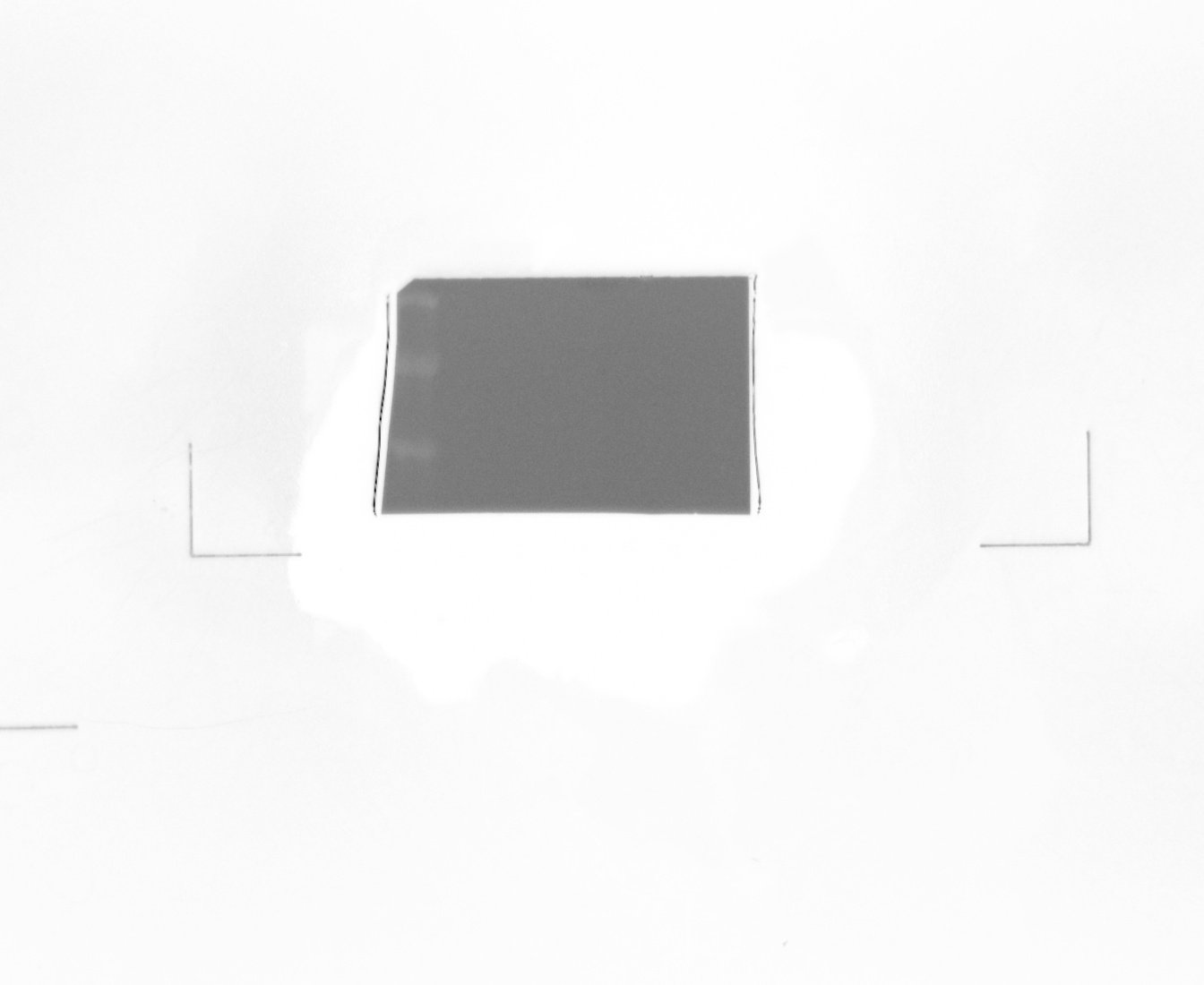

Supplement: Supplemental Material [file KBIE_A_2059614_SM8919.zip › Supplementary Material/Figure 6D/Saos-2 P-AKT-bright field.jpg]

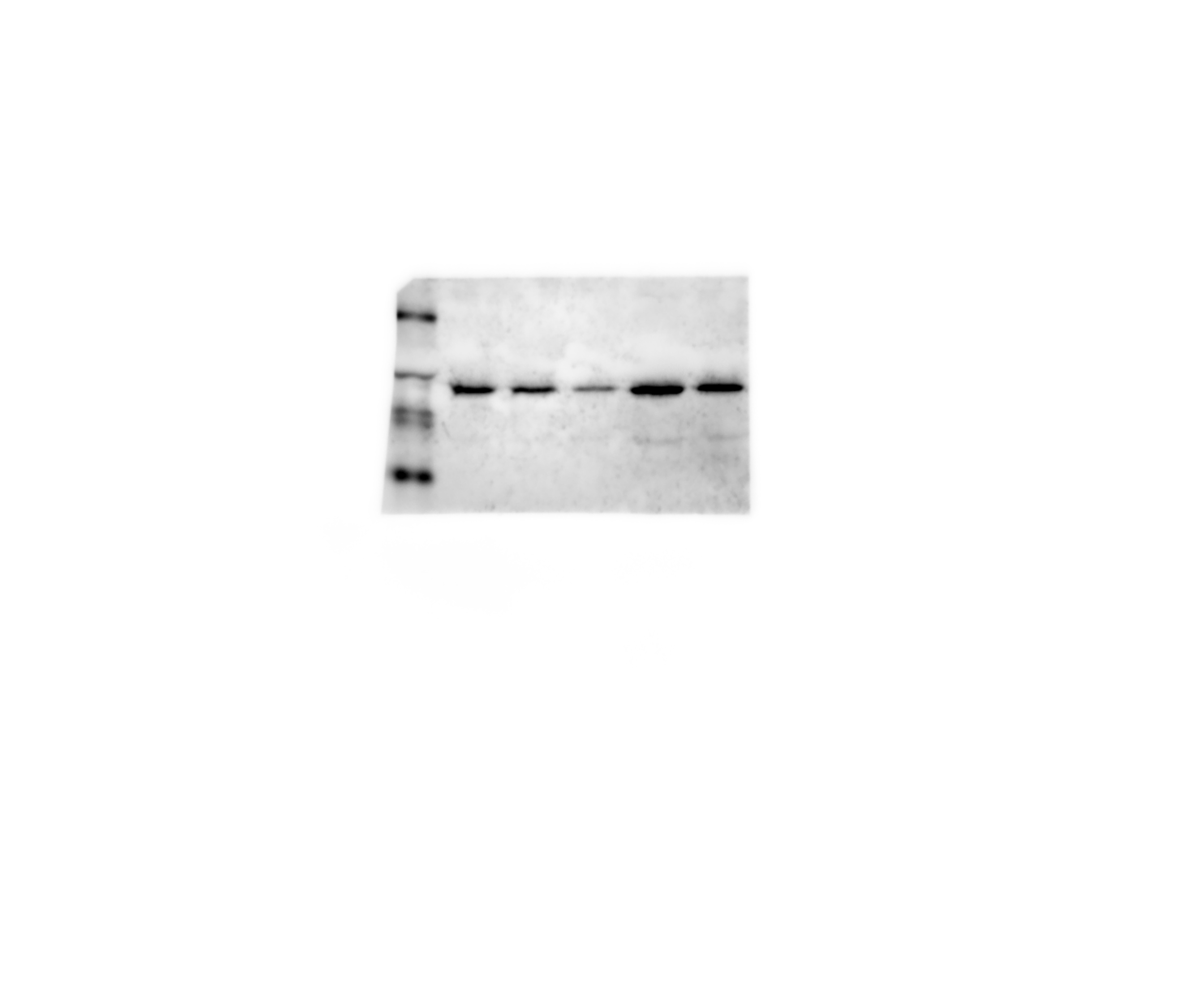

Supplement: Supplemental Material [file KBIE_A_2059614_SM8919.zip › Supplementary Material/Figure 6D/Saos-2 P-AKT.jpg]

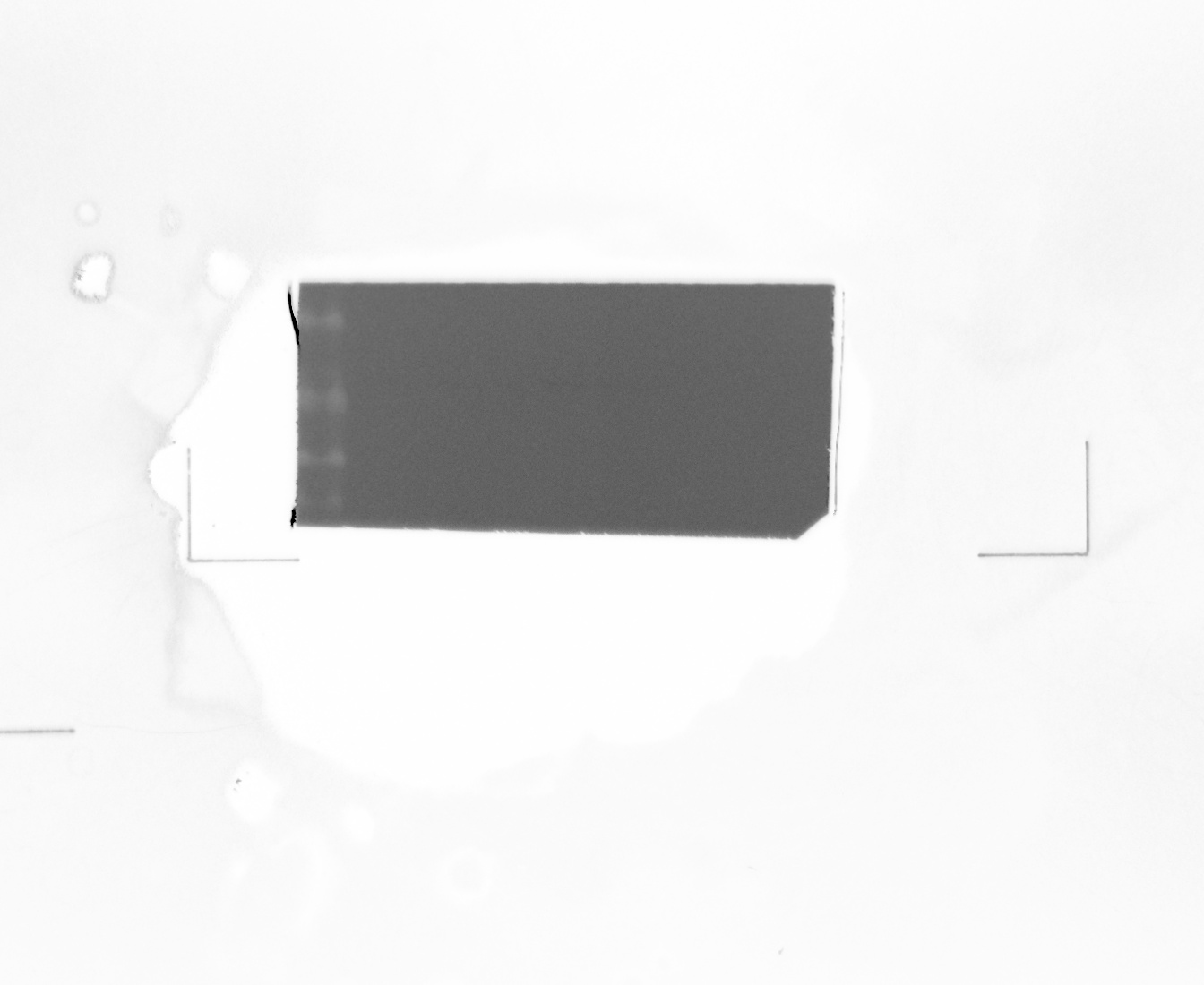

Supplement: Supplemental Material [file KBIE_A_2059614_SM8919.zip › Supplementary Material/Figure 6D/Saos-2 P-PI3K-bright field.jpg]

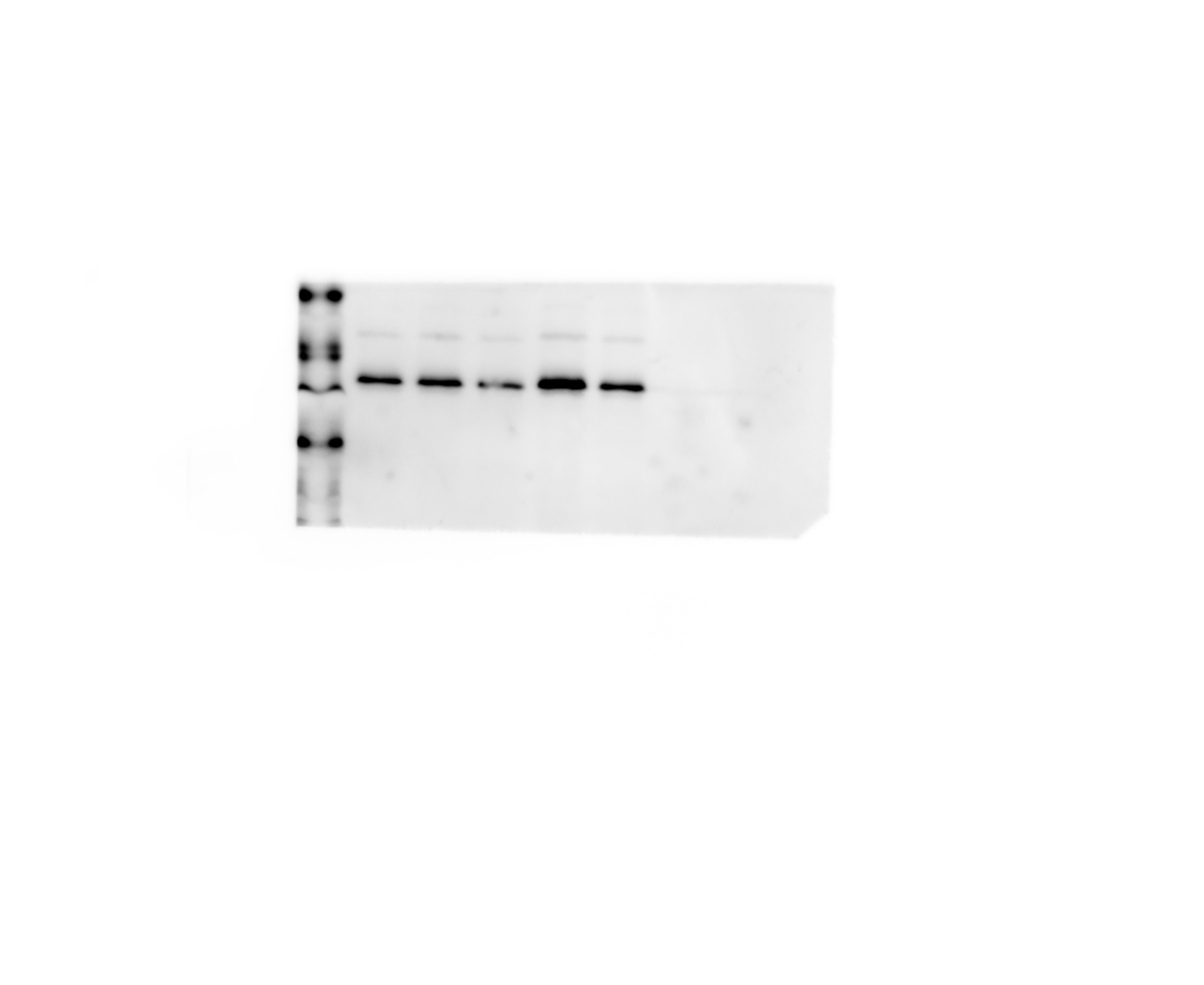

Supplement: Supplemental Material [file KBIE_A_2059614_SM8919.zip › Supplementary Material/Figure 6D/Saos-2 P-PI3K.jpg]

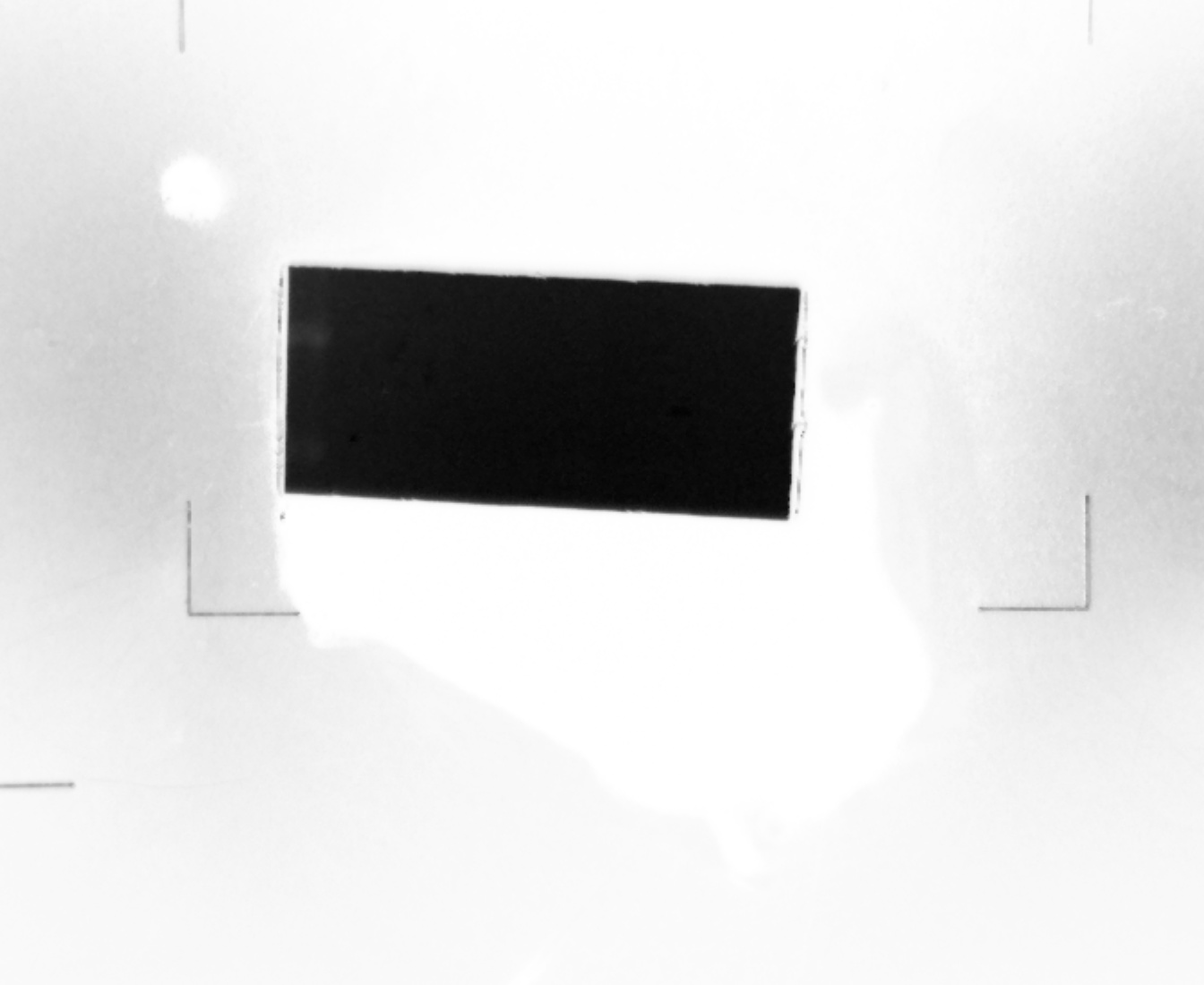

Supplement: Supplemental Material [file KBIE_A_2059614_SM8919.zip › Supplementary Material/Figure 6D/Saos-2 PI3K-bright field.jpg]

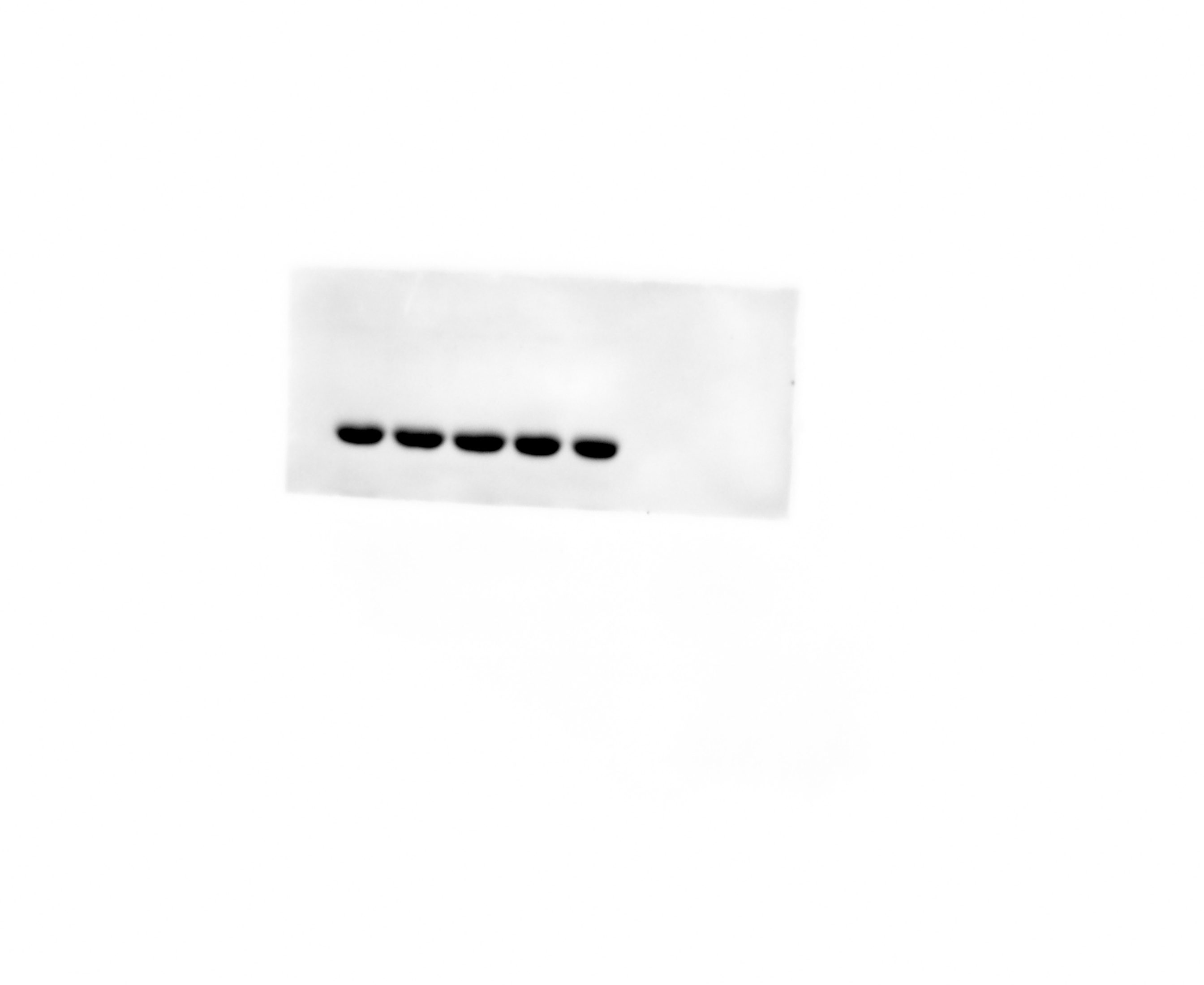

Supplement: Supplemental Material [file KBIE_A_2059614_SM8919.zip › Supplementary Material/Figure 6D/Saos-2 PI3K.jpg]
